# Peer Navigator Intervention and Opioid-Related Adverse Events for Emergency Department Patients: A Randomized Clinical Trial

**DOI:** 10.1001/jamanetworkopen.2025.55903

**Published:** 2026-02-06

**Authors:** Kelly M. Doran, Alice E. Welch, Kelsey L. Kepler, Angela Jeffers, Dominique Chambless, Ethan Cowan, Ian Wittman, Angela Regina, Katherine Siu, Veronika S. Bailey, Yasna Rostam-Abadi, Joseph Kennedy, Hillary V. Kunins, Marya Gwadz, Donna Shelley, Charles M. Cleland, Jennifer McNeely

**Affiliations:** 1 Department of Emergency Medicine, NYU Grossman School of Medicine, New York; 2 Department of Population Health, NYU Grossman School of Medicine, New York; 3 New York City Department of Health and Mental Hygiene, New York; 4 Department of Emergency Medicine, Icahn School of Medicine at Mount Sinai, New York, New York; 5 Now with Department of Emergency Medicine, Rutgers New Jersey Medical School, Newark; 6 Department of Emergency Medicine, St Barnabas Hospital, New York, New York; 7 Department of Psychiatry, Yale University, New Haven, Connecticut; 8 Now with San Francisco Department of Public Health, San Francisco, California; 9 Silver School of Social Work, New York University, New York; 10 School of Global Public Health, New York University, New York

## Abstract

**Importance:**

Emergency departments (EDs) serve patients at high risk for overdose. There is increasing interest in peer-delivered ED interventions for substance use but little rigorous research on their effectiveness.

**Objective:**

To examine the effectiveness of an initiative (Relay) operated by the New York City Health Department that dispatches trained peer wellness advocates (WAs) to support ED patients after a nonfatal opioid overdose.

**Design, Setting, and Participants:**

This randomized clinical trial compared Relay and site-directed care (SDC) at 4 EDs in New York, New York. Adult patients presenting after opioid-involved overdose were enrolled from October 6, 2020, to June 30, 2022, with 12 months of outcome follow-up. Statistical analysis was performed from November 4, 2024, to May 6, 2025.

**Intervention:**

ED workers (generally physicians) called the Relay hotline for patients presenting after a suspected opioid-involved overdose. WAs met patients in the ED to provide peer support and brief overdose risk reduction education. WAs attempted to contact patients for 90 days to provide ongoing support, education, and referrals using a harm reduction framework.

**Main Outcomes and Measures:**

Opioid-related adverse events (any opioid-involved overdose [fatal or nonfatal] or any other substance use–related ED visit) in the 12 months after enrollment were identified using health care administrative data plus self-report.

**Results:**

Among a total of 253 participants randomized, 127 were randomized to the Relay arm and 126 to the SDC arm. A total of 247 participants, 125 in the Relay arm and 122 in the SDC arm (190 [76.9%] men; 80 [32.4%] Black, 126 [51.0%] Hispanic or Latinx, 76 [30.8%] White, and 91 other race [36.8%]), were included in the intention-to-treat analyses. No statistically significant differences between arms were observed for the primary outcome (mean [SD] opioid-related adverse events, 3.29 [4.52] in the Relay arm and 4.10 [9.36] in the SDC arm; rate ratio, 1.02; 95% CI, 0.72-1.45; *P* = .90). By 12 months after enrollment, 24 participants (9.7%) had died (17 [70.8%] due to overdose). Relay participants reported high satisfaction with the ED intervention.

**Conclusions and Relevance:**

This randomized clinical trial examining the impact of an ED peer navigator intervention on subsequent opioid-related adverse events did not find significant outcome differences for Relay vs SDC participants. These findings highlight the importance of intervening to save lives in this high-risk population and suggest potential refinements to future ED peer intervention research.

**Trial Registration:**

ClinicalTrials.gov Identifier: NCT04317053

## Key Points

**Question:**

Does a peer navigator intervention delivered to patients presenting to an emergency department (ED) after a nonfatal opioid overdose reduce subsequent opioid-related adverse events?

**Findings:**

In this randomized clinical trial of 247 patients from 4 New York City EDs, the peer intervention arm and site-directed care arm demonstrated similar numbers of opioid-related adverse events in the 12 months after baseline.

**Meaning:**

These results suggest that future research should further examine impacts on other outcomes, such as mortality, and intervention modifications to enhance follow-up contacts and engagement with medications for opioid use disorder.

## Teaser

This randomized clinical trial assesses whether receipt of a peer navigator intervention following presentation to the emergency department (ED) with opioid-involved overdose is associated with reductions in opioid-related adverse events compared with site-directed care.

## Introduction

Overdose remains a leading cause of premature death in the US, and opioids continue to contribute to most overdose deaths.^1^ Emergency departments (EDs) are recognized as important sites for overdose prevention efforts.^2^ ED visits after nonfatal overdose are critical touchpoints; 1 in 20 ED patients presenting for nonfatal overdose dies of a subsequent overdose in the next year.^3^

An increasing body of research has examined ED-based interventions, including naloxone training and distribution, motivational interviewing, and buprenorphine initiation, and referral to treatment.^4,5,6,7^ Interventions involving trained peer workers with lived experience of substance use have been identified as potentially promising in EDs and other health care settings, given that peers may be able to better develop rapport and serve as trusted messengers vs other health care staff.^8,9,10^ However, there has been limited research on the effectiveness of such interventions.^11,12,13^

To address this gap, we conducted a randomized clinical trial (RCT) of a peer navigator intervention (Relay) delivered to ED patients presenting for care after nonfatal opioid overdose. Relay was launched in 2017 by the New York City (NYC) Health Department during a time of increasing overdose mortality rates and few ED-based standardized protocols to address overdose risk. Relay employs trained peer wellness advocates (WAs) who meet patients in the ED and continue to follow up with them by telephone or in person in the 90 days after discharge to offer tailored education, support, and referrals.^14^ We hypothesized that receipt of the Relay intervention would be associated with reductions in opioid-related adverse events (including fatal and nonfatal opioid-involved overdose as well as substance use–related ED visits) compared with site-directed care (SDC).

## Methods

### Study Design

A multisite, 2-arm (parallel) RCT using 1:1 permuted block randomization compared Relay with SDC, with enrollment from October 6, 2020, to June 30, 2022, and 12 months of outcome follow-up.^15^ Participants provided written informed consent, including consent to be randomly assigned to either the intervention (Relay) or SDC arm. Randomization used the REDCap randomization module, with a 1:1 ratio stratified by site and permuted blocks with variable block sizes. The intervention and study methods have been described in detail in past publications.^14,15^ The trial protocol can be found in Supplement 1. Additional study details appear in the eMethods in Supplement 2. The study was approved by the NYU School of Medicine institutional review board. The study follows the Consolidated Standards of Reporting Trials (CONSORT) reporting guidelines.^16^

### Setting and Participants

The trial was conducted at 4 NYC EDs representing 3 private, nonprofit health systems. Study EDs spanned 3 NYC boroughs and had participated in Relay for at least 1 year before study initiation.

ED workers (generally physicians) called the Relay hotline for patients presenting to a participating ED after a suspected nonfatal opioid-involved overdose. In the study EDs, calls to the hotline were routed to the study team and resulted in a research assistant being immediately dispatched to the ED to assess the patient for study eligibility, as has been previously detailed.^15^ Study eligibility criteria included age of 18 years or older, English- or Spanish-speaking, lived in NYC, medically stable (including not being diagnosed with COVID-19 at the ED visit and being alert and oriented), psychiatrically stable (not psychiatrically distressed and able to provide informed consent), not pregnant, not incarcerated or in police custody, not currently engaged in the Relay intervention, and opioid-involved overdose based on self-report^17^ or report of the treating ED worker. Opioid-involved overdose was defined as any overdose involving an opioid, whether intentional or unintentional (including overdose after using nonopioid drugs suspected to be contaminated with fentanyl).

### Site-Directed Care 

To ensure that all study participants received at least a minimum standard level of postoverdose care, research assistants offered SDC arm participants naloxone kits and a flyer on naloxone use and overdose response, a printed list of local addiction treatment programs, and an informational flyer about Relay so individuals could later self-refer if desired. Patients in both arms also received routine postoverdose ED care. In addition to overdose treatment and monitoring, patients could have received additional services, varying across EDs, such as fentanyl test strip distribution and brief intervention with referral to treatment.

### Intervention

Relay is operated by the NYC Health Department and delivered by trained peer navigators (WAs) (see eMethods in Supplement 2 and prior publications^14,15^). Relay serves ED patients presenting after a nonfatal opioid-involved overdose. ED workers at participating hospitals call a centralized Relay hotline to dispatch a WA to come to the ED (with an expected arrival time within 1 hour). The WA’s initial meeting with the patient focuses on building rapport and providing peer support, using a person-centered, harm reduction approach. In the ED, WAs provide individualized overdose risk reduction education, overdose rescue training and naloxone kits, tangible support (eg, a care bag with items such as snacks and socks), and referrals to additional services as needed. With their consent, patients are enrolled in Relay’s follow-up component, which offers 90 days of ongoing proactive support and connections to services. Although Relay was already operating in the study EDs, there was broad stakeholder agreement regarding the importance and ethical soundness of conducting an RCT, given the presence of clinical equipoise amid lack of evidence for comparable peer navigator interventions, and protections for the comparison arm described in the Site-Directed Care section. Patients were free to decline RCT participation, in which case they were still offered normal Relay services.

### Study Procedures and Data Sources

Research assistants administered a baseline questionnaire.^15^ Race and ethnicity were recorded based on self-report on the baseline questionnaire, using National Institutes of Health reporting categories. Race and ethnicity categories included Black, Latinx or Hispanic, White, and other race (including American Indian or Alaska Native, Native Hawaiian or Other Pacific Islander, Southeast Asian or Indian Subcontinent, other Asian, more than 1 race, other, or unknown). Data on race and ethnicity were collected because of large disparities in overdose observed in NYC and nationally due to structural racism. A WA was called to the ED to engage with Relay arm participants after completion of the baseline questionnaire. At the end of the visit, research assistants administered an exit survey documenting services received in the ED and participants’ thoughts about the intervention. Follow-up questionnaires were administered at 1, 3, and 6 months after baseline by telephone or, rarely, in person.

Administrative data were obtained from the 2 NYC Regional Health Information Organizations (RHIOs), Healthix and the Bronx RHIO, which together include data for visits to nearly all hospitals in NYC, aside from Veterans Affairs hospitals. RHIO data include dates and diagnostic codes for ED visits (administrative data further described in eMethods in Supplement 2). Mortality data were obtained from the NYC Health Department’s Office of Vital Statistics. The NYC Health Department provided Relay program data.

### Outcomes

The prespecified primary outcome was a composite count of opioid-related adverse events (defined as any opioid-involved overdoses [fatal or nonfatal] and any substance use–related ED visits) occurring in the 12 months after the baseline ED visit. All substance use–related ED visits were included in this composite outcome due to potential for misclassification bias given lack of sensitivity in ED visit diagnosis coding (ie, for overdose specifically).^18^ ED visits were classified as substance use–related if they included an *International Statistical Classification of Diseases and Related Health Problems, Tenth Revision (ICD-10) *diagnostic code for opioid, alcohol, or other drug use disorder, poisoning, or adverse effects, following definitions used in previous research (see eTable in Supplement 2 for the full code list).^19,20^ Fatal overdose and substance use–related ED visits were identified using administrative data sources. Nonfatal opioid-involved overdoses not resulting in ED visits were self-reported in follow-up questionnaires.

Prespecified secondary outcomes included self-reported initiation of medication for opioid use disorder (MOUD; buprenorphine, methadone, or naltrexone); self-reported overdose risk behaviors measured using the Overdose Risk Behavior score^17^; all-cause ED visits, ED visits for opioid overdose, and ED visits for other substance use reasons (all from RHIO data); self-reported opioid-involved overdose^17^; and self-reported time to next opioid-involved overdose. We additionally examined time to next substance use–related ED visit using RHIO data and patient satisfaction measures from baseline visit exit surveys. Exploratory mortality outcomes included deaths from all causes, overdose, and opioid-involved overdose within 12 months after enrollment. Outcome measurement details have been previously published.^15^

### Statistical Analysis

Multiple imputation using chained equations was used to address missing follow-up questionnaire data and to estimate nonfatal opioid-involved overdoses that did not result in ED visits during months 7 to 12, which were not covered by questionnaires. Missing data were imputed 100 times, using logistic regression for binary variables and predictive mean matching for all other variables. For outcomes that included self-report, imputed datasets were analyzed and results pooled using Rubin rules.^21^

Primary outcome analysis was conducted using a negative binomial regression model, adjusting for study site and substance use–related ED visits in the 90 days before baseline. For secondary and exploratory outcomes, we compared Relay and SDC arms using negative binomial regression for count variables, logistic regression for binary variables, Cox proportional hazards regression for time-to-event variables, and linear mixed-effects regression for Overdose Risk Behavior score. Study site and baseline assessment of the outcome were included as model covariates.

**Figure 1.**
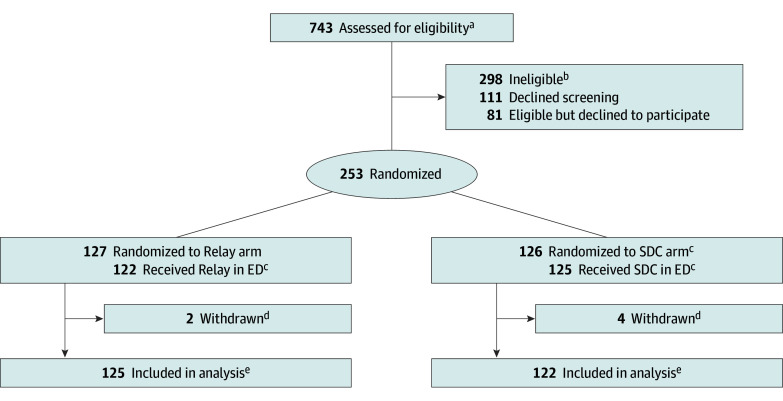
Recruitment, Randomization, and Participant Flow for Trial of Relay ED indicates emergency department; SDC, site-directed care. ^a^A total of 155 calls received from the Relay hotline did not result in eligibility assessment, most commonly due to study staff being unavailable or the patient leaving the ED before screening could occur. ^b^Reasons for ineligibility were too intoxicated to participate (n = 68); medically unstable, too ill to participate, or COVID-19 diagnosis (n = 57); already currently engaged in Relay (n = 45); did not have an opioid-involved overdose (n = 35); unable to provide informed consent (n = 29); did not live in New York City (n = 19); in prison or police custody (n = 18); psychologically distressed (n = 14); already enrolled in the study (n = 12); and pregnancy (n = 1). ^c^Five Relay arm participants did not engage with a wellness advocate (WA) at baseline (eg, because they left the ED before the WA arrived), although 1 enrolled in Relay the next day at a subsequent ED overdose visit. One SDC arm participant was enrolled in Relay at baseline due to staff error. Fifteen additional SDC arm participants enrolled in Relay after the baseline ED visit but within the study period (13 after a repeat overdose ED visit [generally for visits to nonstudy EDs, which by design did not screen patients for study participation] and 2 after calling Relay themselves and requesting to enroll). An additional 4 SDC arm participants interacted with a Relay WA at a subsequent ED visit at least once in the study follow-up period but did not enroll in the full Relay program for ongoing follow-up. All participants are included in their randomly assigned arm in the intention-to-treat analyses. ^d^Six participants were withdrawn (removed by the study investigators) because they were determined after randomization to have been ineligible. ^e^Follow-up survey questionnaires were completed by 128 participants (51.8%) at 1 month, 147 (59.5%) at 3 months, and 139 (56.3%) at 6 months. A total of 177 participants (71.7%) completed a follow-up questionnaire for at least one time point (86 [70.5%] in the SDC arm and 91 [72.8%] in the Relay arm). As described in the Methods, primary outcome assessment did not rely on the presence of self-reported data.

Participants were retained in their randomly assigned study arms following an intention-to-treat analytic approach (Figure 1). Analyses were conducted in the R statistical computing environment, version 4.5.1 (R Foundation for Statistical Computing).^22^

The original target sample size was 350 participants (with power calculations reported previously).^15^ However, enrollment was delayed by the COVID-19 pandemic, making this target unrealistic. We recalculated power and sample size during the study, which showed that a sample size of 240 participants would provide greater than 81% power to detect a rate ratio of 0.57 comparing Relay and SDC arms on the primary outcome. Power calculations were conducted using PASS software, version 21.0.2 (NCSS LLC).^23^ Two-sided *P* < .05 was considered statistically significant. Statistical analysis was performed from November 4, 2024, to May 6, 2025. Additional analysis details are provided in the eMethods in Supplement 2.

## Results

### Participant Characteristics

There were 247 patients enrolled in the trial, including 190 men (76.9%), 55 women (22.3%), and 2 people (0.8%) who were transgender, nonbinary, or other gender and 80 Black (32.4%), 126 Latinx or Hispanic (51.0%), 76 White (30.8%), and 91 other race (36.8%), including American Indian or Alaska Native, Native Hawaiian or Other Pacific Islander, Southeast Asian or Indian Subcontinent, other Asian, more than 1 race, other, or unknown. Table 1 gives detailed participant baseline characteristics by arm. A total of 253 patients were randomized to the Relay arm (127 participants) or SDC arm (126 participants); 6 were later withdrawn after being determined ineligible. The 247 remaining participants were included in the intention-to-treat analyses (125 in the Relay arm and 122 in the SDC arm) (Figure 1**)**. A total of 177 (71.7%) completed at least 1 follow-up questionnaire. Loss to follow-up was not significantly different between groups; details on questionnaire completion by arm and time point appear in the eMethods in Supplement 2.

**Table 1.** Participant Baseline Characteristics

Characteristic	No. (%) of participants	
	Relay arm (n = 125)	SDC arm (n = 122)
Enrollment ED		
A	61 (48.8)	60 (49.2)
B	22 (17.6)	22 (18.0)
C	21 (16.8)	21 (17.2)
D	21 (16.8)	19 (15.6)
Age group, y		
18-24	4 (3.2)	5 (4.1)
25-34	25 (20.0)	20 (16.4)
35-44	23 (18.4)	17 (13.9)
45-54	21 (16.8)	24 (19.7)
55-64	36 (28.8)	36 (29.5)
≥65	16 (12.8)	20 (16.4)
Gender		
Man	99 (79.2)	91 (74.6)
Woman	24 (19.2)	31 (25.4)
Transgender, nonbinary, or other	2 (1.6)	0 (0)
Language used for study procedures		
English	112 (89.6)	106 (86.9)
Spanish	13 (10.4)	16 (13.1)
Ethnicity		
Hispanic or Latinx	56 (44.8)	70 (57.4)
Not Hispanic or Latinx	69 (55.2)	52 (42.6)
Race^a^		
Black	37 (29.6)	43 (35.2)
White	42 (33.6)	34 (27.9)
Other^b^	46 (36.8)	45 (36.9)
Insurance		
Uninsured	5 (4.3)	12 (10.2)
Medicaid	80 (68.4)	77 (65.3)
Medicare or Dual (Medicaid and Medicare)	18 (15.4)	21 (17.8)
Private	3 (2.6)	3 (2.5)
Other or unsure	11 (9.4)	5 (4.2)
Missing	8 (6.4)	4 (3.3)
Educational level		
Less than high school diploma	37 (33.0)	47 (39.8)
High school graduate or GED	50 (44.6)	34 (28.8)
Some college or higher	25 (22.3)	37 (31.4)
Missing	13 (10.4)	4 (3.3)
Employment		
Working (full or part time)	17 (14.9)	19 (16.2)
Unemployed	60 (52.6)	51 (43.6)
Unable to work	25 (21.9)	31 (26.5)
Retired	12 (10.5)	16 (13.7)
Missing	11 (8.8)	5 (4.1)
Current housing situation		
Own apartment	38 (32.8)	38 (32.2)
Doubled up (eg, with friends or family)	26 (22.4)	25 (21.2)
Homeless shelter	19 (16.4)	23 (19.5)
Homeless, unsheltered	16 (13.8)	13 (11.0)
Other (eg, detox, halfway house, SRO, or hotel)	17 (14.7)	19 (16.1)
Missing	9 (7.2)	4 (3.3)
Jail or prison stay (lifetime)^c^	82 (71.9)	78 (68.4)
Substances used (past 3 mo)^c^		
Alcohol	69 (55.6)	63 (51.6)
Cannabis	61 (48.8)	66 (54.1)
Cocaine or crack	50 (40.0)	56 (45.9)
Hallucinogens	17 (13.6)	19 (15.6)
Heroin^d^	79 (63.2)	90 (74.4)
Methamphetamines	14 (11.2)	14 (11.5)
Prescription opioids	33 (26.4)	40 (33.3)
Prescription stimulants	12 (9.6)	11 (9.0)
Sedatives	32 (25.6)	28 (23.0)
Any polysubstance use (past 3 mo)^c^^,^^e^	88 (71.0)	89 (74.2)
Injection drug use (past 3 mo)^c^	34 (30.4)	21 (18.4)
MOUD use (past 3 mo)^f^	39 (33.3)	36 (30.5)
Overdose risk behavior score, mean (SD)	8.90 (8.13)	8.22 (6.35)

Abbreviations: ED, emergency department; GED, general educational development; MOUD, medication for opioid use disorder; SDC, site-directed care.

^a^ Race and ethnicity were self-reported separately by participants, with categories in accordance with National Institutes of Health reporting requirements.

^b^ The other category includes participants who answered American Indian or Alaska Native, Native Hawaiian or Other Pacific Islander, Southeast Asian or Indian Subcontinent, other Asian, more than one race, other, or unknown. Many participants who identified as Hispanic or Latinx chose the other category for the separate question on race.

^c^ There were small numbers of missing responses (not shown) for jail or prison stay (11 in Relay arm and 8 in SDC arm), polysubstance use (1 in Relay arm and 2 in SDC arm), injection drug use (13 in Relay arm and 8 in SDC arm), alcohol and hallucinogens (1 missing in Relay arm for each), heroin (9 in Relay arm and 7 in SDC arm), prescription opioids (1 in Relay arm and 4 in SDC arm), and sedatives (2 in Relay arm and 3 in SDC arm). Percentages are shown for the total denominator in each arm.

^d^ May have included fentanyl or other adulterants that had entered the heroin supply, of which participants may or may not have been aware.

^e^ Polysubstance use was defined as more than 1 category of substance used in past 3 months, not including tobacco or cannabis.

^f^ MOUDs include methadone, buprenorphine, or naltrexone taken for the purposes of treatment of opioid use disorder. A small number of responses were refused or missing (12 in Relay arm and 4 in SDC arm). Percentages are shown for the total denominator in each arm.

### Intervention Delivery

Of 125 participants randomized to the Relay arm, 121 (96.8%) interacted with a WA during their baseline ED visit or an ED visit the following day (1 participant); 110 (90.9%) baseline interactions occurred in person and 11 (9.1%) by telephone. Most participants interacting with a Relay WA at baseline agreed to receive ongoing WA follow-up contacts (98 [78.4%] of Relay arm participants). Of the remainder, 9 were not offered ongoing Relay engagement (because WAs did not believe they met program eligibility criteria), and 14 were offered enrollment but declined. Of 122 participants randomized to the SDC arm, 20 (16.4%) interacted with a Relay WA at least once during the study period (Figure 1).

### Outcomes

Relay arm participants reported high satisfaction with baseline visit interactions with WAs (Table 2) and gave high ratings for usefulness and comfort talking to a WA (Table 2). Approximately half of Relay arm participants had contact with a WA after baseline, most commonly by telephone (Table 2).

**Table 2.** Relay Arm Participant Experiences With WAs

Experience	No. (%) of Relay arm participants^a^ (n = 125)
Self-reported experiences (on participant baseline exit survey), mean (SD) (n = 95)^b^	
How helpful was it to talk with a Relay WA?	8.74 (2.23)
How satisfied were you with care you received from the Relay WA?	8.84 (2.17)
How comfortable did you feel talking to the Relay WA?	9.31 (1.52)
No. of referrals received by each Relay arm participant^c^	
0	88 (70.4)
1	18 (14.4)
2	8 (6.4)
3	9 (7.2)
4-5	2 (1.6)
Types of referrals made by WAs (at baseline or follow-up), No.^d^	
MOUD	11
Other SUD treatment	15
Overdose prevention centers and other harm reduction services	15
Medical care	7
Mental health care	11
Other services	42
No. of follow-up contacts with WAs^e^	
0	67 (53.6)
1	15 (12.0)
2	8 (6.4)
3	6 (4.8)
4	7 (5.6)
5	5 (4.0)
6-10	10 (8.0)
>10 (maximum, 17)	7 (5.6)
Follow-up contact modality^f^	
In person^g^	59 (21.8)
Telephone call	182 (67.2)
Text message	30 (11.1)

Abbreviations: MOUD, medication for opioid use disorder; SUD, substance use disorder; WA, wellness advocate.

^a^ Unless otherwise indicated.

^b^ Baseline exit survey was completed by 95 Relay arm participants. All self-reported experience questions were rated on a scale of 1 to 10, with 1 indicating not at all and 10 indicating very.

^c^ Includes referrals made by WAs for participants at the baseline emergency department visit or within the postbaseline engagement period to MOUD, other SUD treatment, overdose prevention centers or other harm reduction services, medical care, mental health care, and referrals to other services.

^d^ Individual referrals may include multiple service types, so the number of referrals by type may sum to greater than the total number of referrals. Referral outcomes (eg, completion) were not tracked.

^e^ Number of follow-up contacts in the postbaseline Relay engagement period among all 125 participants randomized to the Relay arm. Of the 98 participants who at the baseline visit enrolled or consented to receive follow-up Relay services, number of contacts in the next 90 days were 0 (42 [42.9%]), 1 (14 [14.3%]), 2 (8 [8.2%]), 3 (6 [6.1%]), 4 (7 [7.1%]), 5 (5 [5.1%]) 6 to 10 (10 [10.2%]), or more than 10 (6 [6.1%]). The postbaseline Relay engagement period was 90 days for all participants apart from 2 participants who had their engagement period extended to 120 days (allowed in select circumstances if requested by the WA).

^f^ Of 271 total follow-up contacts within 90 days of the baseline emergency department visit (among all Relay arm participants).

^g^ In-person visits include those made at Relay-participating emergency departments for participants who had subsequent overdose visits and other in-person encounters outside the emergency department.

There was no significant difference between the Relay and SDC arms on the primary outcome of opioid-related adverse events (rate ratio, 1.02; 95% CI, 0.72-1.45), a composite outcome of opioid-related fatal or nonfatal overdose and any substance use–related ED visits, or its individual components in intention-to-treat analyses (Table 3; eFigures 1-2 in Supplement 2). Relay vs SDC arm participants reported more overdose risk behaviors in the 6 months after baseline (mean difference in scores, 1.75; 95% CI, 0.42-3.08, with CI lower bounds above 0 indicating a significant difference [Table 3]). There were no statistically significant intervention effects for other secondary or exploratory outcomes.

**Table 3.** Outcomes for Relay vs Site-Directed Care Arm

Outcome	Relay (n = 125)	SDC (n = 122)	OR, RR, or HR (95% CI)^a^	*P* value
Opioid-related adverse events at 12 months, mean (SD)^b^	3.29 (4.52)	4.10 (9.36)	1.02 (0.72-1.45)	.90
Opioid overdose ED visits at 12 months, mean (SD)	0.30 (0.80)	0.29 (0.86)	1.08 (0.52-2.23)	.84
Other substance use–related ED visits at 12 months, mean (SD)	2.34 (4.07)	2.98 (8.72)	1.03 (0.66-1.60)	.90
Opioid-involved overdose by self-report at 6 months, No. (%)^c^	28 (30.8)	23 (26.7)	1.23 (0.66-2.29)	.52
Mortality at 12 months, No. (%)				
Opioid-involved overdose	5 (4.0)	9 (7.4)	0.52 (0.16-1.56)	.26
Overdose	7 (5.6)	10 (8.2)	0.66 (0.23-1.79)	.42
All cause	9 (7.2)	15 (12.3)	0.55 (0.22-1.30)	.18
ED visits for any diagnosis at 12 months, mean (SD)	6.28 (8.85)	6.73 (12.86)	1.09 (0.78-1.51)	.61
MOUD initiation within 90 days, No. (%)^d^	15 (17.4)	11 (12.8)	1.64 (0.68-3.94)	.27
Overdose Risk Behavior score, mean (SD)^e^	6.16 (5.09)	4.19 (4.09)	1.75 (0.42-3.08)^e^	.01
Time to next opioid-involved overdose (days after baseline)	NA^f^	NA^f^	1.25 (0.81-1.94)	.31
Time to next substance use–related ED visit	NA^g^	NA^g^	1.25 (0.90-1.74)	.18

Abbreviations: ED, emergency department; HR, hazard ratio; MOUD, medication for opioid use disorder; NA, not applicable; OR, odds ratio; RR, rate ratio; SDC, site-directed care.

^a^ RRs are reported for count variables, ORs for binary categorical variables, and HRs for time to event variables.

^b^ Primary study outcome: Any opioid-involved overdose (fatal or nonfatal) or any other substance use–related (including opioids and other drugs of abuse as well as alcohol) ED visit. This outcome was based on a combination of administrative data, self-report, and a prediction for the portion of the follow-up period not covered by self-report, as described in the Methods.

^c^ Among those with at least one follow-up questionnaire completed (91 in the Relay arm and 86 in the SDC arm).

^d^ MOUD initiation was examined only for participants not already taking MOUD at baseline (86 in the Relay and 86 SDC arm).

^e^ For Overdose Risk Behavior score, effect size is reported not as a ratio but as the mean difference in scores across groups (1.75), estimated from a linear mixed-effects regression model including covariates. The linear mixed-effects regression model compares Relay and SDC, incorporating all follow-up points in the analysis (1, 3, and 6 months). The CI lower bounds above zero indicates a significant difference (ie, more risk behaviors reported among the Relay group). This finding could indicate a true difference in behaviors, or it might signal that Relay participants had a better understanding of overdose risk or felt more comfortable talking openly about their risk behaviors.

^f^ See eFigure 5 in Supplement 2 for detailed results on time to next opioid-involved overdose.

^g^ See eFigure 6 in Supplement 2 for detailed results on time to next substance use-related ED visit.

**Figure 2.**
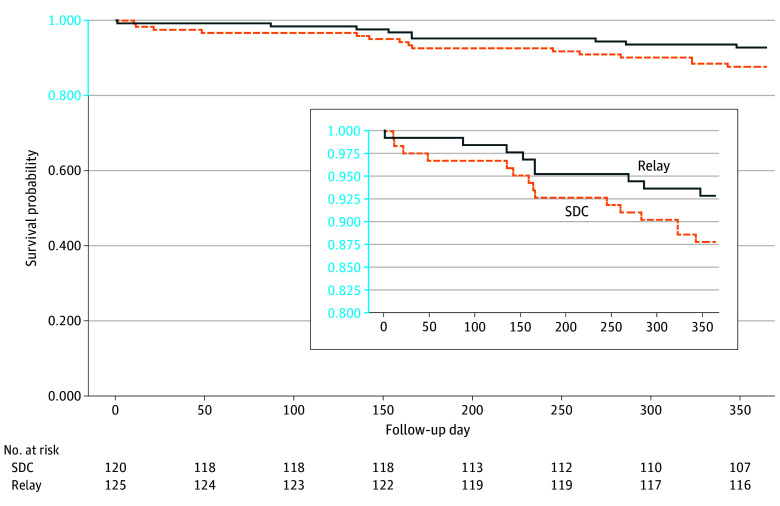
Survival Plot for All-Cause Mortality Comparing Relay and Site-Directed Care (SDC) Arm Participants

Overall, 24 study participants (9.7%) died within 12 months after baseline (7.2% in the Relay arm and 12.3% in the SDC arm). Survival plots (Figure 2; eFigures 3-4 in Supplement 2) did not indicate a mortality difference favoring Relay, as the null hypotheses of no arm difference could not be rejected. Most deaths (70.8%) were attributable to overdose.

## Discussion

The intent of this RCT was to compare a peer-delivered ED-based intervention that offered 90 days of follow-up vs enhanced SDC for patients presenting to an ED after a suspected nonfatal opioid-involved overdose. Importantly, the COVID-19 pandemic had a major impact on both the RCT and the intervention itself. In the 12 months after study enrollment, we did not find a significant difference between the Relay and SDC arms in subsequent opioid-related adverse events (defined as fatal or nonfatal opioid-involved overdose or any substance use–related ED visit). Study participants in both arms had strikingly high rates of all-cause mortality and overdose death. Our study highlights both the importance and the challenges of intervening to save lives in this high-risk population.

During the COVID-19 pandemic there were fewer calls from EDs to Relay, resulting in a lower than expected sample size. Pandemic-related disruptions in transportation for WAs responding to EDs and pressure by EDs to discharge patients quickly sometimes resulted in delayed or truncated ED engagements or, at times, initial engagements conducted by telephone. Moreover, during the pandemic post-ED Relay follow-up was largely conducted by telephone, hindering peers’ ability to build rapport and exacerbating challenges in reaching participants who lacked reliable telephone access—a common structural barrier particularly for the many participants who were homeless or unstably housed. In addition to these challenges, 23 Relay arm participants (18.4%) did not enroll in the follow-up component of Relay; thus, no outreach attempts were made. Overall, more than half of Relay arm participants had no post-ED follow-up contact with a WA, and, among participants with any follow-up, half had 3 or fewer contacts. The number of referrals made to services such as MOUD and other substance use disorder treatment was low, likely due to a combination of factors, including incomplete WA documentation and limited referral options during the pandemic and the program model’s emphasis on use of the baseline ED visit to develop rapport and meet immediate needs (with the goal that this would facilitate successful referrals at follow-up visits).

Low Relay follow-up visit rates observed in this RCT are consistent with those noted in a recently published study of Relay spanning all 14 EDs at which it operated from 2017 to 2022.^24^ As discussed in that study, patients who are “hardly reached” (a reframing of the individually focused language “hard to reach”) face structural barriers, including housing insecurity and other competing needs.^24,25^ Notably, the group of individuals with no follow-up visits had higher rates of opioid (vs nonopioid) drug use, injection drug use, and, ultimately, higher overdose mortality.^24^ Low rates of post-ED engagement highlight the critical role of the ED visit itself as an intervention site and the importance of programs such as Relay that engage with patients in the ED. Considering the RCT findings and ongoing program monitoring, Relay leadership has more recently engaged in quality improvement to bolster the services provided in the ED (eg, increasing referrals and distributing fentanyl and xylazine testing strips), improve outreach methods, and identify and address structural barriers to more effectively reach participants for follow-up.

We observed very high mortality rates for patients presenting to an ED after a nonfatal overdose, with 9.7% of study participants dying in the 12 months after baseline. Notably, during the study’s observation period, NYC had the highest rates of overdose deaths on record.^26^ Prior research in Massachusetts found that 1 in 20 ED patients presenting for overdose from 2011 to 2015 died in the next year^3^; the higher mortality rate observed in our study may have been driven by increased potency of the unregulated drug supply during the past decade.^27,28^ The role of EDs as critical touchpoints for people at high risk of overdose death was similarly underscored by a recent Canadian study showing that 70% of individuals who died of an opioid-involved overdose had visited an ED in the year before their death.^29^

Relay aims to reduce overdose fatality by providing patients with information and resources that will help them survive a future overdose. We observe a trend toward lower all-cause and opioid overdose mortality for Relay vs SDC arm participants; given small sample sizes, there is a high degree of uncertainty surrounding mortality impacts.^30^

Relay arm participants reported positive experiences with WAs in the ED. Qualitative research supports multiple beneficial roles of peers in engaging with individuals at risk for overdose.^8,9,31,32,33,34,35,36,37^ Qualitative research that our team conducted with ED workers, WAs, and ED patients highlighted how Relay filled gaps in ED services and was highly valued by ED workers, with participants noting the unique benefits of peer-delivered interventions.^33^ However, high-quality experimental research on peer-delivered interventions focused on opioid use disorder (OUD) or overdose prevention is still lacking.^7,38,39,40^ Our findings align with those of what is to our knowledge the only other published RCT to date of a peer-delivered ED overdose prevention intervention, which found that a peer-delivered intervention appeared equally effective vs a social worker–delivered intervention in connecting ED patients with substance use treatment and reducing subsequent nonfatal overdose.^11,12^ An observational study in New Jersey found that an ED peer recovery support service was associated with increased 60-day MOUD initiation and decreased subsequent overdoses but with significant heterogeneity across hospitals.^41^ Rigorous experimental research of peer-delivered interventions for patients with OUD in non-ED settings has shown mixed results.^19,42,43^

More research is needed to understand and optimize peer interventions for OUD, particularly in ED settings. One potential, yet understudied, role of peer workers is to assist with interventions designed to connect ED patients with MOUD, which currently demonstrate the most promising evidence in terms of ED interventions related to OUD and overdose.^6,40,44,45,46,47,48,49^ Finally, it is important to recognize that peer interventions for ED patients exist amid a broader environmental context marked by structural barriers, including lack of affordable housing, inadequate access to MOUD and harm reduction services, criminalization of drug use, and a volatile unregulated drug supply. Past research has highlighted the impact of these structural factors as they pertain to peer-delivered interventions and more broadly.^33,37,38,50^

### Limitations

This study has limitations. This was a pragmatic RCT conducted in EDs during the COVID-19 pandemic, which resulted in delays, changes in intervention delivery, and a lower sample size than initially planned. Power simulations indicated that the sample was adequate to show modest effects on the primary outcome but not for exploratory outcomes, including mortality. For example, considering mortality, the study had only 38% power to reject a null hypothesis of no mortality difference even when the true effect was to reduce the risk of mortality by half. As shown in eFigure 1 in Supplement 2, most values in the 95% CI for Relay’s effect on all-cause mortality show a benefit. More data are needed to allow more precise estimates and definitive conclusions about the impact of programs such as Relay on mortality, an outcome of clear public health significance. Additionally, the sample size limited our ability to examine potential impacts of Relay among subgroups of patients (eg, by individual characteristics or other services received during the ED visit) or to perform nuanced treatment effect and dose response analyses to determine whether individuals who received the Relay intervention per protocol and those with more Relay follow-up contacts had better outcomes.

For ethical reasons, research assistants provided SDC arm participants naloxone kits and a naloxone and overdose response educational handout, a printed list of local addiction treatment programs, and information on Relay; these services likely exceeded the standard of care in many EDs. Relay and SDC arm participants could also receive any intervention delivered as part of routine ED care, which, depending on the specific ED, may have included screening and brief intervention, referrals to services and outpatient substance use treatment, or, rarely, buprenorphine initiation. Services offered to the control group may have diminished the relative impact of Relay observed in this study.

We had an expected amount of loss to follow-up for postbaseline study questionnaires, resulting in missing data for self-reported outcomes. Because our primary outcome was largely based on administrative data, this is unlikely to have impacted primary outcome results but may have impacted some secondary outcomes. RHIO administrative data were missing a small number of ED visits, which was balanced across groups and appeared to be missing at random; thus, we do not anticipate impacts to our findings related to the effectiveness of Relay, but the count data in this article likely underestimate actual visit numbers. In addition, outcome measurement did not include hospitals outside NYC or nonhospital visits.

Additionally, our RCT examined only a subset of quantitative patient-level outcomes and should not be interpreted as demonstrating lack of value of ED peer navigator interventions after overdose. We did not examine potentially important impacts on ED workers (eg, satisfaction and reduced stigma) or on peers themselves (eg, employment and income). Qualitative research conducted by our team demonstrated a range of beneficial impacts of Relay on ED workers, patients, and WAs.^33^

## Conclusions

In this multisite RCT conducted during the COVID-19 pandemic, we did not find statistically significant differences in next-year opioid-related adverse events favoring the Relay peer navigator intervention vs SDC for ED patients presenting after a nonfatal opioid-involved overdose. More research is needed to clarify the impact of peer interventions on mortality and other key outcomes and to further optimize the design and delivery of peer-led ED interventions such as Relay.

## Notes

Supplement 1.
Trial Protocol

Supplement 2. **eMethods.** Additional Details on Relay Intervention and Statistical Analyses **eTable.** List of Diagnosis Codes for Substance Use-Related ED Visits **eFigure 1.** Summary of Outcome Effects for Relay vs. Site-Directed Care Arms (Intent-to- Treat Analyses) **eFigure 2.** Visualization of Primary Outcome (Opioid-Related Adverse Events) for Relay and SDC Arms (Intent-to-Treat Analyses) **eFigure 3.** Survival Plot for Overdose Mortality Comparing Relay and SDC Arm Participants (Intention-to-Treat Analysis) **eFigure 4.** Survival Plot for Opioid Overdose Mortality Comparing Relay and SDC Arm Participants (Intent-to-Treat Analysis) **eFigure 5.** Survival Plot for Time to Next Opioid-Involved Overdose Comparing Relay and SDC Arm Participants (Intent-to-Treat Analysis) **eFigure 6.** Survival Plot for Time to Next Substance Use-related ED Visit Comparing Relay and SDC Arm Participants (Intent-to-Treat Analysis)

Supplement 3.
Data Sharing Statement
