# Supplementary material for: Peer Navigator Intervention and Opioid-Related Adverse Events for Emergency Department Patients: A Randomized Clinical Trial
Source: JAMA Netw Open. 2026 Feb 6;9(2):e2555903. doi: 10.1001/jamanetworkopen.2025.55903 (PMC12881982; doi:10.1001/jamanetworkopen.2025.55903)
Supplement: Supplement 1. — Trial Protocol [file jamanetwopen-e2555903-s001.pdf]

Multi-Center Randomized Controlled Trial of Relay- NYC's Nonfatal Overdose Response Program

Initial version: **0**1/08/2020
Amended versions: 10/01/2020**;** 10/09/2020; 01/11/2021; 05/25/2021; 06/21/2021; 07/19/2021; 12/22/2022

Statement of Compliance

This study will be conducted in accordance with the Code of Federal Regulations on the Protection of Human Subjects (45 CFR Part 46), any other applicable US government research regulations, and institutional research policies and procedures. The Principal Investigator will assure that no deviation from, or changes to the protocol will take place without prior agreement from the sponsor and documented approval from the Institutional Review Board (IRB), except where necessary to eliminate an immediate hazard(s) to the trial participants. All personnel involved in the conduct of this study have completed Human Subjects Protection Training.

List of Abbreviations

AE	Adverse Event/Adverse Experience
BADUPCT	Bureau of Alcohol and Drug Use Prevention, Care and Treatment
CDW	NYC Health and Hospitals (H+H) Clinical Data Warehouse
CFR	Code of Federal Regulations
CRF	Case Report Form
CSOC	Clinical Study Oversight Committee
DCC	Data Coordinating Center
DHHS	Department of Health and Human Services
DOHMH	New York City Department of Health and Mental Hygiene
DSMB	Data and Safety Monitoring Board
ED	Emergency Department
EHR	Electronic health record
FFR	Federal Financial Report
FWA	Federalwide Assurance
GCP	Good Clinical Practice
HIPAA	Health Insurance Portability and Accountability Act
ICF	Informed Consent Form
IRB	Institutional Review Board
ISM	Independent Safety Monitor
MOP	Manual of Procedures
MOUD	Medication for opioid use disorder
N	Number (typically refers to participants)
NIH	National Institutes of Health
OEND	Overdose education and naloxone distribution
OD	Overdose
OHRP	Office for Human Research Protections
OHSR	Office of Human Subjects Research
OUD	Opioid use disorder
PI	Principal Investigator
PM	Project Manager
QA	Quality Assurance
QC	Quality Control
RA	Research Assistant (may also refer to Project Manager)
RCT	Randomized Controlled Trial
RHIO	Regional health information organization

SAE	Serious Adverse Event/Serious Adverse Experience
SDC	Site-directed Care
SOP	Site-specific Operating Procedure
US	United States

Protocol Summary

Title	Multi-Center Randomized Controlled Trial of Relay- NYC's Nonfatal Overdose Response Program
Short Title	Relay RCT
Brief Summary	The New York City (NYC) Department of Health and Mental Hygiene (DOHMH) has implemented Relay, a novel program that engages and intervenes with individuals in the ED following an opioid OD and for the next 90 days, with the goal of preventing subsequent OD events. The proposed randomized controlled trial will evaluate the impact of Relay on preventing subsequent opioid-related adverse events. A total of 350 eligible individuals with nonfatal opioid OD presenting to one of four participating EDs will be enrolled and randomized to one of two arms: 1) site-directed care (SDC) or 2) Relay—peer-delivered OD education and treatment linkage, including 90 days of peer navigation. Outcomes will be measured for 12 months through interviews and administrative health data.
Phase	Phase 3
Objectives	Aim 1: Evaluate the effectiveness of Relay to reduce the frequency of opioid-related adverse events (any opioid-involved OD [fatal or nonfatal] or any other substance use-related ED visit), in the 12 months following the index visit, the primary outcome.<br><br>Aim 2: Evaluate the impact of Relay on the following secondary outcomes: initiation of medication for opioid use disorder (MOUD); OD risk behaviors; self-reported opioid-involved OD; time to next opioid-involved OD; and frequency of ED visits for any cause, opioid OD, and other substance use related reasons.<br><br>Aim 3: Identify mediators (e.g., OD knowledge, support, experience of stigma, peer modeling) and moderators (e.g., substance use history, age, sex, housing status) of the effect of Relay.<br><br>Aim 4: Evaluate Relay's implementation by assessing fidelity to the program model and exploring barriers and facilitators to its delivery and sustainability through qualitative interviews with patients, Relay peer navigators, and ED providers.
Methodology	Randomized Controlled Trial of a behavioral intervention
Endpoint	Primary endpoints: opioid-related adverse events (OD deaths, nonfatal OD events, and other substance use related ED visits)<br>Secondary endpoints: initiation of medication for opioid use disorder (MOUD); OD risk behaviors; self-reported opioid-involved OD; time to next opioid-involved OD; and frequency of ED visits for any cause, opioid OD, and other substance use related reasons.
Study Duration	The estimated time from when the study opens to enrollment until completion of data analysis is approximately 33 months.
Participant Duration	6 months active participation

Duration of behavioral intervention	Total duration of intervention: 90 days<br>The behavioral intervention is comprised of two components, both delivered by the peer navigator. The first takes place in the ED, where the peer distributes naloxone and delivers tailored OD education, treatment referrals and support. The second component begins after discharge and consists of up to 90 days of peer navigation to provide ongoing support and linkage to services including MOUD, harm reduction programs, and social services.
Population	Adults (≥18 years), English- or Spanish-speaking, presenting to a participating ED with nonfatal opioid OD
Study Sites	Study sites are three health systems, representing four high-volume NYC hospital EDs. The sites are geographically dispersed and serve patient populations that are diverse with respect to age, sex, race, and ethnicity.<br><br>1. NYU Langone Health – Tisch Hospital & NYU Langone Hospital—Brooklyn<br>2. Mount Sinai Beth Israel<br>3. St. Barnabas Hospital
Number of participants	350 participants expected to be enrolled in the RCT across 3 health system EDs (4 EDs total).<br><br>Approximately 10-12 ED provider participants, approximately 10 Relay peer navigator participants, and approximately 12 patients will complete qualitative interviews.
Description of Study Intervention/Procedure	Relay is a novel program that engages and intervenes with individuals in the ED following an opioid OD and for the next 90 days, with the goal of preventing subsequent OD events. Relay is delivered by trained peer navigators, who are DOHMH staff with lived substance use experience. Relay navigators provide counseling, linkage to services, and OD prevention education.
Reference Therapy	Site-directed care (SDC): site-specific existing interventions delivered to patients with OUD as part of ED care. At a minimum SDC arm patients will receive OD education and naloxone distribution (OEND), a list of opioid treatment programs, and an informational flyer about Relay.
Key Procedures	Survey administration, education and counseling (for Relay arm), qualitative interviews
Statistical Analysis	The primary analysis will use a Poisson generalized linear regression model to evaluate the intervention effect on the total number of opioid-involved OD and substance use-related ED visits. We anticipate the availability of 12 months of follow-up data on all participants, but in the event of death or withdrawal from the study, we will adjust the follow-up time appropriately using an offset term. We will include adjustment for study site, as well as other baseline factors that may be unbalanced across arms. The primary effect measure will be the incidence rate ratio comparing Relay to SDC. Some participants may be routinely high utilizers of the ED, and this may lead to overdispersion of the count outcome; we will use a negative binomial model instead of a Poisson model if warranted. Statistical analysis for other aims are described in the full protocol.

Schematic of Study Design

Figure 3. Study design

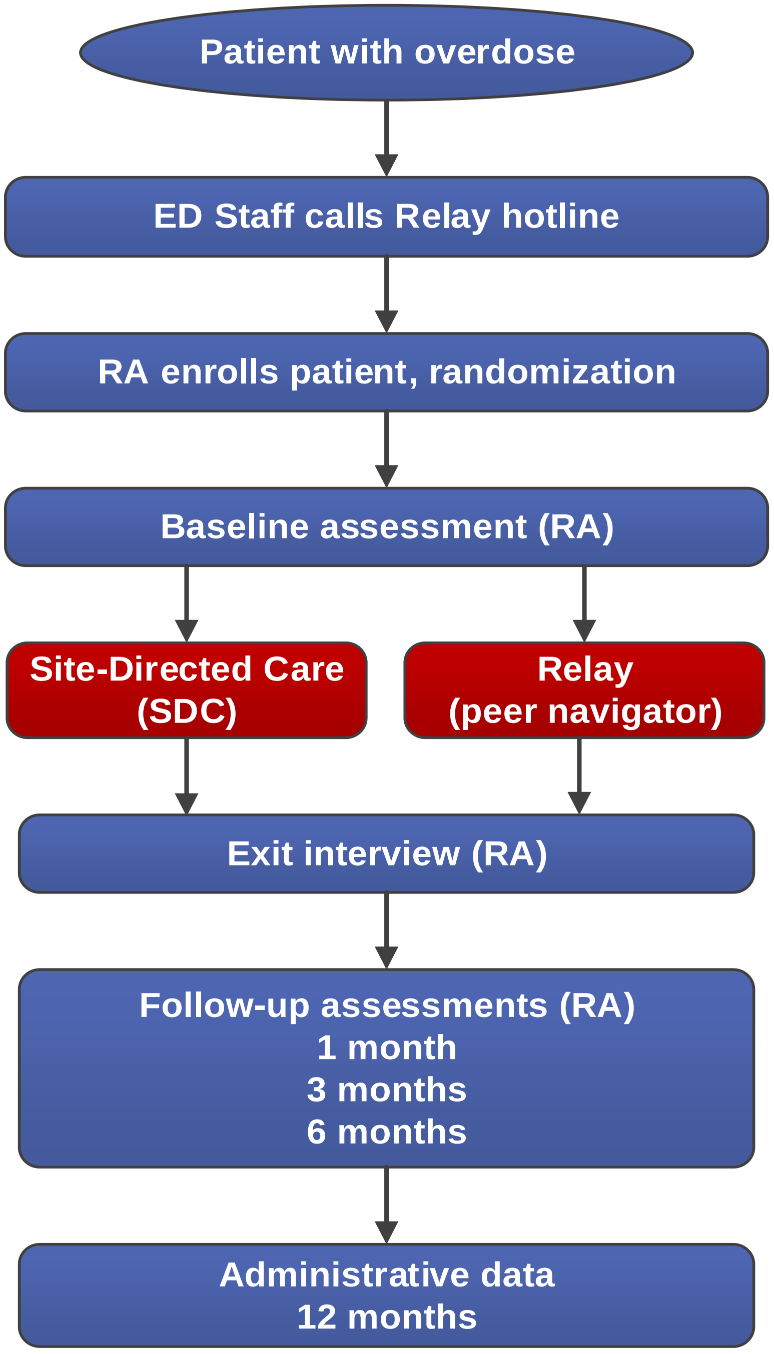

This material is the property of the NYU School of Medicine and NYU Langone Health. Do not disclose or use except as authorized in writing by the study sponsor

1 Key Roles

Principal Investigators (for NYULH study site and overall study)
Kelly Doran, MD, MHS
NYU Langone Health
Assistant Professor, Departments of Emergency Medicine and Population Health
462 1st Avenue, Room A-345
New York, NY 10016
212-263-5850
Kelly.doran@nyulangone.org

Jennifer McNeely, MD, MS (MPI)
Department of Population Health
180 Madison Ave., 17th floor
New York, NY 10016
646-501-3551
Jennifer.mcneely@nyulangone.org

2 Introduction, Background Information and Scientific Rationale

2.1 Background Information and Relevant Literature

The increasing rates of overdose (OD) deaths and prevalence of opioid use disorders (OUD) are urgent medical and public health issues in the US.[1-3] The number of OD deaths has risen annually for the past decade.[4,5] In 2016, the US saw over 63,000 drug OD deaths, an increase of 28% from the previous year.[6] Two-thirds of these OD deaths involved opioids. Deaths are increasingly driven by heroin and synthetic opioids,[2] and deaths involving fentanyl specifically have increased exponentially in recent years.[7]

As the opioid crisis worsens, the number of emergency department (ED) visits for opioid overdose is rising, to over 140,000 in 2017 (a 30% increase from the prior year).[8] In the US, the northeast had the highest rate of opioid OD ED visits in 2017, with over 20 OD visits for every 10,000 ED visits for any cause.[8] The largest increases in, and highest rates of, opioid-related ED visits occurred in large metropolitan areas.[8] Mirroring national trends, *from 2015-2016, New York City (NYC) saw its largest single-year increase in the number of drug OD deaths*, with 483 more drug overdose deaths in 2016 than in 2015.[9] In 2017, there were 1,487 unintentional drug OD deaths in NYC, a rate of 21.2 per 100,000 New Yorkers.[9] Opioids were involved in 82% of these drug OD deaths.[9] The increase in OD deaths is driven in large part by the penetration of fentanyl into the drug supply. In each year prior to 2015, fentanyl was present in fewer than 4% of OD deaths.[10] In contrast, fentanyl was present in 16% of OD deaths in 2015, increasing to 44% in 2016. In 2017, 57% of 1,487 total OD deaths involved fentanyl.[11]

Public health authorities are looking for solutions. In March 2017, the NYC Mayor's Office launched the HealingNYC initiative, which committed $38 million each year to programs intended to reduce opioid OD deaths.[12] In March 2018, the NYC Mayor's Office announced an additional $22 million investment into HealingNYC to expand existing initiatives and launch new strategies, bringing the total HealingNYC investment to $60 million.[13] This initiative leverages the resources of the city's Department of Health and Mental Hygiene (DOHMH) to prevent opioid OD and increase access to opioid treatment. HealingNYC has spurred the rapid development and launch of innovative programs to address the opioid crisis, but does not provide resources for rigorous evaluation of their effectiveness.

ED visits for nonfatal opioid overdose are a missed opportunity for intervention. Individuals with prior nonfatal OD are at greatly increased risk of having another OD[14-16] and are 2-3 times more likely to subsequently die of OD,[17-20] in comparison to individuals who use drugs and have not previously overdosed. A statewide study in Massachusetts found that 10% of those who had a nonfatal initial OD reversed by naloxone died within one year, many from opioid OD.[21] Following a nonfatal overdose—whether or not naloxone is given—the most likely care setting for an individual is the ED.[22,23] This high-risk population has few other contacts with health care providers,[24] and the ED visit for nonfatal opioid OD thus represents a rare opportunity to reach these patients and engage them in care.[25] While national

This material is the property of the NYU School of Medicine and NYU Langone Health. Do not disclose or use except as authorized in writing by the study sponsor

leaders in addiction and emergency medicine have called for a more coordinated and robust response in EDs following opioid OD, current practice in the majority of EDs nationwide and in NYC is to discharge patients after a brief observation period, without providing OEND or effectively linking patients to opioid treatment programs.[25-33] Some EDs do offer treatment referrals, but referrals alone are rarely effective; it takes considerable effort to engage individuals in care.[34-38]

The scale and urgency of the opioid crisis have led to the rapid development and implementation of novel strategies aimed at preventing overdose fatalities. Public health authorities are developing and deploying new, theory-based programs even in the absence of rigorous scientific evidence supporting their effectiveness. A cornerstone of DOHMH's response to the opioid crisis is Relay, a novel program that uses peer navigators to reach New Yorkers following an OD.

A large literature highlights the positive impact of peer navigation interventions for a range of public health problems and conditions, though they have only recently been deployed to address OD. Navigation approaches were originally developed to reduce racial/ethnic disparities in cancer outcomes, and peer navigation approaches have been found effective across a range of populations and cancer types.[39,40] Similarly, a large body of evidence supports the peer navigation approach for improving HIV treatment outcomes. A peer navigation approach may be particularly well suited to patient populations that are highly stigmatized and mistrustful of health care professionals, such as those addressed in the proposed study.[41] Peer navigators, by drawing on their lived experiences, can serve to reduce stigma.[42] Further, peer navigators are positioned to serve as effective positive role models.[42] These factors (social modeling, reduced stigma) are critical elements of social-cognitive behavior change models. Thus for stigmatized, wary, and distrustful populations, peer navigation interventions show high levels of acceptability, and this promotes feasibility, engagement, motivation to change behavior, and intervention effectiveness.

Grounded in a theoretical model that integrates the theory of triadic influence[43] and dynamic social impact theory,[44] and incorporating a motivational interviewing counseling approach, Relay is a behavioral intervention that is carried out by trained DOHMH peer navigators who have lived experience with substance use. These peer navigators are deployed to collaborating EDs when the ED provider caring for an individual presenting after a nonfatal OD calls the Relay hotline (the hotline is a component of the DOHMH Relay program). The Relay intervention is designed to overcome the specific barriers to secondary prevention of subsequent OD experienced by these patients in the ED, in the immediate aftermath of the OD, and after discharge. The intervention is comprised of two well-integrated components, both delivered by the peer navigator. The first takes place in the ED, where the peer navigator engages individuals in patient-centered OD education, provides a naloxone kit if one was not given by ED staff (family/friends with the patient are also given kits), and facilitates initiation of treatment for OUD through motivational counseling and referrals. The second component begins after discharge and consists of up to 90 days of peer navigation to provide ongoing support and linkage to services including MOUD, harm reduction programs, and social services. Theoretically, in the ED and post-ED, peer navigators serve as positive role models while providing tangible and instrumental support and reducing stigma, which fosters motivation and capacity to change risk behaviors and initiate treatment, thereby reducing risk for future OD.

Experts have called for EDs—open '24/7/365' and uniquely accessible—to do more to address the opioid crisis.[45] Some academic health systems have adopted peer 'recovery coaches' in the ED, whose primary role is to link patients to treatment. These programs are propagating in response to the opioid crisis in advance of strong evidence of their effectiveness.[29,46,47] To date, there has been only one small retrospective study of the effectiveness of an ED-based peer opioid OD response program.[48] This study examined The Rhode Island Lifespan Opioid Overdose Prevention (LOOP) program—which is similar to Relay in using peers to engage and support patients after an OD event in the ED and for 90 days following the event—compared to OEND and usual care. Non-significant trends were observed that generally favored both the 'recovery coach' and OEND groups over usual care, though MOUD initiation rates appeared lower in the recovery coach group.[48] The study authors suggested future randomized controlled trials to more rigorously examine the effectiveness of similar peer navigator programs.[48]

Overall, trials of other ED-based OD prevention interventions have been limited by small sample sizes and heterogeneous populations and interventions, which constrain the applicability of their results to real-

CONFIDENTIAL
This material is the property of the NYU School of Medicine and NYU Langone Health. Do not disclose or use except as authorized in writing by the study sponsor

world practice. A study by Bohnert et al.[49] identified 204 ED patients at risk for prescription opioid OD in EDs and provided a single-contact motivational interviewing intervention. Participants reported significant reductions in OD risk behaviors, but the study did not examine repeat OD events. A study by Banta-Green et al.[50] screened ED patients for risk of opioid OD, and tested a single-contact brief intervention in high-risk users of illicit and prescription opioids. This study found no significant difference in OD rates between groups (~20% experienced an OD within 12 months), but it was limited by low enrollment and loss to follow-up. There is also increasing interest in ED-initiated buprenorphine and referral to ongoing treatment. Past studies have shown promising, albeit mixed, results; for example, a study by D'Onofrio et al. showed that such an intervention resulted in increased connection to outpatient treatment and lower illicit opioid use at 2 months but not at 6 or 12 months.[38,51] Interventions focused on ED-based buprenorphine initiation tend not to address many of the potent barriers to treatment among vulnerable ED patients—including competing subsistence needs, lack of social support, and stigma—which peer navigator programs attempt to address.

In summary, while ED-initiated peer navigator approaches appear to be well accepted and theoretically promising in reducing secondary opioid OD, rigorous study is urgently needed to determine their effectiveness. Because research has thus far not demonstrated a benefit of peer navigator approaches over other approaches to ED patients presenting after opioid OD (such as OEND alone), clinical equipoise exists to justify a randomized controlled trial. The proposed RCT promises to fill a critical research gap, providing important information about evidence-based responses to prevent OD among the population at highest risk.

2.2 Rationale

The feasibility of Relay is already established, but its effectiveness has not yet been studied. DOHMH is interested in studying Relay's effectiveness to inform critical decisions around whether the program should be modified, replaced, or further expanded. DOHMH is committed to optimizing NYC's response to its continuing opioid OD crisis, including by evaluating existing programs to ensure that limited resources are being used effectively. Because programmatic funding for Relay does not support more than basic process evaluation, DOHMH has partnered with NYU researchers to develop this proposal to study Relay's impact on preventing opioid-related adverse events (subsequent fatal or nonfatal OD, and substance use-related ED visits).

This study seeks to add a rigorous evaluation component to the Relay program initiative, by introducing random assignment to Relay versus site-directed care (SDC) at five EDs. Our evaluation uses structured interviews, administrative data, and qualitative interviews with patients and other stakeholders to characterize Relay's implementation, effectiveness, and potential for future dissemination and sustainability.

By engaging individuals at high risk for OD at a reachable moment and providing a sustained peer-delivered intervention, the Relay approach has potential to reduce subsequent OD and opioid-related adverse events. In the midst of an opioid crisis, health authorities are developing programs aimed at reducing OD deaths, even in the absence of strong evidence. This is a critically important time to understand which of these new approaches are effective, and under what conditions, so that they can be brought to scale to improve population health. The Relay program is feasible, well accepted by EDs and patients, and holds promise as an OD prevention intervention that could be scaled up in NYC and nationally, but first its effectiveness must be established. The proposed randomized controlled trial will provide the quality of evidence that is required to determine whether peer interventions like Relay can impact the opioid crisis by reducing future opioid-related adverse events for patients who have presented to the ED for nonfatal opioid OD, above and beyond other interventions already being implemented in EDs in the wake of the opioid crisis.

2.3 Potential Risks & Benefits

2.3.1 Known Potential Risks

This study poses no more than minimal risk to participants. The main risk of participation is loss of confidentiality. Personally identifiable information and personal health information collected for the study

will be kept to the minimum required, though the study does involve collection of participant identifying information that is needed to allow examination of health care administrative data for the 12 month outcome. Participant contact information will also be collected for purposes of reaching participants for completion of study visits. Participant responses to questionnaires include sensitive questions such as questions about substance use. Risk of loss of participant confidentiality or participation information will be guarded against by the measures described in subsequent sections of this protocol. Additionally, this CDC-funded study will be covered by a Certificate of Confidentiality, as it meets the criteria specified by the 21st Century Cares Act.

Patients. There is a possibility that patients may feel uncomfortable while completing some study assessments. We will minimize discomfort by having interviews conducted by a trained and qualified RA, who will take steps to maximize participant privacy even in a busy ED setting (e.g., asking visitors to leave, moving the patient to a more private area). Patients will be informed that all aspects of their participation are voluntary and that they may choose not to complete assessments or to discontinue their participation entirely, without repercussion or penalty. Participants in the Relay arm may feel uncomfortable while discussing sensitive topics with Relay peers. The Relay peers are highly experienced in discussing sensitive topics with participants, and participants may choose to end uncomfortable conversations at any time. In the informed consent process, participants will be told that if they become psychologically distressed during the study procedures they can request to speak to someone about their feelings (e.g., a physician). If a participant voices suicidal ideation during study procedures, RAs will follow procedures that include immediate reporting, as will be detailed in the study SOP/MOP and included in RA training materials.

ED staff and Relay peers (qualitative interviews). ED staff are employees of the three study hospital system sites (NYULH, Mt. Sinai Beth Israel, St. Barnabas Hospital). Relay peers are employees of DOHMH. Adequate protections will be implemented to ensure that participation in the qualitative interviews does not jeopardize their employment or professional standing. Specifically, all audio recordings will be transcribed at the earliest opportunity and any personally identifying information will be removed from the transcripts. All analyses will utilize only the transcripts from which personally identifying information is removed. Audio recordings and transcripts will not be shared with supervisors of ED staff or Relay peers; only aggregate information will be shared with study team members who may hold supervisory roles over participants (DOHMH staff and ED site leads), such that individuals cannot be identified. No information about personal substance use will be solicited in the interviews.

2.3.2 Known Potential Benefits

Participants are not expected to benefit from the study directly, as they will not be receiving new interventions not otherwise available to them in the ED. We expect that overdose education and provision of naloxone (in both conditions) will reduce future opioid-related adverse events among patients presenting with nonfatal OD; of note, OEND is generally standard care at study sites already, but for the study we will ensure it occurs for all study participants. The Relay peer navigator intervention has the potential to provide additional benefit to patients, but its effectiveness has not been established. The community at large, including patients and providers, stands to benefit from the knowledge gained from this study about reducing OD fatalities, and barriers and facilitators to OD prevention programs. The results of this study are critical to advancing science around preventing OD fatalities, informing the allocation of resources in NYC, and informing other jurisdictions as they scale up opioid OD prevention efforts.

3 Objectives and Purpose

3.1 Primary Objective

Aim 1: To evaluate the effectiveness of Relay to reduce the frequency of opioid-related adverse events (any opioid-involved OD [fatal or nonfatal] or any other substance use-related ED visit), in the 12 months following the index visit, the primary outcome.

CONFIDENTIAL
This material is the property of the NYU School of Medicine and NYU Langone Health. Do not disclose or use except as authorized in writing by the study sponsor

3.2 Secondary Objectives

Aim 2: To evaluate the impact of Relay on the following secondary outcomes: initiation of medication for opioid use disorder (MOUD); OD risk behaviors; time to next opioid-involved OD; ED visits for opioid OD, other substance use reason, and any cause; and self-reported opioid-involved OD.

Aim 3: To identify mediators (e.g., OD knowledge, support, experience of stigma, peer modeling) and moderators (e.g., substance use history, age, sex, housing status) of the effect of Relay.

Aim 4: To evaluate Relay's implementation by assessing fidelity to the program model and exploring barriers and facilitators to its delivery and sustainability through qualitative interviews with patients, Relay peer navigators, and ED providers.

4 Study Design and Endpoints

4.1 Description of Study Design

This 2-arm multi-center RCT compares site-directed care (SDC) to the Relay intervention among patients presenting to the ED with a nonfatal opioid-involved OD. When individuals present to the ED with opioid OD, ED staff call the Relay hotline. The hotline operator will notify the research team. A RA will meet the patient in the ED, assess study eligibility, complete informed consent, and randomize participants to the SDC vs. Relay arm. Participants in both arms will complete interviews with research staff at baseline and at 1, 3, and 6 months after enrollment, and will provide consent for viewing their information in administrative data sets. Administrative data provide measures for the primary 12-month outcome analysis. All appropriate and applicable permissions will be obtained from the holders of administrative data prior to their use for research. Qualitative interviews will provide additional information on implementation of Relay.

4.2 Study Endpoints

4.2.1 Primary Study Endpoints

The primary endpoint is the number of opioid-related adverse events in the 12 months following the index ED visit. Opioid-related adverse events include: any opioid-involved OD (fatal or nonfatal) or any other substance use-related ED visit.

4.2.2 Secondary Study Endpoints

Secondary endpoints include the impact of Relay on the following outcomes:

- Initiation of medication for opioid use disorder (MOUD)
- OD risk behaviors
- Time to next opioid-involved OD
- ED visits for any cause
- ED visits for opioid OD
- ED visits for other substance use reasons
- Self-reported opioid-involved OD

We expect that Relay will be effective in increasing MOUD initiation because it is built on a foundation of prior work demonstrating that ED interventions by non-medical staff can effectively link patients to treatment. Measures of *MOUD initiation* will be gathered at each study visit. MOUD is defined as use of methadone, buprenorphine, or naltrexone that is prescribed by an office-based provider or provided by a treatment program as treatment for OUD. Initiation is defined as receiving MOUD within the 90 days following the index visit, for individuals who received no MOUD for at least 2 weeks prior to the index

This material is the property of the NYU School of Medicine and NYU Langone Health. Do not disclose or use except as authorized in writing by the study sponsor

visit.[38] Though infrequent, if patients are initiated on MOUD during the index visit (in the ED or, if admitted from the ED, in the hospital), the first outpatient MOUD encounter post-discharge will be the measure of treatment initiation. We will include MOUD initiated in the ED in sensitivity analyses. Our primary analysis is limited to the individual's first episode of outpatient MOUD in the 90 days (3 months) following enrollment because this is the duration of contact with the Relay peer navigator and thus, for individuals in the Relay arm, is most attributable to the intervention. However, we will measure the total number of MOUD initiation episodes and time receiving MOUD for a full 6 months, to capture any continued intervention effect. Assessments at baseline and follow-up will collect data on MOUD in the past 90 days.

Relay offers motivational counseling as well as sustained peer navigation that provides support and access to services that could help individuals reduce their OD risk behaviors. As such, we expect to detect greater reductions in risk behaviors in the Relay compared to the SDC arm. We will measure _OD risk behavior_ using questionnaires that were developed by a group of OD researchers based on knowledge of OD behavioral risk factors and used in 3 prior studies to capture OD risk behavior.[49,50,52] These questions measure the frequency, in the past 3 months, at which individuals: 1) used >1 opioid; 2) used benzodiazepines, alcohol, or cocaine/stimulant within 2 hours of an opioid; 3) used alone or behind a locked door; 4) used in a place they had never used before; and 5) used more than usual amount. The total risk score is an aggregation of responses to the individual questions.

Time to next OD is measured using self-report. _Frequency of ED visits for any cause_ (including visits that are not substance use-related) will be measured to assess whether there are differences in overall acute care use representing potentially negative health events. Additionally, we will examine frequency of _ED visits for opioid OD_ and frequency of _ED visits for other (non-OD) substance use reasons_. These three ED visit secondary endpoints will be count outcomes using administrative data at 12 months and based on documentation of visit diagnosis (including primary and secondary diagnosis ICD codes, text diagnosis, and/or chief complaint). _Self-reported opioid-involved OD_ is based on participant self-report in the 1-, 3- and 6-month follow-up visits and includes nonfatal ODs that did or did not result in an ED visit.

We will also examine multiple potential mediators and moderators of Relay program effectiveness as further described in later sections.

4.2.3 Exploratory Endpoints

Exploratory endpoints include the impact of Relay on the following outcomes:

- Health services contacts (medical, mental health, harm reduction program)
- Possession of naloxone; number of times naloxone used
- Naloxone carrying by members of social network
- Death from any cause
- Opioid-involved OD deaths
- Any OD deaths

Relay peer navigators are trained to take a motivational and patient-centered approach to reducing OD risk, which includes working with clients to reduce opioid use-related harms even if they are not ready to enter treatment. Death outcomes will be tracked using Vital Statistics records and DOHMH OD death reports (accessed by DOHMH) since prior research shows high rates of death, including those unrelated to OD, among patients following ED visits for non-fatal opioid OD.[48] Other exploratory outcomes will be ascertained by participant self-report at baseline and follow-up visits.

5 Study Enrollment and Withdrawal

5.1 Inclusion Criteria

5.1.1 Patients

Participants in this study are patients presenting to a participating ED with non-fatal opioid OD. Because we seek to enroll a broad and representative sample of individuals experiencing nonfatal opioid OD,

This material is the property of the NYU School of Medicine and NYU Langone Health. Do not disclose or use except as authorized in writing by the study sponsor

exclusion criteria are limited. To be eligible to participate in this study, an individual must meet all of the following criteria:

1. Adults (≥18 years)
2. English- or Spanish-speaking
3. Patient at a participating ED presenting with nonfatal opioid OD. Nonfatal opioid OD is initially determined by the treating ED clinician(s) based on the patient's clinical characteristics, history, and/or response to naloxone treatment and is operationalized as the treating ED clinician calling the Relay hotline. Patients and/or ED providers will confirm opioid-involved OD at the time of eligibility assessment.
4. Currently residing in NYC

5.1.2 **ED Staff and Relay Peers** (Qualitative Interviews)

For ED staff interviews, purposeful sampling will be used to select individuals with a variety of roles who have experience with the Relay program (e.g., physician, social worker). For Relay peers, purposeful sampling will be used to select peers with a variety of experiences (e.g., different amounts of time with the program, both part-time and full-time employees). Specific eligibility criteria include:

1. Adults (≥18 years)
2. English speaking
3. ED staff with current involvement with the Relay program or Relay peers
4. Currently employed in the ED at a participating study site or by the Relay program as a peer navigator

5.2 Exclusion Criteria

5.2.1 Patients

An individual who meets any of the following criteria will be excluded from participation in this study:

1. Unable to provide informed consent
2. Currently incarcerated, currently living in another controlled environment prohibiting research contacts, or currently in police custody
3. Known to be currently pregnant, based on patient or provider report
4. Already participating in the study (patients will only be enrolled into the study once)
5. Already actively engaged in the Relay program (enrolled within the past 90 days)
6. Speaks only Spanish, at a site and time period when no Spanish-speaking RA is available.

Individuals admitted to the hospital from the ED are eligible to participate in the study.

5.2.2 ED Staff and Relay Peers (Qualitative Interviews)

1. Unable to provide informed consent

5.3 Vulnerable Subjects

This study will not enroll vulnerable subjects, which under federal regulation include children, pregnant women, fetuses, neonates, and prisoners. Children under the age of 18 will be excluded. Pregnant women are excluded from the study because they have specific needs and access to services that are not generalizable to other populations. Women will be asked if they are currently pregnant as part of the study screening process; no further testing or questioning about pregnancy during the study will be done.

Prisoners, as defined by the federal Office for Human Research Protections (OHRP), will not be enrolled in the study. Under these definitions, "prisoner" means "any individual involuntarily confined or detained in a penal institution. The term is intended to encompass individuals sentenced to such an institution under a criminal or civil statute, individuals detained in other facilities by virtue of statutes or commitment

procedures which provide alternatives to criminal prosecution or incarceration in a penal institution, and individuals detained pending arraignment, trial, or sentencing (45 CFR 46.303(c)).” Pertinent to the current study, “individuals who are detained in a residential facility for court-ordered substance abuse treatment as a form of sentencing or alternative to incarceration” as well as “parolees who are detained in a treatment center as a condition of parole” are defined as “prisoners.” However, in accordance with federal definitions, “individuals who are receiving non-residential court-ordered substance abuse treatment and are residing in the community” as well as “persons living in the community and sentenced to community-supervised monitoring, including parolees” are *not* considered prisoners. Patients who are not currently incarcerated, under arrest, or otherwise detained but who may be on probation or parole will not be excluded from the study as these patients are not considered prisoners for research.

5.3.1 Inclusion of Patients who become Prisoners during the Study

It is possible that during the course of the study, some individuals in our study population may become considered prisoners. Our study enrolls a population with high rates of criminal justice involvement as a consequence of their opioid use. Incarceration is a major risk factor for opioid-related overdose, and data on overdose and other substance-related adverse among this population is important for evaluating the effectiveness of the Relay intervention in our population of interest.

Though the study will not enroll people who are considered prisoners at the time of enrollment, participants could potentially become prisoners over the course of their participation in the study. We will seek OHRP approval to ascertain follow-up data using administrative data sources only from these participants during the periods in which they are considered prisoners. We will not attempt to complete follow-up surveys with prisoners who are currently detained in a residential facility, due to a lack of ability to contact them in a secure, HIPAA-compliant manner. We do plan to use hospital administrative data that may include dates during which these individuals are considered prisoners. This data will be gathered through data abstraction from a variety of administrative health databases, as described in section 14.1. As collection of this information does not involve directly contacting the participants, we do not expect that the use of this administrative data constitutes an increased risk to these individuals.

We will not be notified when a study participant becomes considered a prisoner during the course of the study. We do include questions regarding whether a person has been incarcerated since the previous study visit in our follow-up surveys. For follow-up study visits that are conducted by phone, research staff will confirm that the participant has privacy and is not a prisoner prior to administering research assessments.

If an individual is considered a prisoner past the end of the study, we will not conduct any additional follow-up, but will use the hospital administrative data from the time that they were a study participant. If an individual is considered a prisoner at one point in the study, and later in the study is no longer considered a prisoner, we will attempt to conduct all follow-up assessments as scheduled.

We do not expect any direct benefit to individuals who are considered prisoners over the course of the study. We do expect that the information gathered about these individuals will provide important information for the study and may benefit our population of interest in the longer term.

We do not expect that the collection of administrative data and select follow-up information from this population poses more than minimal risk. We will take steps to protect this data as outlined in section 13.5.

5.4 Strategies for Recruitment and Retention

5.4.1 Patients

We plan to enroll 350 participants. Patients arriving to the emergency department (ED) with a nonfatal opioid overdose (OD) for which the ED providers have called the Relay hotline will be approached by

This material is the property of the NYU School of Medicine and NYU Langone Health. Do not disclose or use except as authorized in writing by the study sponsor

trained RAs, after they are medically stable. RAs will be alerted to potentially eligible patients by the Relay Hotline, which is activated by the ED care providers or staff when a patient presents to the ED with a nonfatal opioid OD. RAs will request permission from the patient to inform them about the study, assess eligibility, explain the study procedures and incentive schedule, and offer enrollment to those who are eligible. We estimate that we will be able to meet recruitment goals over an approximately 15-month period.

Inclusion of women and minorities. Patients will not be excluded on the basis of sex/gender, race or ethnicity. The sample is expected to be representative of the patient population presenting after opioid OD to the five diverse urban EDs chosen as study sites. Based on NYC Department of Health and Mental Hygiene Syndromic Surveillance data for opioid OD presentations at the study site EDs, women are anticipated to represent 33% of the study sample and the proportion of racial/ethnic minorities included in the sample will vary by site, and will range from 27% to 83%. To ensure the ability of diverse patients to participate in the study, study materials will be available in both English and Spanish.

Retention Plan. Successful tracking and retention over time is a multifaceted effort requiring simultaneous strategies at the management, staff, participant, and compensation levels. To enhance retention in follow-up procedures, we will use several methods that have yielded long-term retention rates above 80% in numerous prior studies with vulnerable populations.[53-55] These methods include the following:

1. **Locator form and contact information**. We will ask participants to provide detailed locator information at the time of enrollment. The locator information form will include participants' phone numbers, addresses, email addresses, and at least three secondary contacts (e.g., friends, case workers) that we may use if we are unable to reach them directly. Collecting this information is necessary to improve ability to contact study participants for follow-up visits, given that participants' own contact information may change during the course of the study. Further, locator form information will include information on places frequently visited by participants (e.g., methadone treatment programs, homeless shelters). We will collect information on participants' social media "handles" for Facebook, Instagram, etc. so that we can also locate them via these methods if necessary. Of note, when using secondary contacts or social media accounts, research staff members will only say they are calling/reaching out from the "emergency department study," "NYU health study," or "health study" and will not divulge the nature of the research study. Participants will be informed they should only provide contact information they are comfortable with. Collecting such detailed locator information in addition to phone numbers is necessary given that some participants may not have a stable phone number of their own, and mobile phone numbers may change. Locator information will be confirmed and updated at each contact and at each follow-up assessment.

2. **Tracking**. Retention efforts will begin shortly after enrollment. We will maintain regular contact via mail (e.g., a thank you card sent after the baseline interview, holiday cards). These are useful ways to maintain rapport and returned mail will help identify participants at risk of becoming lost to follow-up. All recruitment and retention materials will be approved by the IRB. Materials sent by mail do not mention substance use but instead reference a "emergency department study," "NYU health study," or "health study."

Tracking participants begins with a direct approach: contacting the participant by phone, email, social media, and/or mail. If direct tracking is not successful, we will begin more intensive outreach efforts. The first step will be to contact participants' secondary contacts (e.g., friends, family, case workers) listed on the locator form (for whom participants have given our consent to contact). If these tracking methods are unsuccessful, we will initiate systems-level tracking. At enrollment, participants will have been asked to list agencies with which they have contact, and we will send a letter or flyer to each of these agencies requesting that they forward the enclosed letter to the participant's most current address. Additionally, we will use free systems-level tracking methods commonly used to locate participants in longitudinal studies. These include searching web-based phone and address directories, as well as criminal justice directories. In some cases we will attempt place-based tracking, canvassing locations where the participant reports

often spending time. Further, we will monitor ED visits to study hospitals made by patients subsequent to the baseline visit, which may present an opportunity for performing follow-up visits. This monitoring will occur via Epic track boards and alerts provided to the study team via a secure and password protected online portal by the HealthIx RHIO (see Section 14.1).

3. **Compensation**. We will reimburse participants for their time completing study activities, as described in more detail in the sections that follow.

4. **Reminders**. Beginning approximately two weeks before each follow-up assessment is due, study staff will begin reaching out to participants. Staff will make multiple attempts at different times of day and days of the week, and via different contact modalities, to reach participants. Staff will conduct multiple contact attempts for participants who do not complete scheduled assessments; windows before and after the follow-up assessment due date will be allowed to complete the assessments (up to 1 month will be allowed for the final 6-month assessment), though attempts will be made to complete them as close as possible (+/- 3 days) to the due date.

5. **Flexibility**. Follow-up visits may be completed by telephone and may occur during evening or weekend hours. This will make it easier for participants to schedule and complete their assessments, without requiring them to travel to a research site.

5.4.2 ED Staff and Relay Peers (Qualitative Interviews)

We plan to enroll approximately 22 participants (10-12 ED staff and 10 Relay peer navigators) for qualitative interviews. ED staff who have current involvement with the Relay program and Relay peers will be contacted by study staff to inform them about the study, assess eligibility, explain the study procedures and incentives, and offer enrollment to those who are eligible. As described in Section 5.1.2, for ED staff interviews, purposeful sampling will be used to select potential participants who have a variety of roles who have experience with the Relay program (e.g., physician, social worker,), to maximize the amount we are able to learn about the program's implementation. For Relay peers, purposeful sampling will be used to select peer participants with a variety of experiences (e.g., different amounts of time with the program, both part-time and full-time employees). As participation in the qualitative interviewing portion of the study is limited to one interview, retention of participants is not applicable.

5.4.3 Use of DataCore/Epic Information for Study Procedures

Study staff will use Epic to look up information about patients after receipt of a call about a potentially eligible patient from the Relay hotline (as described in the protocol), to gather information about potential study eligibility and likely duration of stay in the ED. Study staff will also monitor the Epic ED track boards for NYU Langone—Tisch and NYU Langone Hospital—Brooklyn to look for study participants presenting to the EDs subsequent to the baseline visit, which may present an opportunity to perform a follow-up visit. As described elsewhere in the protocol, Epic will also be used to complete the baseline visit exit survey and collect outcome data.

5.5 Duration of Study Participation

The duration of active study participation is approximately 6 months. After screening and enrollment in the ED, participants in both arms will complete interviews with research staff at baseline and at 1, 3, and 6 months and will provide consent for viewing their information in administrative data sets for 12 months.

5.6 Total Number of Participants and Sites

Recruitment will end when approximately 350 evaluable participants are enrolled across all three study sites (representing 4 EDs). It is expected that approximately 355 total participants will be enrolled in order to produce 350 evaluable participants. If there are multiple participants who withdraw consent to use their data after they are enrolled or otherwise need to be terminated from the study, we may recruit additional

participants as needed to evaluate the primary study endpoints. There are no specified target accrual numbers for NYU Langone vs. other study sites, as enrollment will occur based on ED providers having patients who present for opioid OD for whom they activate the Relay hotline; therefore, the researchers do not have control over at which site potentially eligible patients will present.

We will also enroll approximately 10-12 ED provider participants and 10 Relay peer navigator participants in qualitative interviews to learn about barriers and facilitators to the intervention model; these are just estimates and the exact number of participants in the qualitative interviews will be guided by theoretical saturation as is standard best methodologic practice. Qualitative interviews are described in Section 7.3.5.

5.7 Participant Withdrawal or Termination

5.7.1 Reasons for Withdrawal or Termination

Participants are free to withdraw from participation in the study at any time upon request.

An investigator may choose to terminate a participant's participation in the study if:

- Any clinical adverse event (AE), laboratory abnormality, or other medical condition or situation occurs such that continued participation in the study would not be in the best interest of the participant.
- A participant is discovered to have been enrolled in the study despite not having met study exclusion criteria <u>at the time of enrollment</u> (e.g., dementia / unable to understand study consent).

Of note, while pregnancy and incarceration are exclusion criteria for enrollment in the study, if a participant becomes pregnant or a prisoner <u>during the course of the study</u>, they will not be terminated from the study. Administrative data will still be used for assessment of outcomes, and study interviews may be conducted with pregnant participants (See Section 5.3.1 for details on prisoner assessments).

5.7.2 Handling of Participant Withdrawals or Termination

If participants request to withdraw from the study, they will be asked whether the data already collected from them can still be used. Participants will be asked to provide their request for study withdrawal in writing, sent to the study PI. Participants may choose not to complete individual follow-up interviews without actually formally withdrawing from the study; in such cases investigators will affirm with participants that we can still access their health care data for measurement of the study outcomes. The primary outcome measures use administrative data, which will be available for all individuals who enroll in the study, even if they are lost to follow-up, unless they have withdrawn permission for us to use their data. Participants who withdraw or are terminated from the study may still receive continued services from the DOHMH Relay program if they desire them.

If a participant is terminated from the study by the investigators, the participant will be informed in writing via a letter mailed to their primary address. Due to confidentiality concerns, details of the reason for termination will only be provided to the participant directly by phone.

5.7.3 Premature Termination or Suspension of Study

This study may be temporarily suspended or prematurely terminated if there is sufficient reasonable cause for such suspension or termination. Written notification, documenting the reason for study suspension or termination, will be provided by the suspending or terminating party to Kelly Doran, Jennifer McNeely, and the CDC. If the study is prematurely terminated or suspended, the PI will promptly inform the IRB and will provide the reason(s) for the termination or suspension.

Circumstances that may warrant termination or suspension include, but are not limited to:

- Determination of unexpected, significant, or unacceptable risk to participants related to the study.
- Insufficient compliance to protocol requirements.
- Meeting the study stopping rule described in Section 8.7.

The study may resume once concerns about safety and protocol compliance are addressed and satisfy the sponsor and/or IRB.

6 Behavioral/Social Intervention

6.1 Study Behavioral or Social Intervention(s) Description

Individuals assigned to the Relay arm will receive the Relay peer navigator-led intervention. The Relay program is designed and administrated by DOHMH; the NYULH study investigators do not have control over Relay program operations. DOHMH may modify the intervention components or administration. The Relay program model by design is tailored to patient needs and therefore exact intervention components delivered to individual Relay participants may differ; the study investigators will monitor but will not attempt to change intervention components delivered. The present study is adding 1) random assignment and 2) evaluation procedures to investigate the implementation and effectiveness of Relay.

DOHMH's Relay program comprises two components, both conducted by the peer navigator.

The first component is conducted in the ED after the patient has been medically stabilized and consists of 1) engagement and support, to build relationships and foster trust; 2) tailored overdose prevention information and risk reduction counseling; 3) training on naloxone and provision of a naloxone kit if one has not yet been provided by the ED (or additional kits if needed); and 4) offer to follow up with the individual for up to 90 days to provide ongoing support and linkage to services, including opioid treatment programs. Throughout this component, the peer navigator serves as a positive role model and seeks to reduce stigma by highlighting shared lived experiences. Patients may be uncomfortable, distressed, and anxious to be discharged; peer navigators address these concerns in a number of ways. Consistent with the motivational interviewing approach, peer navigators support patients' autonomy and 'roll with resistance.' Engagement is fostered through tangible support (obtaining snacks, getting answers to questions) and instrumental (social) support. Tailored OD prevention education and training on naloxone is provided when patients are comfortable and engaged, and takes a motivational interviewing approach. The peer navigator obtains the patient's contact information and makes plans to follow up with them.

The second component comprises peer navigator follow-up in-person and by phone for up to 90 days. The goal of these contacts is to provide ongoing support and linkage to treatment, harm reduction, health care, and social services. Similar to the first intervention component, the peer navigator serves as a role model and shares his/her personal experiences as a means of reducing stigma and fostering engagement and motivation. Follow-up activities may vary depending on the needs of the participant but could include: ensuring that the participant has a naloxone kit and fostering discussion of how naloxone can be used to protect the participant and those in his/her social network from a fatal OD (e.g., provide kits to social network members, discuss naloxone with drug use partners); using a problem-solving approach to highlight and help resolve factors that might impede uptake of referrals, such as transportation challenges, lack of clarity on where to obtain services, and stigma; asking participants to clarify their personal goals regarding service use and risk reduction. In general, in these contacts, the peer navigator 'checks in' with the participant without applying pressure or judgment, monitors steps taken by the participant to achieve personal goals, and provides encouragement and support. Taken together, these activities in the ED and post-discharge are designed to foster *motivation* to engage in services, carry naloxone, initiate MOUD, and reduce risk for future OD; provide *support* for steps taken toward these outcomes; and foster *access* to services and resources.

Site-directed Care (SDC) arm. Patients randomized to the SDC arm will receive site-specific care delivered to patients with OUD/SUD. All of the site EDs included in this study have implemented a variety of their own initiatives—apart from Relay—for patients who present with OUD or after opioid OD. These initiatives include, but are not limited to: becoming credentialed as OOPP's (opioid overdose prevention programs), employing health educators to distribute naloxone kits, implementing "hard stops" in the electronic medical record to prevent patient discharge prior to naloxone receipt, conducting universal screening for drug and alcohol use, providing SBIRT (screening, brief intervention, and referral to

treatment) services, and ED-initiated buprenorphine (for X-waivered ED physician prescribers) with outpatient follow-up. The exact services / initiatives vary by site and are provided based on site-specific protocols. Study investigators will not attempt to influence SDC but will track the OD prevention-related ED care that participants receive with a patient exit survey administered during the baseline study assessments. The exit survey will document care delivered by ED staff including OEND, buprenorphine induction, substance use screening, counseling/brief intervention, and treatment referrals. Collecting this data will allow us to consider individual differences in ED services received in our analyses.

In the SDC arm, RAs will ensure that—at a minimum—patients receive overdose education and naloxone distribution (OEND) as well as a list of opioid treatment programs. RAs will give patients DOHMH OD education materials (including general information on OD and specific instructions on how to use naloxone) and 1-2 DOHMH-supplied naloxone kits (if one has not already been given by the ED staff). RAs will also give SDC arm patients a printed list of information on addiction treatment programs.

Patients in the SDC arm will also receive a flyer with information about Relay and a telephone number they can call to receive Relay services at any time after their ED visit. While we expect few patients will call for services, DOHMH and the study investigators agree that for fairness all study participants should be offered the ability to receive Relay services if they choose to do so. DOHMH and research staff will track any Relay services provided in this patient-initiated manner. After the 12 month study follow-up period (for the primary outcome analysis) has concluded, DOHMH will contact participants who had been assigned to the site-directed care arm to offer Relay services.

All patients (in the study or otherwise) can receive the full range of services offered at the individual study ED per existing protocols at each site.

6.1.1 Administration of Intervention

The Relay intervention consists of two components, both administered by the peer navigator. The first component is delivered in-person in the ED after the patient has been medically stabilized. The peer navigator then obtains the patient’s contact information and makes plans to follow up over the next 90 days. Follow-up visits are provided in person or by phone with a Relay peer navigator. The exact services, mode, timing, frequency, and duration may depend on individual participant needs and desire to participate. The intervention is administered by DOHMH. The NYU study investigators are not administering the intervention.

6.1.2 Procedures for Training Interventionalists and Monitoring Intervention Fidelity

Relay peer navigators receive thorough training and ongoing supervision from DOHMH. DOHMH may change training protocols without approval from the study investigators. The study investigators are not responsible for training Relay peer navigators. Currently, a standardized approximately 3-day new hire orientation and training covers all facets of the Relay program including OD education, naloxone distribution, MOUD, treatment referrals, tenets of peer navigation, building relationships, assessing and addressing patient needs, and documentation of patient contacts. Peer navigators additionally receive approximately 24 hours of formal training in motivational interviewing. Peer navigators meet with supervisors (Licensed Masters-Level Social Workers or Mental Health Counselors) regularly and participate in monthly group supervision meetings for ongoing training, program updates, and presentation of difficult cases. Supervisors also provide assistance and individual support for difficult cases. Additionally, peer navigators receive training in self-care and participate in voluntary group sessions with an external social worker to discuss any frustrations, stress, feelings, and emotions regarding their Relay work.

Study staff: Study RAs will complete privacy and security, HIPAA, and human subjects protections trainings. RAs will be employees of NYU School of Medicine as well as the study sites (for baseline visits at those site EDs). All RAs will receive standardized, thorough training in the study procedures prior to beginning work on the study. All RAs will receive ongoing supervision from the study Project Manager under direction from the PIs and Site PIs.

Fidelity monitoring of intervention: We will assess fidelity as follows: 1) All participants will complete an RA-administered exit survey interview at the conclusion of the baseline visit (in some cases the exit survey may be completed by phone the next day). The exit survey will assess what interventions the participant received in the ED that are relevant to opioid OD (e.g. substance use screening, OD education, naloxone kit(s) received, treatment referral/follow-up offered). The exit survey interview will also ensure that SDC arm participants receive, at a minimum, OEND, a list of treatment programs, and a handout with Relay program information. RAs will need to check off boxes in the exit survey indicating that the participant has received these items. Participants will also be asked at follow-up assessments what additional Relay program services they have received in the interval since the last assessment, 2) After each participant contact, Relay peer navigators complete content checklists to capture the duration of the interaction and activities performed during the session. To minimize burden to DOHMH and the peer navigators, the study will use the existing peer navigator contact content checklists used by DOHMH. Throughout the study, we will use this data to assess intervention dose, measured using the number, type (phone or in-person conversation, accompaniment to appointments), and duration of contacts; this information is routinely logged by the Relay peer navigator for DOHMH, as part of regular program activities. We will obtain a MOU/DUA to use these checklists to assess study fidelity.

6.1.3 Assessment of Subject Compliance with Study Intervention

Consistent with the motivational interviewing approach, participants who do not comply with the study intervention (e.g., decline referrals or decline to set substance use risk reduction goals) remain in the Relay intervention, and the peer navigator continues to offer support. Participant receipt of intervention components will be tracked both through self-report assessments and through Relay program data.

7 Study Procedures and Schedule

7.1 Study Procedures/Evaluations

Study procedures/evaluations in this protocol include: screening, consent, enrollment, randomization, research interviews (self-reported assessments and semi-structured qualitative interviews), overdose prevention education, as well as data extraction from administrative data sets and Relay programmatic data. All participants receive standard medical care in the ED. Those in the Relay intervention arm additionally receive the DOHMH Relay program, and those in the SDC arm additionally receive RA-delivered OD prevention education. There are no laboratory results, biospecimens, or images collected as part of this research protocol.

7.1.1 Study Specific Procedures Overall

- Eligibility assessment and study consent
- Randomization to study arm
  - Relay intervention: peer navigator administered OEND, motivational counseling, referrals and support and linkage to services while in the ED and for up to 90 days post-discharge.
  - Site-directed Care: RA will ensure that participant receives, at a minimum, overdose prevention education consisting of OEND, treatment program list, and Relay informational handout.
- Administration of questionnaires for patient-reported outcomes, at baseline, 1-month, 3-months, and 6-months.
- Baseline exit survey documenting opioid-related services received by the participant in the ED.
- Collection of participant information (to allow linkage with administrative data)
- Collection of participant locator form information
- Data extraction from administrative datasets including vital statistics / mortality data, DOHMH OD deaths data, and health services use data (which may include Regional Health Information System [RHIO], Statewide Planning and Research Cooperative System [SPARCS], Medicaid, and electronic medical record [EMR] data, as available)
- Semi-structured qualitative interviews with Relay participants, peer navigators, and ED staff.

This material is the property of the NYU School of Medicine and NYU Langone Health. Do not disclose or use except as authorized in writing by the study sponsor

- Assessment of study intervention adherence (Relay contact content checklists and assessment of DOHMH Relay program data)

7.1.2 Standard of Care Study Procedures

All patients in the study (Relay arm + SDC are) will receive standard medical care as they would regularly receive in the ED, including a range of relevant substance use services offered at the individual study ED per existing care protocols at each site. These services vary by site and are offered to patients either by clinician discretion or according to specific protocols at each study site. Relevant services vary by site and by patient and may include, but are not limited to: health education, distribution of naloxone kits, universal screening for drug and alcohol use, SBIRT (screening, brief intervention, and referral to treatment), and ED-initiated buprenorphine with outpatient follow-up. Patients also receive standard medical care as they would usually receive in the ED. The exact services vary depending on site- and patient-specific factors and will be captured using patient exit surveys. The study investigators will not attempt to change standard care provided at study sites.

7.1.3 ED Staff and Relay Peer Qualitative Interview Procedures

We will enroll approximately 10-12 ED provider participants and 10 Relay peer navigator participants in qualitative interviews to learn about barriers and facilitators to the intervention model; Interviews will be conducted one-on-one with an interviewer trained in qualitative research techniques. To allow flexibility given the ongoing COVID-19 pandemic, interviews will be conducted either in-person, by phone, or by secure web conferencing (e.g., WebEx). At the qualitative interview contact the interviewer will review the verbal consent script, attain verbal consent for the study and for audio recording, and complete a one-on-one in-depth interview in which the interviewer uses an interview guide. Interviews will be semi-structured, guided by an interview guide (see Interview Guide Attachment), with flexibility to allow for additional questions as needed and to maximize interview flow. Interviews will be digitally recorded and professionally transcribed. As noted previously, any identifying information will be removed from transcripts prior to analysis. Basic characteristics of interview participants (e.g., age, race/ethnicity, gender, job role) will also be recorded, and the interviewer will record field notes at the conclusion of the interview with any additional pertinent observations. Qualitative interview participants will receive an incentive payment at the conclusion of the interview.

7.2 Laboratory Procedures/Evaluations

Not applicable.

7.3 Study Schedule

7.3.1 Screening and Enrollment/Baseline

Screening/Enrollment/Baseline Visit (Visit 1, Day 0)

There is no separate screening visit for this study. Screening for study eligibility will occur during the same ED or hospital visit as the study enrollment / baseline visit. The only patients who are potentially eligible for the study and who will be screened by RAs are those for whom the ED staff has called the Relay program hotline. As part of existing protocols, ED staff at the study sites call the Relay program hotline for patients who have presented to the ED after a nonfatal opioid OD. ED staff are instructed to call the Relay hotline when a patient presents with nonfatal opioid OD, as determined by the treating clinician(s). The Relay hotline is operated by a HIPAA-compliant call center that provides 24-hour service, 365 days/year. It is used by all EDs participating in Relay, including the study sites. ED staff are instructed to call the Relay hotline as soon as they become aware of a patient presenting for nonfatal opioid OD, to help ensure that there is adequate time for both study procedures and (if randomized to the Relay arm) delivery of the Relay intervention.

Assignment of the RA. The Relay hotline operator contacts the appropriate research staff member based on date and time of day according to a call schedule that will be provided to the hotline. The

PM/RC/ or RAs will receive information that the Relay hotline routinely collects to facilitate locating the patient in the ED (e.g., patient name, referring provider, location or bed number). The RA will determine whether the patient is already an active Relay participant and/or has already participated in the study and proceed according to study eligibility guidelines. The appropriate RA for the study site will go directly to the ED, arriving within 1 hour (typically within 15-45 minutes depending on study site). Upon arrival, the RA will confirm with ED staff that the patient is medically stabilized (as needed), and then approach the bedside and introduce themselves to the patient. After requesting permission from the patient to provide information about the study, the RA will explain the study procedures and assess eligibility. Clear protocols for RAs will be specified in the study SOP/MOP.

Screening for Eligibility. RAs will follow a standardized series of questions on REDCap using a study iPad (see attachment) to screen patients for study eligibility. If there is any question about a patient's capacity to provide informed consent, RAs will consult the ED treating team (e.g., attending physician) and/or complete the UBACC (see attachment), a brief consent capacity quiz that has been previously validated and used in other ED studies.[57] If the patient is not eligible for the study, the RA will call the Relay peer navigator to inform him/her of a patient who is not eligible for the study but who could potentially receive Relay services (apart from the study).

Enrollment and informed consent. RAs will enroll and obtain consent from eligible patients for participation in the research activities. The RA will explain the study procedures and incentive schedule, answer any questions, and offer enrollment. Patients will be informed that the decision to participate in the research is voluntary and will not affect the medical care they receive from the ED. If the patient is willing to participate and signs the informed consent form, only then will study staff commence collecting additional information. The informed consent process will be conducted in-person on a study iPad using REDCap (using electronic consent/signature). The participant will be given a paper or e-mailed copy (through REDCap) of the informed consent form and key information sheet. If the patient declines to participate in the study, the RA will call the Relay peer navigator to inform him/her of the patient who has declined to participate in the study but who could potentially receive Relay services (apart from the study, as part of standard DOHMH Relay practice).

Randomization. Patients who meet eligibility criteria and agree to participate in the study will be randomized to the SDC vs. Relay arm. The RA will use an electronic system (REDCap) to create a random assignment to a treatment arm. Randomization uses a 1:1 ratio and is stratified by site; permuted blocks with variable blocks of size 4 and 6 will be used to maintain balance over time and prevent prediction of assignment by the RA. Upon randomization, the RA will immediately inform the participant of the treatment arm assigned. For Relay arm participants, the RA will then inform the Relay peer navigator by phone. If the participant is assigned to the Relay arm, the peer navigator will generally arrive at the ED within 30 minutes.

Baseline data collection. RAs will administer a baseline self-reported assessment. RAs will also collect participant identifying information to allow future linkage with administrative data, and detailed locator form information to enable study staff to contact participants for follow-up assessments. The locator form collects multiple forms of contact information for the individual (e.g., phone numbers, e-mail, social media, addresses, 'hang out' locations) and phone numbers for at least 3 individuals who will know how to reach them in the future. The locator form is based on procedures used successfully by our team in prior studies, and is a crucial component of the retention strategy. RAs will follow procedures to maximize participant privacy. If at any point during baseline data collection an ED provider needs to provide clinical care to the patient, the RA will pause study procedures so as not to interfere with clinical care.

Because participant identifiers and locator form information are not sensitive to the Relay intervention, RAs can collect this information at the time that is most appropriate to maximize patient flow and ensure peer navigators can engage patients. As much as possible, RAs will complete the baseline assessment prior to participant receipt of the Relay intervention (for participants in the Relay arm).

Administration of Relay intervention or SDC. Relay peer navigators will deliver the ED portion of the Relay intervention for participants in the Relay arm. For participants in the SDC arm, RAs will ensure that,

at a minimum, participants receive (from the RA) OEND, a list of treatment programs, and an informational handout about Relay. RAs will document the number of naloxone kits (1-2) distributed as described in Section 14.1. Participants will receive other site-directed care as specific to the particular ED and/or care provider (not under the control of the study). See other sections of this protocol for a detailed description of the Relay intervention and SDC conditions.

Exit survey. Near the end of the ED stay, the RA will complete an exit survey recording what intervention elements were received by participants. If needed (for example, in instances where the ED visit spans many hours), the RA may complete the exit survey by phone with the participant the day following the ED visit.

Wrap up. RAs will schedule the first follow-up assessment and will give participants a study contact card with the appointment information and information on how to contact the study team. Participants will be given the study incentive payment and a FAQ document about the Greenphire ClinCards. Within the week following the baseline visit the participant will be mailed a thank you card.

Hybrid Baseline visits due to COVID-19
Due to COVID-19, baseline visits may be conducted in a hybrid manner in order to minimize staff time in the ED. In this instance, RAs would screen, enroll, consent, and randomize patients in person in the ED. They would then conduct the survey section of the baseline visit virtually from a private office location adjacent to the ED using iPads (one iPad will be handed to the participant) using video conferencing with a secure, HIPAA compliant platform (WebEx).

7.3.2 Intermediate Visits

Acceptable time windows are given for each study visit, but attempts will be made to conduct the visit as close as possible to the intended day (+/- 3 days when possible).

Visit 2 (Day 30 +14/-7) (*All participants, conducted by RA or PM)

- RA administers structured interview over the phone or in person (30-40 minutes).
- Study incentive payment added to ClinCard.

Visit 3 (Day 90 +30/-14) (*All participants, conducted by RA or PM)

- RA administers structured interview over the phone or in person (30-40 minutes).
- Study incentive payment added to ClinCard.

7.3.3 Final Study Visit

Final Study Visit (Visit 4, Day 180 +30/-14) (*All participants, conducted by RA or PM)

- RA administers structured interview over the phone or in person (30-40 minutes).
- Participant is provided with final study incentive payment via ClinCard.
- Any questions about the study are answered and participant is given contact information for the study if needed.

7.3.4 Withdrawal Visit

Participants may choose to withdraw from the study by informing the PI in writing. There will not be a separate withdrawal visit. See Section 5.7 for additional details.

7.3.5 Unscheduled Visits

Qualitative interviews with patients: A subset of study participants will be invited to participate in semi-structured qualitative interviews. Purposeful selection will be used to choose approximately 12 English-speaking participants with a variety of experiences and perspectives to contribute to the interviews. The purpose of the qualitative interviews is to explore barriers and facilitators to delivery and sustainability of the Relay program. Interviews will be conducted one-on-one with an interviewer trained in qualitative research techniques. At the qualitative interview contact the interviewer will review the verbal consent

This material is the property of the NYU School of Medicine and NYU Langone Health. Do not disclose or use except as authorized in writing by the study sponsor

script, attain verbal consent for participation and audio recording, and complete a one-on-one in-depth interview in which the interviewer uses an interview guide. Interviews will be digitally recorded and transcribed. Basic characteristics of interview participants (e.g., age, race/ethnicity, gender) will also be recorded, and the interviewer will record field notes at the conclusion of the interview with any additional pertinent observations. Qualitative interview participants will receive an incentive payment at the conclusion of the interview.

Relay contacts: For participants in the Relay intervention arm only, the peer navigator stays in touch by phone for up to 90 days. In the Relay program, certain follow-up call attempts are mandated but otherwise may vary. The participant is encouraged to call or text the peer navigator any time if he/she has questions or unmet service needs. In these phone contacts, the peer navigator ‘checks in’ with the participant, monitors steps taken by the participant to achieve personal goals, and provides encouragement and support. Peer navigators log the number, type, and duration of contacts, which comprise the intervention ‘dose.’ Participants in the SDC arm will receive a card or flyer with information about Relay and a telephone number they can call to receive Relay services at any time after their ED visit. DOHMH will track any Relay services provided in this patient-initiated manner so that we can account for this in our analysis.

Additional unscheduled visits: Participants in both the SDC and Relay intervention arms will be given a study phone number and may reach out to the RA at any point during the study period. All such unscheduled encounters will be documented by the RA using a designated study form. RAs will also attempt to contact study participants between visits to confirm contact information and intermediate and final study appointment information. RAs will mail participants appointment reminder letters. If study staff is still unable to reach the participant after multiple attempts of contact, and the participant has given permission to do so, RAs may mail a letter to the participant’s contacts asking to help our study staff get in contact with the participant. Additionally, RAs will mail participants a thank you card and a holiday card when applicable. Finally, if participants present to the ED after a repeat opioid OD in which the Relay hotline is activated, RAs will attempt to visit the participant in the ED if possible (e.g., if an RA is available) to confirm contact information and visit appointments (or to conduct one of the scheduled intermediate or final study visits if within the appropriate time window).

7.4 Concomitant Medications, Treatments, and Procedures

All participants will receive care in the ED as they normally would during their baseline ED visit. Medications, treatments, procedures, and other care are at the discretion of the ED treatment team and will not be influenced by the study.

7.5 Justification for Sensitive Procedures

7.5.1 Precautionary Medications, Treatments, and Procedures

Not applicable.

7.6 Prohibited Medications, Treatments, and Procedures

Not applicable.

7.7 Prophylactic Medications, Treatments, and Procedures

Not applicable.

7.8 Participant Access to Study Intervention at Study Closure

After the 12 month study follow-up period (for the primary outcome analysis) has concluded, the DOHMH will attempt to contact participants who had been assigned to the SDC arm to offer Relay services. Additionally, after study closure all participants will continue to be eligible for Relay services should they present again to an ED participating in the Relay program. Study participants will still be able to access all

existing community/social services for which they are eligible and ED/health care at the end of the study as they would if they had not participated in the study.

8 Assessment of Safety

The Data and Safety Monitoring Plan submitted to the CDC is included as an attachment. Key elements are summarized below.

8.1 Specification of Safety Parameters

The following safety parameters are study endpoints:

- Opioid-involved OD (fatal and nonfatal)
- Substance use-related ED visit

These study endpoints will be captured using REDCap electronic data capture from interim and final study visit questionnaires, as well as at 12 months from administrative data analysis. Reports including these endpoints will be produced and discussed at regular DSMB meetings. Given the morbidity of the population being studied, we expect that these study endpoints will occur not infrequently. The stopping rule for the study will be based on the objective, serious endpoint of OD mortality and are specified in Section 8.7 and the DSMP which will be approved by the study sponsor.

This is a minimal risk study; therefore, no causally related AEs or SAEs are anticipated. AEs and SAEs that are not study endpoints are described in the sections that follow. Event impact will be formally and informally monitored and formally documented, and the MPIs will implement steps to prevent future similar events from occurring.

8.1.1 Definition of Adverse Events (AE)

IRB required text: An ***adverse event*** (AE) is any symptom, sign, illness or experience that develops or worsens in severity during the course of the study. Intercurrent illnesses or injuries should be regarded as adverse events. Abnormal results of diagnostic procedures are considered to be adverse events if the abnormality:

- results in study withdrawal
- is associated with a serious adverse event
- is associated with clinical signs or symptoms
- leads to additional treatment or to further diagnostic tests
- is considered by the investigator to be of clinical significance

Study specific text: For the purpose of this study, the following events will <u>not</u> be reported as AEs:

- Mild unrelated event.
- Moderate unrelated event. This would typically include physical events that were considered unrelated to study participation.
- Substance use events expected for the population being studied, including:
    - Worsening of substance use
    - Need for higher level of care
    - Signs and symptoms of substance use disorder or withdrawal
    - Drug or alcohol craving
    - Medical events, including but not limited to injuries, overdose, and medical conditions, that are directly or indirectly related to substance use

8.1.2 Definition of Serious Adverse Events (SAE)

Serious Adverse Event

IRB required text: Adverse events are classified as serious or non-serious. A ***serious adverse event*** is any AE that is:

- fatal
- life-threatening
- requires or prolongs hospital stay
- results in persistent or significant disability or incapacity
- a congenital anomaly or birth defect
- an important medical event

Important medical events are those that may not be immediately life threatening, but are clearly of major clinical significance. They may jeopardize the subject, and may require intervention to prevent one of the other serious outcomes noted above.

All adverse events that do not meet any of the criteria for serious should be regarded as ***non-serious adverse events***.

Study specific text: Given the population being enrolled in the study, it is expected that most participants will continue to have substance use, several may have overdose, and many may have hospitalizations or other treatments related to their substance use. We will be assessing these events at the 1-, 3-, and 6-month participant interim visits and at the 12-month administrative data abstraction. We will also track any events that we learn of between these time periods, which may occur if participants reach out to the study team. Events will not be individually reported as AEs or SAEs unless they are also judged to be related to the study, as further described in the sections that follow. Frequency of events, however, will be tracked and reported at regular DSMB meetings.

8.1.3 Definition of Unanticipated Problems (UP)

Unanticipated Problems Involving Risk to Subjects or Others
Any incident, experience, or outcome that meets all of the following criteria:

- Unexpected in nature, severity, or frequency (i.e. not described in study-related documents such as the IRB-approved protocol or consent form, the investigators brochure, etc.)
- Related or possibly related to participation in the research (i.e. possibly related means there is a reasonable possibility that the incident experience, or outcome may have been caused by the procedures involved in the research)
- Suggests that the research places subjects or others at greater risk of harm (including physical, psychological, economic, or social harm).

8.2 Classification of an Adverse Event

8.2.1 Severity of Event

The following guidelines will be used to describe AE severity.

- **Mild** – Events require minimal or no treatment and do not interfere with the participant’s daily activities.
- **Moderate** – Events result in a low level of inconvenience or concern with the therapeutic measures. Moderate events may cause some interference with functioning.
- **Severe** – Events interrupt a participant’s usual daily activity and may require systemic drug therapy or other treatment. Severe events are usually potentially life-threatening or incapacitating.

8.2.2 Relationship to Study Intervention

The clinician’s assessment of an AE's relationship to study intervention is part of the documentation process. If there is any doubt as to whether a clinical observation is an AE, the event should be reported.

All AEs must have their relationship to study intervention assessed. In a clinical trial, the study intervention must always be suspect. To help assess, the following guidelines are used.

- **Related / Possibly Related** – The AE is known to occur with the study intervention, there is a reasonable possibility that the study intervention caused the AE, or there is a temporal relationship between the study intervention and event. Reasonable possibility means that there is evidence to suggest a causal relationship between the study intervention and the AE.
- **Not Related** – There is not a reasonable possibility that the administration of the study intervention caused the event, there is no temporal relationship between the study intervention and event onset, or an alternate etiology has been established.

8.2.3 Expectedness

Dr. Doran and Dr. McNeely will together be responsible for determining whether an AE is expected or unexpected (also called unanticipated), with input from the study DSMB. An AE will be considered unexpected if the nature, severity, or frequency of the event is not consistent with the risk information previously described for the study intervention.

8.3 Time Period and Frequency for Event Assessment and Follow-Up

The occurrence of an AE or SAE may come to the attention of study personnel at study visits (1-, 3-, and 6- months after baseline) during study procedures as specified including participant surveys and interviews. Participants may also provide unsolicited information on AEs or SAEs during defined study visits or interim contacts. AEs or SAEs may also become apparent at the time of the final study administrative data review (12 months after baseline). Participants will be allowed to report problems, whether related or potentially related to study participation, to study personnel at and between scheduled intervention sessions and assessments. They will be instructed on how to contact study personnel should problems occur during intervals between visits. AEs or SAEs that are assessed through standard study surveys will be recorded in REDCap. Other events not captured in REDCap will be documented on an internal events log form.

8.4 Reporting Procedures – Notifying the IRB

The PI will report the following types of events to the IRB and DSMB:

- AE or SAE <u>unanticipated AND related to the study</u>. Study-specific descriptions of what will and will not be considered AEs or SAEs are provided in Sections 8.1.1 and 8.1.2.
- Other unanticipated problems involving risks to study participants or others.

The following events will also be treated as Reportable Events, as required by the IRB: an unresolved participant complaint that indicates a potential increased or unexpected risk; new information that presents a change to the risks or potential benefits; a major deviation from the IRB approved protocol.

Any departure from procedures and requirements outlined in the protocol will be classified as either a major or minor protocol deviation. The difference between a major and minor protocol deviation has to do with the seriousness of the event and the corrective action required. A minor protocol deviation is considered an action (or inaction) that by itself is not likely to affect the scientific soundness of the investigation or seriously affect the safety, rights, or welfare of a study participant. Major protocol deviations are departures that may compromise the participant safety, participant rights, inclusion/exclusion criteria or the integrity of study data and could be cause for corrective actions if not rectified or prevented from re-occurrence. Sites will be responsible for developing corrective action plans for both major and minor deviations as appropriate. Major protocol deviations will be reported to the IRB. Major and minor protocol deviations and corrective actions implemented will be documented in study records as further described in the MOP/SOP.

Events will be reported according to the NYU Langone Health IRB's reporting guidelines.

CONFIDENTIAL
This material is the property of the NYU School of Medicine and NYU Langone Health. Do not disclose or use except as authorized in writing by the study sponsor

8.4.1 Adverse Event Reporting

AEs (see section 8.1.1) that are harmful, unanticipated, and related to the study will be reported to the IRB using the appropriate forms via Research Navigator within 24 business hours of becoming known to the PIs. Additionally, such AEs will be recorded on a summary report provided yearly to the IRB at the time of study continuation. The PI will have primary responsibility for reporting AEs, with assistance from the study staff.

8.4.2 Serious Adverse Event Reporting

SAEs (see section 8.1.2) that are unanticipated and related to the study, or any deaths of study participants *related to study procedures* will be reported to the IRB and DSMB as quickly as possible after becoming known to the PI, and within 24 business hours. Reporting will be completed using the appropriate forms via Research Navigator. When appropriate and possible the PI will also attempt to discuss by phone with IRB staff. Serious adverse events uncovered during the study that are not reportable because they are not related to the research but are harmful and unanticipated will be communicated to the IRB at the time of continuing review in summary or aggregate form, to the DSMB in aggregate form at each scheduled meeting, and to the Program Officer at CDC in aggregate/summary form in the annual Progress Report. Non-harmful/expectable events are not recorded or reported to the IRB. When an event is reported, it will be documented on an internal Events Log Form described in the study MOP/SOP. The Events Log Form will assess the details, seriousness, and outcome of the event. Event impact will be formally and informally monitored and formally documented, and the MPIs will implement steps to prevent future similar events from occurring. Additionally, SAEs will be recorded on a summary report provided yearly to the IRB at the time of study continuation. The PIs will have primary responsibility for reporting SAEs, with assistance from the study staff. When an event is reported, it will be documented on an internal Events Log Form described in the study MOP/SOP. The Events Log Form will assess the details, seriousness, and outcome of the event. Event impact will be formally and informally monitored and formally documented, and the MPIs will implement steps to prevent future similar events from occurring if possible.

8.4.3 Unanticipated Problem Reporting

In addition to AE and SAE reporting as specified above, other unanticipated problems (UPs) involving risks to study participants or others will be reported to the IRB and DSMB within 72 business hours of becoming known to the PI. UP reports will include the following information:

- Protocol identifying information: protocol title and number, PI's name, and the IRB project number;
- A detailed description of the event, incident, experience, or outcome;
- An explanation of the basis for determining that the event, incident, experience, or outcome represents an UP;
- A description of any changes to the protocol or other corrective actions that have been taken or are proposed in response to the UP.

8.4.4 Reporting of Pregnancy

Not applicable. Pregnancy will not be followed or tested for within the context of this study. As noted, pregnant women are ineligible for study enrollment. Pregnancy at the time of enrollment is based on self-report or provider report; women will not be tested for pregnancy prior to enrollment in the study, nor will their clinical data be reviewed. However, if a participant becomes pregnant during the course of the study they may remain in the study.

8.5 Reporting Procedures – Notifying the Study Sponsor

Dr. Doran and Dr. McNeely will report the Reportable Event to the study's Program Officer at the CDC by phone or email within 24 business hours of the event becoming known and follow up with a written report (by email) within 72 hours, detailing any additional information and whether or not the event is related to participation in the study or may affect future participation in the study. They will make an initial report to the Chairperson of the DSMB within 24 hours of learning of the event, followed up with a written report

within 72 hours. Next, the DSMB will review the Reportable Event (usually on the phone or via email) and make a report via email to the PI, who will forward the report to the IRB and Program Officer at the CDC.

Adverse events uncovered during the study that are not reportable because they are not related to the research but are harmful and unanticipated will be communicated to the IRB at the time of continuing review in summary or aggregate form, to the DSMB in aggregate form at each scheduled meeting, and to the Program Officer at CDC in aggregate/summary form in the annual Progress Report.

8.6 Reporting Procedures – Participating Investigators

For any reportable events, the PIs will inform participating Site PIs in writing (email) upon determination of a course of action for the event or within 5 business days of the even, whichever comes first.

8.7 Study Halting Rules

Decisions about halting or terminating the study will be made by the PIs in conjunction with the DSMB. The study will be halted if:

- There are significant deficiencies in study performance and/or quality control such that continuation of the study would be dangerous, unethical, or would not be expected to result in meaningful results despite modification.
- There are significant risks to participant safety as indicated by frequency of reportable serious adverse events or protocol violations that put participants at risk.
- The study stopping rule is met. The stopping rule will be based on the objective, serious, negative event of fatal opioid overdose. The study will be stopped if we observe a rate ratio of number of OD deaths in the SDC arm to number of OD deaths in the Relay arm of ≥3.0 and a one-sided p-value of <0.01, or a one-side p-value of <.01 when the rate ratio is undefined due to zero deaths in the Relay arm. The DOHMH will provide the study investigators with data on any OD deaths for study participants biannually. The DSMB will review all overdose deaths of study participants at each biannual DSMB meeting. The DSMB will use the overdose death data to determine whether the stopping rule has been met at each biannual DSMB meeting after at least 80 total participants have been enrolled in the study, at which point differences between the study arms can be determined with reasonable statistical precision.

In the event that the study is halted, researchers will attempt to contact participants to inform them and potentially offer additional services.

8.8 Safety Oversight

It is the responsibility of the Principal Investigators to oversee the safety of the study overall and the responsibility of the Site Principal Investigators to oversee safety of the study at their sites. This safety monitoring will include careful assessment and appropriate reporting of adverse events as noted above, as well as implementation of a data and safety monitoring plan.

The DSMB is charged with monitoring the safety of study participants and quality of data. The DSMB will review the study protocol and recommend changes that may improve the protocol, as needed. Throughout the duration of the study, the DSMB will revisit the protocol to identify any emerging issues and recommend improvements and monitor the safety of study participants by assessing adverse event and other event reports. The DSMB will meet semi-annually to review interim analyses and efficacy reports to identify unanticipated or pre-specified harm or benefits that may be attributable to study activities. The Board will recommend any necessary modifications to the protocol or early termination of the study should overwhelmingly significant benefits or risks become apparent, or if they deem that the trial cannot be completed successfully.

The DSMB has five main objectives:

1) Protocol review—to review and evaluate the research protocol and plans for data and safety monitoring before data collection begins, recommending any needed modifications to study protocols and finalizing the particulars of the DSMP; to review subsequent protocol changes proposed by the investigators and recommend any needed changes prior to implementation as well as an implementation timeframe. The Sponsor's Program Officer will be informed of any changes recommended by the DSMB.

2) Participant safety—to review therapeutic results, unanticipated consequences, event impact, adverse events, and Reportable Events, formulating recommendations to continue, amend, or terminate the study based on ad hoc interim analysis for safety and/or efficacy and according to established criteria (e.g., recommend termination because to continue the comparison treatment group in the face of overwhelming benefit accrued from the experimental intervention would be unethical).

3) Study performance—to review study performance (e.g., eligibility of participants, accrual and follow-up rates, participation and retention rates, adherence to randomization protocol, program fidelity measures, data quality control/validity) and recommend that the study continue as is, be amended to achieve improvements, or be terminated (according to established criteria) because meaningful results would not result, regardless of modifications.

4) Study efficacy—to review interim analyses of outcome data and cumulative statistical summaries to determine whether the trial should continue as originally designed, or be changed, or be terminated according to the established criteria for benefit-risk (favorable and unfavorable) and inability to answer the research questions.

5) Quality control—to review quality control information for data collection and for program fidelity as obtained by the investigators and reported at each scheduled meeting to ensure and preserve study integrity and credibility; to assess this information and formulate recommendations that the study continue as is, be amended, or be terminated according to established criteria.

The DSMB will meet its objectives by fulfilling the responsibilities listed below:

1) Becoming familiar with the research protocols and plans for data and monitoring.

2) Reviewing adverse event reports (in aggregate) for disproportionate distribution between the two treatment conditions or unexpected frequency of occurrence; reviewing adverse event reports (in aggregate) and evaluating for attribution to the intervention or to study activities; suggesting protocol modifications to improve participant safety. The Sponsor's Program Officer will be informed of any changes recommended by the DSMB.

3) Reviewing major proposed modifications to the study prior to their implementation (e.g., termination, increasing target sample size).

4) Reviewing interim analyses of outcome data and cumulative data summaries to determine whether the trial should continue as originally designed, should be changed, or should be terminated according to the established criteria.

5) Providing the MPIs with a verbal overview and a detailed written report following each DSMB meeting that describe deliberations and findings related to cumulative observations and that list the Board's recommendations related to continuing, changing, or terminating the trial (the MPIs are responsible for distributing the written report to the appropriate parties). This report will be forwarded to the Sponsor's Program Officer. Any report containing findings that the frequency or magnitude of harms or benefits may be different than initially presented to the IRB will be reported to the IRB.

9 Clinical Monitoring

Clinical site monitoring is conducted to ensure that the rights and well-being of human subjects are protected, that the reported trial data are accurate, complete, and verifiable, and that the conduct of the trial is in compliance with the currently approved protocol/amendment(s), with GCP, and with applicable regulatory requirement(s).

Ongoing monitoring will occur at all study sites. Monitoring will be conducted in collaboration between the study PIs, the site PIs / Clinical Leads, the Project Manager, and the Research Assistants. The primary purpose of monitoring will be to ensure that all study activities are being conducted in accordance with the study protocol. Site monitoring will also ensure that the study is not posing abnormal risk to study participants or other ED patients. Monitoring will occur via regular in-person direct supervision by the Project Manager and Site PIs / Clinical Leads. Frequency of monitoring will be highest in the initial study start-up period and will decrease over time. The PIs and Study Site PIs / Clinical Leads will have regular monthly meetings at which any non-urgent site-specific issues can be discussed.

Monitoring will also occur by careful, regular review of all collected study participant information. Study participant consents and assessments are being collected at all study sites using REDCap that is centrally managed by NYU. As such, NYU study staff will have instant ability to review data collected at study sites. Data QA (of 100% of collected data) will be conducted by the PM under direction of the PIs weekly and will include independent evaluation by Study Site to ensure no irregularities.

Any irregularities or other issues with study sites will be discussed between the PIs and Site PIs / Clinical Leads to determine reasons for such issues and an action plan.

10 Statistical Considerations

10.1 Statistical and Analytical Plans

A detailed statistical and analytical plan will be finalized in consultation with the study biostatistician and determined prior to study outcomes analysis. Preliminary approaches are described below but some details of specific statistical analysis plans may change upon biostatistics consultation and/or as the study progresses; no changes in the analysis plan would change the risk to study participants. For minor statistical plan changes, protocol modification approval will not be sought from the IRB.

10.2 Statistical Hypotheses

We hypothesize that ED patients receiving the Relay peer navigator intervention after a nonfatal OD will have significantly fewer opioid-related adverse events (opioid OD and substance use related ED visits) in the next 12 months compared to patients receiving site-directed care.

10.3 Analysis Datasets

The primary dataset will contain all randomized study participants and will include data both from participant assessments (at baseline, 1-, 3-, and 6-months) and from a 12-month administrative data extraction/review. The primary analysis will be intent-to-treat (ITT). Depending on the amount of crossover between study arms other analyses may be conducted upon consultation with the study biostatistician.

Secondary datasets will include the Relay program data as maintained by the DOHMH and a qualitative interview dataset (audio recordings which will be transcribed). Of note, Relay program data forms were developed and are administered by DOHMH. Relay program data forms may be changed by DOHMH without notice, as this is an element of the DOHMH-operated program not under the direct control of the researchers. Such modifications will not be submitted for IRB approval.

10.4 Description of Statistical Methods

10.4.1 General Approach

Two-arm RCT comparing site-directed care (SDC) to the Relay intervention among patients presenting to the ED with a nonfatal opioid-involved OD.

10.4.2 Analysis of the Primary Efficacy Endpoint(s)

The primary endpoint is the number of opioid-related adverse events in the 12 months following the index ED visit. Opioid-related adverse events include: any opioid-involved OD (fatal or nonfatal) or any other substance use-related ED visit. Overdose deaths are tracked using the DOHMH comprehensive database of NYC overdose deaths. Data on OD resulting in ED visits or other substance use related ED visits will be collected from administrative data at 12 months, as identified from visit chief complaint and/or diagnoses. We will collect additional self-reported data on OD using a questionnaire that has been used in prior studies. This questionnaire will be administered at each study visit. Participants will be asked whether the OD did or did not result in an ED visit. We will use this information to calculate an estimated 12 month opioid-related OD event count.

The primary outcome will be ascertained by administrative data abstraction with adjustment using self-report data as described above. Administrative data extraction will be completed by members of the study team who are blinded to participant arm assignment. Results for the primary outcome will be compared for independent data abstraction completed by two members of the study team; disagreements will be resolved either by consensus or by a study PI.

The primary analysis will use a Poisson generalized linear regression model to evaluate the intervention effect on the total number of opioid-related adverse events. We anticipate the availability of 12 months of follow-up data on all participants but, in the event of death or withdrawal from the study, we will adjust the follow-up time appropriately using an offset term. We will include adjustment for study site, as well as other baseline factors that may be unbalanced across arms. The primary effect measure will be the incidence rate ratio comparing Relay to SDC. Some participants may be routinely high utilizers of the ED, and this may lead to overdispersion of the count outcome; we will therefore use a negative binomial model instead of a Poisson model if warranted.

In summary, the primary outcome is “opioid-related adverse events,” which will include fatal and non-fatal opioid-involved overdose as well as substance use-related emergency department (ED) visits. ED visits and fatal overdoses in the 12-month period after baseline will be captured in administrative data sources. Other non-fatal opioid-involved overdose events will be captured by self-report in follow-up interviews through six months after baseline, and estimated for the second half of the 12-month follow-up period.

10.4.3 Analysis of the Secondary Endpoint(s)

Secondary outcome analysis. For secondary outcomes, we will use models similar to those for the primary outcome. Initiation of medication for opioid use disorder (MOUD) in the first 90 days will be evaluated using a logistic regression model with intervention arm as the primary predictor. In addition, we will explore multiple initiations of MOUD in the follow-up period using longitudinal generalized linear mixed models. These will use a logit link and incorporate random effects for participants to accommodate the correlation of repeated assessments. The OD risk behavior score will be evaluated using mixed effects models for continuous longitudinal data, incorporating random effects for participants as described. Item responses to the OD risk behavior questionnaire will be summed; higher scores indicate a higher number and/or frequency of risk behaviors. Time to first subsequent OD will be evaluated using Cox proportional hazards models. Frequency of ED visits for a) opioid OD, b) other substance use related reason, and c) any cause will be evaluated with Poisson regression models as described for the primary outcome. For all models, we will assess validity of assumptions regarding distributional characteristics of the outcomes and find suitable transformations if necessary. We will assess indicators of model fit and evaluate standard model diagnostics such as residual plots.

Qualitative interview analysis. One-on-one interviews will be conducted with approximately 12 patients,

This material is the property of the NYU School of Medicine and NYU Langone Health. Do not disclose or use except as authorized in writing by the study sponsor

10-12 ED providers, and 10 peer navigators, though if we find that important new concepts are emerging in late interviews we will continue interviews until theoretical saturation is reached. Interviews will follow a semi-structured guide with a ‘start list’ of key questions drawn from the theoretical model and CFIR domains and will allow for exploration of unanticipated themes. Interviews will be digitally recorded and transcribed. Given the immediate pragmatic goals of the analysis (i.e., to identify factors impacting implementation and fidelity) and the desire to quickly translate research findings into practice, the initial analysis of the interviews will use rapid turn-around qualitative methods such as creating templated summaries of interviews and conducting matrix analysis focused on key domains. These techniques are action-focused, time-limited, and facilitate sharing of results, while still being rigorous. A subsequent analysis will include line-by-line coding of all content from the transcripts using an *a priori* coding scheme based on the theoretical model and CFIR domains; researchers will additionally identify any relevant emergent themes. Coding will be done by a core team of 2–3 trained researchers, and findings will be discussed among the investigators to reach consensus on the main themes. ATLAS.ti or Dedoose will be used to assist with coding and data organization.

Relay programmatic data. Additional descriptive analyses will be conducted of Relay programmatic data (e.g., number and types of contacts with participants) to allow a fuller understanding of the Relay intervention.

10.4.4 Safety Analyses

AEs and SAEs will be recorded and reported as previously described.

10.4.5 Adherence and Retention Analyses

All participants will complete an RA-administered exit survey interview at the conclusion of the baseline visit (either while still in the ED or hospital, or by phone the following day as needed). The exit survey will assess what interventions the participant has received in the ED that are relevant to opioid OD (e.g. substance use screening, OD education, naloxone kit(s) received, treatment referral/follow-up offered). The exit survey interview will also ensure that SDC arm participants receive, at a minimum, OEND, a list of treatment programs, and a handout with Relay program information. RAs will need to check off boxes in the exit survey indicating that the participant has received these items. Participants will also be asked at follow-up assessments what additional Relay program services they have received in the interval since the last assessment.

We will routinely monitor data on number of potentially eligible patients, reasons for ineligibility, refusals and reasons for refusal, and retention/loss to follow-up at all follow-up assessment time points. These data will be compiled and discussed at weekly meetings between the Project Manager and PI(s).

10.4.6 Baseline Descriptive Statistics

Simple descriptive statistics will be examined for baseline characteristics of study participants, as collected on the baseline study survey. Baseline basic characteristics for Relay and SDC arm participants will be compared using bivariate analyses. We expect approximately equal distributions of baseline characteristics for the two study arms given random assignment. Significant differences in baseline characteristics between groups will be adjusted for in outcome analyses.

10.4.7 Planned Interim Analysis

10.4.7.1 Safety Review

Safety review is described in Section 5.8 Premature Termination or Suspension of Study and Section 8.7 Study Halting Rules. Safety endpoints will be monitored at least biannually, in conjunction with biannual DSMB meetings.

10.4.7.2 Efficacy Review

We do not plan formal interim analyses for efficacy.

CONFIDENTIAL
This material is the property of the NYU School of Medicine and NYU Langone Health. Do not disclose or use except as authorized in writing by the study sponsor

10.4.8 Additional Sub-Group Analyses

Mediators and moderators. Relay may impact primary and secondary outcomes indirectly, by means of its impact on putative mediators grounded in our conceptual model, including OD knowledge, motivation, social support, peer role modeling, stigma, barriers to services, and access to naloxone. Also, the intervention may not be equally effective for all participants. The following individual factors may modify the relation between the Relay intervention and outcomes: age, gender, race/ethnicity, sexual orientation, marital status, ZIP code, housing status, mental and physical health, substance use history, and criminal justice involvement.[118] Understanding mediators and moderators of the Relay intervention will be critically informative for future efforts to scale up and/or modify the program, or replicate it in other settings. We will examine Relay intervention effect mediation and moderation in structural equations models (SEMs). We will estimate SEMs to measure the degree to which the Relay intervention improves knowledge (OD, naloxone), motivation (naloxone, MOUD, other treatment), social support, peer role models, and access to naloxone kits, as well as the degree to which the intervention reduces stigma and perceived barriers to services. Also estimated will be the impact of these putative mediators on the risk of opioid-related adverse events and other outcomes. Models will be fit that include paths from Relay to mediators and outcomes as well as paths from mediators to outcomes. The magnitude and significance of the direct and indirect effects of Relay will be provided. Bootstrapping methods will be used to derive estimates of mediated (i.e., indirect) effects. Bootstrapping approaches to indirect effect inferences rely on repeated random sampling from the data with replacement (e.g., 10,000 times) and use the resulting sampling variability of the estimate as the basis for an interval estimate. To capture potential effect moderation (regarding both the effects of Relay on mediators and the effects of mediators on outcomes), interaction terms will be included in these SEMs. This approach, described by Preacher, Rucker, and Hayes (2007), allows multiple types of effect moderation including moderation of: 1) effect of Relay on mediator; 2) effect of mediator on outcome; 3) indirect effect of Relay on outcome via mediator; and 4) direct effect of Relay on outcome. The software package Mplus accommodates model constraints to facilitate estimation and testing of complex moderated mediation hypotheses. When significant moderator effects are detected, we will estimate and present direct and indirect effects for specific subgroups defined by the moderating variable (e.g., the indirect effect of Relay on overdose via increases in social support *among males).* In addition to moderated mediation analysis based on the standard SEM approach and products of coefficients, we will employ analysis of causal mechanisms grounded in the potential outcomes framework, using marginal structural models. This approach highlights assumptions needed to identify direct and indirect effects of interest: no unmeasured cofounders of the exposure (Relay) and outcome relation; no unmeasured confounders of the mediator and outcome relation; no unmeasured confounders of the exposure and mediator relation; and no measured or unmeasured confounders of the mediator and outcome relation affected by exposure. Because the Relay intervention is randomly assigned, the key issue for the proposed study is addressing confounding of the relation between mediators and outcomes. Because unmeasured confounding of relations between mediators and outcomes may remain despite attempts to measure and include known confounders in the models, sensitivity analysis will be undertaken to determine how the size of the correlation between error for the mediator model and error for the outcome model impacts inferences for direct and indirect effects. Analyses will be undertaken with Mplus (Version 8) and the medflex package in R.

Finally, we will assess the impact of intervention dose, measured using the number, type (phone or in-person conversation, accompaniment to appointments), and duration of contacts; this information is routinely logged by the Relay peer navigator. We will examine the impact of dose using instrumental variables (IV) regression; this approach can substantially increase the strength of causal inference about a variable (such as dose) not fully under experimenter control. IV regression requires a variable related to a dose, but not correlated with any other determinants of dose. Random assignment to study condition meets these requirements; thus, IV regression illuminates the intervention's effectiveness at different dose levels.

10.4.9 Multiple Comparison/Multiplicity

Not applicable.

10.4.10 Tabulation of Individual Response Data

All planned analyses use aggregate data. Individual data will not be published.

10.4.11 Exploratory Analyses

Participants will be asked about any services received from an outpatient medical or mental health provider, or from a harm reduction program; for those reporting receipt of any services, we will measure the frequency of contact and the nature of services received. We will additionally assess use of naloxone by participants and naloxone carrying by friends/family. Death from OD, opioid-related OD, and any cause will be tracked using Vital Statistics and/or National Death Index records (accessed by DOHMH) since prior research shows high rates of death, including those unrelated to OD, among patients following ED visits for non-fatal opioid OD.[48]

10.5 Sample Size

The sample size for this study is 350. Our preliminary data indicate the average number of opioid-related adverse events per year in SDC arm will be in the range of 2-3 events/person; this rate is substantiated in the literature.[19,50,52,58] We wish to be able to detect a decrease in this number of approximately 25% in the Relay arm, a modest effect size judged to be of public health significance. Using a Poisson regression model for the total number of events, 350 participants provide more than 80% power to detect this difference. Specifically, we have 83% power to detect a decrease in the mean event rate from 2.0 per year in SDC to 1.52 in Relay (an incidence rate ratio (IRR) of 1.32 and a 24% reduction). If average opioid-related adverse events in the SDC arm are lower than anticipated, we still have 80% power to detect a decrease in the mean event rate from 1.75 per year in SDC to 1.32 in Relay (IRR = 1.33; a 25% reduction). All calculations assume a two-sided, 0.05-level test, and conservatively include adjustment for possible overdispersion in the primary outcome (overdispersion parameter = 1.6). While formal power and sample size calculations were based on our primary outcome, given that secondary outcomes are generally more common than our primary outcome (e.g., all-cause ED visits) or are time to events expected not to be rare (e.g., time to opioid-involved OD), we expect to have adequate power for secondary outcome analysis as well.

10.6 Measures to Minimize Bias

10.6.1 Enrollment/Randomization/Masking Procedures

At the enrollment visit, the RA will use an electronic system (REDCap) to trigger assignment to a treatment arm. Randomization will use a 1:1 ratio and is stratified by site; permuted blocks with variable blocks of size 4 and 6 will be used to maintain balance over time and prevent prediction of assignment by the RA. Upon randomization, the RA will immediately inform the participant of the treatment arm assigned.

While the RA conducting the baseline assessment cannot be blinded to the intervention assignment (since it will occur during the same ED visit as the intervention), whenever possible a different RA will be assigned to conduct follow-up interviews in an attempt to maintain blinding. Blinding may be imperfect since participants may reveal their intervention assignment, including based on their answers to the follow-up assessments. Participants themselves cannot be blinded to the treatment condition due to the nature of the intervention. Study staff conducting the administrative data abstraction for the primary study outcomes will be blinded to the intervention assignment. Study analytic staff are blinded to treatment condition until the conclusion of the trial and finalization of the analytic database.

10.6.2 Evaluation of Success of Blinding

Not applicable.

10.6.3 Breaking the Study Blind/Participant Code

Not applicable. For any reportable events, the participant's study arm will be noted.

This material is the property of the NYU School of Medicine and NYU Langone Health. Do not disclose or use except as authorized in writing by the study sponsor

11 Source Documents and Access to Source Data/Documents

Source data is all information, original records of clinical findings, observations, or other activities in a clinical trial necessary for the reconstruction and evaluation of the trial. Source data are contained in source documents. Examples of these original documents, and data records include: hospital records, clinical and office charts, laboratory notes, memoranda, subjects' diaries or evaluation checklists, pharmacy dispensing records, recorded data from automated instruments, copies or transcriptions certified after verification as being accurate and complete, microfiches, photographic negatives, microfilm or magnetic media, x-rays, subject files, and records kept at the pharmacy, at the laboratories, and at medico-technical departments involved in the clinical trial.

For this study documents aside from the study-specific documents as described in this protocol are minimal. Study baseline and follow-up assessments and other participant information collected at baseline will be collected and maintained using REDCap. REDCap maintains a log of any changes to this information. Consent forms will also be collected on REDCap. Relay programmatic data is maintained by DOHMH on a separate system per their existing protocols.

Access to study records will be limited to the study team, including DOHMH study team members as governed by an executed DUA. The investigator will permit study-related monitoring, audits, and inspections by the IRB/EC, the sponsor, government regulatory bodies, and University compliance and quality assurance groups of all study related documents (e.g. source documents, regulatory documents, data collection instruments, study data etc.). The investigator will ensure the capability for inspections of applicable study-related facilities (e.g. pharmacy, diagnostic laboratory, etc.).

Participation as an investigator in this study implies acceptance of potential inspection by government regulatory authorities and applicable University compliance and quality assurance offices.

12 Quality Assurance and Quality Control

Multiple steps will be taken toward quality assurance (QA) and quality control (QC).

- All study staff will be thoroughly trained both in overall GCP and human subjects protections and in study-specific procedures.
    - Formal training will occur prior to the study beginning recruitment.
    - RAs will be supervised until they are able to conduct study procedures independently.
    - Continuous training will occur in weekly staff team meetings.
- RAs will be closely supervised by the PM, with PI oversight.
- MOP and SOP documents will clearly outline all study procedures, including QA/QC procedures.
- Multiple QA/QC protections are built into REDCap data collection software being used for this study, including forced question entry (i.e., specified questions cannot be left blank), automatic logging of all activities in REDCap including any entry changes, and study staff permission rules.
- Weekly QA/QC reviews conducted by the PM and reviewed/discussed with the PI will include:
    - Review of numbers of patients approached, eligible/ineligible, refused, participated.
    - Review to ensure that all eligibility classifications were made correctly.
    - Review for missing or out of range data.
    - Review for completeness of forms and data.
    - Review of exit surveys for adherence to study protocols.
    - Review of any safety issues.
    - Review of any potential QA/QC issues identified by any study staff member (which did not warrant more immediate discussion).

QC procedures will be implemented beginning with the data entry system and data QC checks that will be run on the database will be generated. Any missing data or data anomalies will be communicated to the site(s) and/or individual RA(s) for clarification/resolution.

13 Ethics/Protection of Human Subjects

13.1 Ethical Standard

The investigator will ensure that this study is conducted in full conformity with Regulations for the Protection of Human Subjects of Research codified in 45 CFR Part 46.

13.2 Institutional Review Board

The protocol, informed consent form(s), recruitment materials, and all participant materials will be submitted to the IRB for review and approval. Approval of both the protocol and the consent form must be obtained before any participant is enrolled. Any amendment to the protocol will require review and approval by the IRB before the changes are implemented to the study. All changes to the consent form will be IRB approved; a determination will be made regarding whether previously consented participants need to be re-consented.

13.3 Informed Consent Process

13.3.1 Consent/Assent and Other Informational Documents Provided to Participants

Consent forms describing in detail the study intervention, study procedures, and risks, as well as key information sheets providing a concise summary of these factors, are given to the participant. Documentation of informed consent is required prior to starting study procedures. The following consent materials are submitted with this protocol:

- Relay Consent Form – Main Study
- Relay Key Information Sheet – Main Study
- Verbal Consent Form – Qualitative Interview Patients
- Verbal Consent Form – Qualitative Interview Providers

13.3.2 Consent Procedures and Documentation

Those eligible will be invited to complete the informed consent process. The RCT consent documents will be in English and Spanish, and consent will be obtained by trained, bilingual RAs. Consent will be obtained using e-consent on Open REDCap. A copy of the signed e-consent will be provided to the potential participant in one of three ways: 1) signed electronic consent copy will be e-mailed if the participant has an e-mail address and agrees to receive an e-mailed version of the consent copy, 2) signed electronic consent copy will be printed and provided to the participant, or 3) if the participant does not have or does not want e-consent e-mailed to them and printing isn't available at the study site, he/she will be asked to sign a paper version of the consent form, which will be provided to them to keep. Multiple steps will be taken to ensure participant privacy during the consent process, including: asking any visitors to leave the area (though visitors will be allowed to stay for the consent process if preferred by participants); using a private room or treatment bay when available; research staff speaking in a quiet voice to avoid others overhearing the conversation. Potential participants will be given a written consent form that includes required language regarding HIPAA and the Certificate of Confidentiality, as well as name and contact information of the PIs and of the institutional review board, a description of the study, the payment schedule, a description of potential risks and benefits, a statement of confidentiality, and an indication of the right to refuse or withdraw participation at any time without any consequence. The consent form for patients will include consent for accessing their personal health information in administrative data sources (e.g., Bronx RHIO and HealthIx RHIO and DOHMH mortality data) for 12 months following enrollment.

Informed consent will be obtained by the trained study researchers from all participants, per usual human subjects research procedures. RAs will review the IRB-approved consent form and key information sheet verbally and in detail, in simple and plain language, with potential participants. Participants will give signed informed consent prior to participation in all study activities. Study staff will emphasize that participation is voluntary, that participants are free to stop participation at any time and are free to refuse

to answer specific questions in any assessment and can decline participation in any intervention activity or assessment activity. Participants will be informed that their decision to participate or decline to enroll in the study or any aspect of the study will not affect any other services they receive in the ED or elsewhere.

Informed consent is a process that is initiated prior to the individual's agreeing to participate in the study and continues throughout the individual's study participation. Discussion of risks and possible benefits of participation will be provided to the participants and their families. Participants will have the opportunity to carefully review the written consent form and ask questions prior to signing. Study staff will answer any questions that may arise. The participants will have the opportunity to discuss the study with their surrogates or think about it prior to agreeing to participate. The participant will sign the informed consent document prior to any procedures being done specifically for the study. The participants may withdraw consent at any time throughout the course of the trial. The rights and welfare of the participants will be protected by emphasizing to them that the quality of their medical care will not be adversely affected if they decline to participate in this study.

The participant and the person obtaining informed consent will sign and date the informed consent document prior to any procedures being done specifically for the study. A copy of the signed electronic consent will be provided to the potential participant in one of three ways as described above. The electronic copy of the signed informed consent document will be stored electronically in the subject's research record.

The consent process, including the name of the individual obtaining consent, will be thoroughly documented in the subject's research record. Any alteration to the standard consent process and the justification for such alteration will likewise be documented. Regular audits will be performed to ensure that consent forms are fully and properly completed for all study participants.

Participant capacity and comprehension: Potential participants will be required to demonstrate adequate comprehension of the study and decision-making capacity to participate in the study. Only patients who have the capacity to give informed consent will be included in the study. Patients will not be included if they are too acutely intoxicated to give informed consent or otherwise medically unfit to participate, as determined by trained study staff. If there is any question about the patient's capacity to provide informed consent, study staff will: a) ask ED clinical providers (e.g., physician, nurse) for advice about whether the participant has capacity to provide consent, and/or b) complete formal assessment of decisional capacity for participation in research using the University of California, San Diego Brief Assessment of Capacity to Consent (UBACC). The UBACC has been previously validated and generally takes less than 5 minutes to administer.

Consent for Qualitative Interviews:
Patient participants, ED providers, and Relay Wellness Advocates will verbally consent to take part in the qualitative interview. The Qualitative Researcher will review the IRB-approved verbal consent form in English with each participant. The Qualitative Researcher will review a description of the study, a description of potential risks and benefits, a statement of confidentiality, and an indication of the right to refuse or withdraw participation at any time without any consequence the study requirements. The verbal consent will include explicit consent for an audio recording.

Consent processes, like the interviews, will take place by phone, video conferencing, or in person. Multiple steps will be taken to ensure participant privacy during the consent and interview process, including: requesting that the participant is in a private space; providing a private space for participants requesting to complete the interview in person; speaking in a quiet voice to avoid others overhearing the conversation.

13.4 Posting of Clinical Trial Consent Form

The informed consent form will be posted on the Federal website after the clinical trial is closed to recruitment, and no later than 60 days after the last study visit by any subject.

13.5 Participant and Data Confidentiality

All research data is stored in secure, password-protected storage drives maintained on a remote server by the NYU School of Medicine and accessible only to members of the research team. Access to research offices at NYU is restricted to research staff, and the buildings where these offices are located are secure and monitored by security staff. File drawers containing participant information are located only at the NYU research office. These files are kept locked when not in use, and only research staff members have keys to these file cabinets. When the researchers are collecting data at the clinical sites, they may hand-carry a minimum amount of participant information required for that day's study visits on site (ex: name and location for potentially eligible patients). At the end of each recruitment day, this information will be carried back to the research office and stored in a locked cabinet or placed in a confidential shred bin per protocols to be specified in the MOP/SOP. Personal health information required to carry out the study (e.g., name, contact information, medical record number) will be stored in a separate REDCap form from study assessments.

Administrative data: Existing administrative data sets will be secured according to standard protections at the institutions where the data are housed and maintained.

Audio recordings: Interviews will be recorded using Webex or the Rev Voice Recorder app. Interviews conducted on WebEx, will be recorded using WebEx. Interviews conducted on the phone, will be recorded using Rev. As a back-up recording, a digital audio recorder may be used. Digital audio files will be transferred to NYULH protected secure drives, which are HIPAA-compliant and protected from unauthorized access. Audio files will then be deleted from the Rev recording app or WebEx system. Researchers will provide audio files to a professional transcription company that meets privacy and security requirements for research (e.g., *Transcript Divas Transcription Services*). Audio files will be uploaded and transcripts downloaded from the transcription company's secure website via the study's password-protected account. The transcription company limits access to audio recordings to only those who need access to provide the transcription service, and only for that purpose. Following completion of transcription, only written transcripts will be used for analysis. Transcripts will not contain names or other identifying information, and data will be analyzed and reported anonymously. Audio recording files will be deleted once required data retention periods have passed.

Data security and disaster recovery: This study will use NYU Langone Health's extensive information technology (IT) infrastructure and the comprehensive policies and standard operation procedures (SOPs) established for information security and the protection of data. All data processing follows NYU Langone Health's strict security policies to ensure the privacy and confidentiality of patient data and guard against physical, accidental, or malicious loss of data or the hardware on which it resides. For example, all desktops and servers on the network run virus protection software that guard against the destruction of any data, and Network users cannot disable virus defense software. All NYU Langone Health servers are backed up using de-duplication technology. Data are stored on servers residing in the secured off-site data center, and NYU Langone Health has a comprehensive disaster recovery plan that allows for the expedient resumption of the processing of study data in the case of a disaster.

REDCap. The secure and password protected REDCap database will be used for the majority of study activities. Consent forms will be signed and structured assessments will be conducted in REDCap; the study will use electronic consent. REDCap was designed specifically to protect patient and research participant privacy and confidentiality while assisting investigators in conducting clinical research. System-level and application-level security features include SSL encryption of internet traffic (e.g., https pages), hosting in a secure data center with nightly backup, fine-grained control over user rights, detailed audit trails, record-locking, and de-identification functions for data export. Data can be downloaded from REDCap onto secure NYU School of Medicine maintained drives that have security protections suitable for research. Downloaded data will include minimum possible amount of participant identifying data. In cases when participant identifying information is downloaded it will be stored in separate files from participant study assessments.

Computers. All computerized data that are not in REDCap (transcripts, audio-recordings prior to deletion) are kept in secure network drives (in accordance with IT policy) that are password-protected and files are only labeled with study IDs when applicable.

Individual participants and their research data will be identified by a unique study identification number. This number will be used for all materials, assessments, audio recordings, and transcripts. The study data entry and study management systems used by clinical sites and by NYU research staff will be secured and password protected. At the end of the study, all study databases will be de-identified and archived at NYU.

As part of the informed consent process, participants will also be informed that all information they provide in study assessments/interviews is confidential, with the following exceptions: (1) If they are in imminent danger of committing suicide or homicide, the appropriate agency will be contacted (usually 911 will be called) or, if already in the emergency department, the participant's attending physician will be notified, and (2) if they are physically or sexually abusing someone, the Administration for Children's Services or other appropriate agency will be contacted.

Information about study subjects will be kept confidential and managed according to the requirements of the Health Insurance Portability and Accountability Act of 1996 (HIPAA). Those regulations require a signed subject authorization informing the subject of the following:

- What protected health information (PHI) will be collected from subjects in this study
- Who will have access to that information and why
- Who will use or disclose that information
- The rights of a research subject to revoke their authorization for use of their PHI.

In the event that a subject revokes authorization to collect or use PHI, the investigator, by regulation, retains the ability to use all information collected prior to the revocation of subject authorization. For subjects that have revoked authorization to collect or use PHI, attempts should be made to obtain permission to collect at least vital status (i.e. that the subject is alive) at the end of their scheduled study period.

Participant confidentiality is strictly held in trust by the participating investigators, their staff, and the sponsor(s) and their agents. No information concerning the study or the data will be released to any unauthorized third party without prior written approval of the sponsor. The study monitor, other authorized representatives of the sponsor, or representatives of the IRB may inspect all documents and records required to be maintained by the investigator. The clinical study site will permit access to such records.

The study participant's contact information will be securely stored for internal use during the study. At the end of the study, all records will continue to be kept in a secure location for as long a period as dictated by local IRB and Institutional regulations.

To further protect the privacy of study participants, a Certificate of Confidentiality is automatically granted for this research as it meets federal requirements for such a Certificate. This certificate protects identifiable research information from forced disclosure. It allows the investigator and others who have access to research records to refuse to disclose identifying information on research participation in any civil, criminal, administrative, legislative, or other proceeding, whether at the federal, state, or local level. By protecting researchers and institutions from being compelled to disclose information that would identify research participants, Certificates of Confidentiality help achieve the research objectives and promote participation in studies by helping assure confidentiality and privacy to participants.

13.5.1 Research Use of Stored Data

No human samples or specimens are being collected or stored for this study. Survey and other data being collected for this study will be handled as documented in Section 14 below.

13.6 Future Use of Stored Data

Study data generated from the present study will not be stored for future research.

14 Data Handling and Record Keeping

14.1 Data Collection and Management Responsibilities

Primary data collection from participants in the RCT will occur by approved, trained study RAs at the study EDs. Data collection will occur using REDCap on secure tablet computers (e.g., iPads), which will be password protected. Access to REDCap will only be granted to approved study personnel, each of whom will have a unique user ID and password to be used when collecting data. REDCap permissions and functionality will be restricted for RAs so that they do not have the ability to delete records or change forms. In addition to these protections, REDCap has other functionality that will ensure data collected are complete and reliable, including data entry field limitations and forced responses to indicated questions. REDCap also automatically maintains an audit log of all REDCap activity, including any changes made to records and who has made them. All information entered into REDCap is automatically saved in the NYULH REDCap project, accessible only to approved study personnel. All study sites will use the same approved NYULH REDCap project. For QA monitoring and data analysis, REDCap data will be downloaded in the form of Excel and/or statistical programming software files. These files will be password protected and saved in secure NYULH IT-managed network drive accessible only to the PI, PM, and any other approved personnel. Electronic consent forms will also be stored on REDCap. There are no paper questionnaires or other forms that will be saved from the RCT visits.

The PI and PM will monitor data collection regularly via the QA/QC procedures outlined in Section 12. Further, all RAs will receive standard training in study procedures and will complete supervised shifts until they are fully competent in all study data collection. The PM and PI will be available in real-time to answer any RA's questions about data collection. Further, weekly study team meetings will be used to continually reinforce standards of good data collection and management.

The primary outcome will be ascertained in part by data abstraction from a variety of administrative health databases, including primarily the Regional Health Information System (RHIO) databases, also called Bronx RHIO and Healthix. We will also use individual health system electronic medical records (which may be accessed through data warehouses or directly for sites participating in the study). Other sources of administrative data that may be used if data is incomplete in the aforementioned sources include Medicaid and SPARCS. Study participants will provide informed consent for the study investigators to access information about them in these datasets. Approval and use of these datasets will follow defined procedures for research specific to each source dataset. Data abstraction will occur directly into a REDCap secure data collection form. Data will only be accessed for study participants. RHIOs may request and receive from the study investigators a list of participants (including personally identifying information) in the study, to allow them to conduct a data match with RHIO data and share results with the investigators. All appropriate and applicable permissions will be obtained from the holders of administrative data outside the study sites prior to their use for research (e.g., completion of RHIO application for research use), and DUAs will be executed prior to data sharing as necessary.

Additionally, DOHMH collects Relay program data which it maintains in a programmatic database known as Maven. All data entered into Maven are stored in a secure, password protected, SQL Server Database on the DOHMH servers. Access is restricted to approved DOHMH staff and restricted access will be granted to approved NYU study team members. NYU study team members will enter limited participant information into Maven (see Attachment) for the following purposes: (1) participant identifying information (e.g., name, date of birth, race/ethnicity) so that DOHMH can match study participants to death records to allow ascertainment of the primary study outcome (Section 3.1) and also tracking for purposes of the study stopping rule (Section 8.7); (2) participant contact information so that the Relay program can contact SDC arm participants after the study is over to offer Relay services (see Section 6.1), and (3) information on NYU RA distribution of naloxone kits to SDC arm participants, as DOHMH is providing the

This material is the property of the NYU School of Medicine and NYU Langone Health. Do not disclose or use except as authorized in writing by the study sponsor

naloxone kits to NYU and is legally required to monitor their distribution. Study participants will provide informed consent for the NYU study team to share this information about them with DOHMH.

DOHMH also keeps data on overdose and other deaths in NYC and has other administrative health data that we may use for this study. For any data to be shared by DOHMH with the NYU study team, we will execute a Data Use Agreement (DUA) and/or Memorandum of Understanding (MOU) with DOHMH to govern data use and sharing prior to access or use of any of these data.

We will be providing a list of participant names and birthdates of people who have participated in the SDC arm to the Relay hotline call center. The call center will not be provided with any other participant information. Using a secure, password protected file transfer portal that only NYU and the Relay hotline call center will have access to, the PM or RC will transfer the list of participant names. This information will be provided securely to the Relay hotline call center because there is not a study RA on call 24/7 and therefore the call center needs an alternate way of knowing when a patient for whom Relay is activated is in the study SDC arm meaning a Wellness Advocate should not be dispatched to visit the patient (because this would cause crossover between study arms). This will only be relevant in the rare instances for which a study participant in the SDC arm has a repeat overdose visit to an emergency department participating in the study and the Relay hotline is called. In these cases a study RA will conduct an unscheduled visit or follow-up visit as appropriate (see Section 7.3) in the ED once available (if the participant remains in the ED) or will attempt to follow up by phone. The Relay hotline call center activities related to this study follow a comprehensive data privacy and security agreement with the NYC Department of Health and Mental Hygiene, which specify data security procedures, rules against data sharing, and other protections for privacy of the data. The Relay hotline call center will delete all participant lists after completion of the study follow-up period, one year after enrollment of the last study participant.

The study PIs will have ultimate responsibility for overseeing data collection and management. The PIs are responsible for ensuring the accuracy, completeness, legibility, and timeliness of the data reported.

14.2 Study Records Retention

Study records retention will be compliant with the NYULH ‘Policy on Retention of and Access to Research Data’ which mandates retention periods of 6 years after any reporting, publication or presentation, or for the time period specified in the Sponsored Research Agreement. No records will be destroyed without the written consent of the sponsor, if applicable. It is the responsibility of the sponsor to inform the investigator when these documents no longer need to be retained.

14.3 Protocol Deviations

A protocol deviation is any noncompliance with the clinical trial protocol, GCP, or MOP requirements. The noncompliance may be either on the part of the participant, the investigator, or the study site staff. As a result of deviations, corrective actions are to be developed by the site and implemented promptly.

These practices are consistent with ICH E6:

- 4.5 Compliance with Protocol, sections 4.5.1, 4.5.2, and 4.5.3
- 5.1 Quality Assurance and Quality Control, section 5.1.1
- 5.20 Noncompliance, sections 5.20.1, and 5.20.2.

Reporting of protocol deviations is described in Section 8.4. The site PI/study staff is responsible for knowing and adhering to their IRB requirements. Further details about the handling of protocol deviations will be included in the MOP.

14.4 Publication and Data Sharing Policy

The International Committee of Medical Journal Editors (ICMJE) member journals have adopted a clinical trials registration policy as a condition for publication. This study will be registered as a clinical trial on Clinicaltrials.gov.

A publication plan will be made by the MPIs and other applicable co-investigators. A draft publication plan will be made and discussed with Site Co-Is and other Co-Is and agreed upon within the first year of the study. The MPIs will have ultimate responsibility and decision-making authority regarding the publication plan. The International Committee of Medical Journal Editors (ICMJE) guidelines for authorship will be followed.

<u>Access to the data</u>. After analysis, the final dataset will be incorporated into a secure computer database as soon as possible, and no later than within one year of the completion of the funded project period. Datasets will be archived within the secure NYULH data center but curated to be accessible for data sharing as required. Datasets will be made available in a non-proprietary format. The research database will contain no PHI (protected health information), and all appropriate procedures consistent with the Office of Civil Rights' Privacy Rule (HIPAA) will be taken to preserve the anonymity of the records. As appropriate, the following steps will be taken to share and distribute the research data:

1. Results will be presented at scientific meetings in the form of posters and oral presentations.
2. Results will be published in a prompt fashion. We will fully comply with CDC policies on public access to publications. Further, de-identified data underlying publications will be made available at the time of the publication of the paper upon request, unless the dataset has already been made available as described.
3. The rights of human subjects will be preserved at all times, including privacy and confidentiality of human subjects. The dissemination and disclosure of research results and findings will use only de-identified data.
4. Data will be shared under confidentiality agreements with collaborators, partners, and sponsors in the project.
5. Agreements may also be used to protect intellectual property rights while exchanging information with collaborators, partners and the larger scientific community, including mutual confidentiality (non-disclosure) agreements.

Upon request, we will provide the research dataset to qualified investigators under a Data Use Agreement (DUA) that will require approval of the MPIs and each of the primary research team participating agencies (NYU, DOHMH). The DUA will be required to include: (1) a commitment to using the data only for research purposes and not to identify any individual participant; (2) a commitment to securing the data using appropriate computer technology; (3) a commitment to destroying or returning the data after analyses are completed; (4) reporting responsibilities; (5) restrictions on redistribution of the data to third parties or use for commercial purposes; (6) appropriate human subjects and institutional approvals on the part of the collaborator; and (7) proper acknowledgement of the data resources.

<u>Use of data standards</u>. Where possible, we will use common data elements and data standards for the collection of data, as specified by the NIH Office of Extramural Research. A data dictionary will be made that documents and describes the data, including documenting potential limitations. The data dictionary will include details on the source, method of collection, description of the variable, and appropriate references for each data point.

<u>Plans for preservation of the data</u>. Long term retention will be compliant with the NYULH 'Policy on Retention of and Access to Research Data' which mandates retention periods of 6 years after any reporting, publication or presentation, or for the time period specified in the Sponsored Research Agreement.

15 Study Finances

15.1 Funding Source

This study is financed through a grant from the US Department of Health and Human Services, Centers for Disease Control and Prevention. Any referrals to treatment or counseling will be paid for by participants' individual insurance plan, as applicable.

15.2 Costs to the Participant

There are no direct costs to participants related to participation in this study.

15.3 Participant Reimbursements or Payments

Participant compensation at all study sites will follow the same procedures, in accordance with the NYULH Policy on Human Subject Payment.

Participants will receive compensation after completion of baseline visit study procedures as well as after completing follow-up assessments at 1, 3, and 6 months after enrollment. Participants will receive $75 after the baseline visit and will receive $30 after completion of 1 and 3 month follow-up assessments and $50 after completion of the 6 month follow-up assessment. The total compensation for a patient who participates in all RCT assessments is thus $185. At the baseline visit participants will be given a Greenphire ClinCard which will be used loaded with the compensation amounts for follow-up visits upon completion of each visit. Payment for the baseline visit will occur using either cash or the Greenphire ClinCard.

Participants, including patients, Relay peers, and ED staff, in the qualitative interviews will receive compensation of $50 at the completion of the approximately 45-minute interview. For Relay peers and ED staff, the interviews will be conducted outside of regular working hours, and thus participants will be compensated for their time. Relay peers and ED staff will receive a $50 gift card. Patients will be compensated the $50 via their Greenphire ClinCard.

These participant reimbursement amounts are in line with reimbursement amounts that have been used in other studies with similar patient populations and for similar amounts of participant time. They are not expected to constitute undue inducement of patients to participate in the research or to continue beyond a point that they would have otherwise withdrawn. Participants have the right to withdraw from the study without financial penalty. In other words, any reimbursement already distributed to the participant will not be revoked.

Participant reimbursement receipts will document the provision of the reimbursements. A disbursement log in accordance with the NYULH Policy on Human Subject Payments will be used to document distribution of payments with all study participants.

16 Study Administration

16.1 Study Leadership

Overall study leadership is provided by the study MPIs, Dr. Kelly Doran and Dr. Jennifer McNeely. Clinical leads and advisors for the study who will not have a direct role in study conduct or data analysis will not be included on the IRB application.

There is no Steering Committee for this low-risk study. The study DSMB is described in a separate section.

CONFIDENTIAL
This material is the property of the NYU School of Medicine and NYU Langone Health. Do not disclose or use except as authorized in writing by the study sponsor

17 Conflict of Interest Policy

The independence of this study from any actual or perceived influence, such as by the pharmaceutical industry, is critical. Therefore, any actual conflict of interest of persons who have a role in the design, conduct, analysis, publication, or any aspect of this trial will be disclosed and managed. Furthermore, persons who have a perceived conflict of interest will be required to have such conflicts managed in a way that is appropriate to their participation in the trial. The study leadership in conjunction with the CDC has established policies and procedures for all study group members to disclose all conflicts of interest and will establish a mechanism for the management of all reported dualities of interest.

Any investigator who has a conflict of interest with this study (patent ownership, royalties, or financial gain greater than the minimum allowable by their institution, etc.) must have the conflict reviewed by the NYU Langone Conflict of Interest Committee with a Committee-sanctioned conflict management plan that has been reviewed and approved by the study sponsor prior to participation in this study. All NYULH investigators will follow the applicable conflict of interest policies. Investigators from other study sites are expected to follow their own institution’s conflict of interest policies, as well as to report any relevant conflicts of interest to the study MPIs.

This material is the property of the NYU School of Medicine and NYU Langone Health. Do not disclose or use except as authorized in writing by the study sponsor

18 References

1. US Dept. of Health and Human Services (HHS) Office of the Surgeon General. *Facing Addiction in America: The Surgeon General's Report on Alcohol, Drugs, and Health.* Washington, DC2016.
2. Rudd RA, Seth, P., David, F., Scholl, L. Increases in Drug and Opioid-Involved Overdose Deaths - United States, 2010-2015. *MMWR Morbidity and mortality weekly report.* 2016;65(5051):1445-1452.
3. Dowell D, Arias E, Kochanek K, et al. Contribution of Opioid-Involved Poisoning to the Change in Life Expectancy in the United States, 2000-2015. *Jama.* 2017;318(11):1065-1067.
4. U.S. Department of Health and Human Services. The Opioid Epidemic: By the Numbers. 2016; https://www.hhs.gov/sites/default/files/Factsheet-opioids-061516.pdf. Accessed May 21, 2017.
5. Rudd RA, Aleshire, N., Zibbell, J. E., Gladden, R. M. Increases in Drug and Opioid Overdose Deaths--United States, 2000-2014. *MMWR Morbidity and mortality weekly report.* 2016;64(50-51):1378-1382.
6. Hedegaard H, Warner M, Minino AM. Drug Overdose Deaths in the United States, 1999-2016. *NCHS data brief.* 2017(294):1-8.
7. Spencer MR, Warner M, Bastian BA, Trinidad JP, Hedegaard H. *National Vital Statistics Reports: Drug Overdose Deaths Involving Fentanyl, 2011-2016. Vol 68, No 3. March 21, 2019.*
8. Vivolo-Kantor AM, Seth P, Gladden RM, et al. Vital Signs: Trends in Emergency Department Visits for Suspected Opioid Overdoses - United States, July 2016-September 2017. *MMWR Morbidity and mortality weekly report.* 2018;67(9):279-285.
9. Nolan ML, Tuazon E, Blachman-Forshay J, Paone D. *Unintentional Drug Poisoning (Overdose) Deaths in New York City, 2000 to 2017. New York City Department of Health and Mental Hygiene: Epi Data Brief (104).* Sept 2018 2018.
10. New York City Department of Health and Mental Hygiene BoAaDUP, Care, and Treatment. Unpublished data. In:2018.
11. Colon-Berezin C, Nolan ML, Blachman-Forshay J, Paone D. Overdose Deaths Involving Fentanyl and Fentanyl Analogs - New York City, 2000-2017. *MMWR Morbidity and mortality weekly report.* 2019;68(2):37-40.
12. The City of New York Office of the Mayor. Healing NYC: Preventing Overdoses, Saving Lives. 2017; http://www1.nyc.gov/assets/home/downloads/pdf/reports/2017/HealingNYC-Report.pdf.
13. The City of New York. HealingNYC: Mayor and First Lady Announce $22 Million Expansion of City's Plan to Combat Opioid Epidemic. March 19, 2018. https://www1.nyc.gov/office-of-the-mayor/news/143-18/healingnyc-mayor-first-lady-22-million-expansion-city-s-plan-combat-opioid#/0.
14. Coffin PO, Galea S, Ahern J, Leon AC, Vlahov D, Tardiff K. Opiates, cocaine and alcohol combinations in accidental drug overdose deaths in New York City, 1990-98. *Addiction (Abingdon, England).* 2003;98(6):739-747.
15. Darke S, Williamson A, Ross J, Mills KL, Havard A, Teesson M. Patterns of nonfatal heroin overdose over a 3-year period: findings from the Australian treatment outcome study. *Journal of urban health : bulletin of the New York Academy of Medicine.* 2007;84(2):283-291.
16. Darke S, Williamson A, Ross J, Teesson M. Non-fatal heroin overdose, treatment exposure and client characteristics: findings from the Australian treatment outcome study (ATOS). *Drug and alcohol review.* 2005;24(5):425-432.
17. Darke S, Mattick RP, Degenhardt L. The ratio of non-fatal to fatal heroin overdose. *Addiction (Abingdon, England).* 2003;98(8):1169-1171.
18. Darke S, Mills KL, Ross J, Teesson M. Rates and correlates of mortality amongst heroin users: findings from the Australian Treatment Outcome Study (ATOS), 2001-2009. *Drug and alcohol dependence.* 2011;115(3):190-195.
19. Caudarella A, Dong H, Milloy MJ, Kerr T, Wood E, Hayashi K. Non-fatal overdose as a risk factor for subsequent fatal overdose among people who inject drugs. *Drug and alcohol dependence.* 2016;162:51-55.
20. Stoove MA, Dietze PM, Jolley D. Overdose deaths following previous non-fatal heroin overdose: record linkage of ambulance attendance and death registry data. *Drug and alcohol review.* 2009;28(4):347-352.

21. Weiner S, Baker O, Bernson D, Schuur J. One-Year Mortality of Opioid Overdose Victims Who Received Naloxone by Emergency Medical Services. *Annals of emergency medicine.* 2017;70(4):S158.
22. Zadoretzky C, McKnight C, Bramson H, et al. The New York 911 Good Samaritan Law and opioid overdose prevention among people who inject drugs. *World Medical & Health Policy.* 2017;9(3):318-340.
23. Watson DP, Ray B, Robison L, et al. Lay responder naloxone access and Good Samaritan law compliance: postcard survey results from 20 Indiana counties. *Harm reduction journal.* 2018;15(1):18.
24. Pollack HA, Khoshnood K, Blankenship KM, Altice FL. The impact of needle exchange-based health services on emergency department use. *Journal of general internal medicine.* 2002;17(5):341-348.
25. Houry DE, Haegerich TM, Vivolo-Kantor A. Opportunities for Prevention and Intervention of Opioid Overdose in the Emergency Department. *Annals of emergency medicine.* 2018.
26. Rockett IR, Putnam SL, Jia H, Chang CF, Smith GS. Unmet substance abuse treatment need, health services utilization, and cost: a population-based emergency department study. *Annals of emergency medicine.* 2005;45(2):118-127.
27. Dwyer K, Walley AY, Langlois BK, et al. Opioid education and nasal naloxone rescue kits in the emergency department. *The western journal of emergency medicine.* 2015;16(3):381-384.
28. Kestler A, Buxton J, Meckling G, et al. Factors Associated With Participation in an Emergency Department-Based Take-Home Naloxone Program for At-Risk Opioid Users. *Annals of emergency medicine.* 2017;69(3):340-346.
29. Samuels E. Emergency department naloxone distribution: a Rhode Island department of health, recovery community, and emergency department partnership to reduce opioid overdose deaths. *Rhode Island medical journal (2013).* 2014;97(10):38-39.
30. Samuels EA, Dwyer K, Mello MJ, Baird J, Kellogg AR, Bernstein E. Emergency Department-based Opioid Harm Reduction: Moving Physicians From Willing to Doing. *Academic emergency medicine : official journal of the Society for Academic Emergency Medicine.* 2016;23(4):455-465.
31. Martin A, Mitchell A. ED Treatment of Opioid Addiction: An Opportunity to Lead. *Academic Emergency Medicine.* 2017.
32. Saitz R. Absence of a quick fix does not mean ‘do nothing:’time to address drug use in the ED. In: BMJ Publishing Group Ltd and the British Association for Accident & Emergency Medicine; 2018.
33. Scheuermeyer FX, DeWitt C, Christenson J, et al. Safety of a Brief Emergency Department Observation Protocol for Patients With Presumed Fentanyl Overdose. *Annals of emergency medicine.* 2018.
34. Glass JE, Hamilton AM, Powell BJ, Perron BE, Brown RT, Ilgen MA. Specialty substance use disorder services following brief alcohol intervention: a meta-analysis of randomized controlled trials. *Addiction (Abingdon, England).* 2015;110(9):1404-1415.
35. Glass JE, Hamilton AM, Powell BJ, Perron BE, Brown RT, Ilgen MA. Revisiting our review of Screening, Brief Intervention and Referral to Treatment (SBIRT): meta-analytical results still point to no efficacy in increasing the use of substance use disorder services. *Addiction (Abingdon, England).* 2016;111(1):181-183.
36. Saitz R, Cheng DM, Winter M, et al. Chronic care management for dependence on alcohol and other drugs: the AHEAD randomized trial. *Jama.* 2013;310(11):1156-1167.
37. Roy-Byrne P, Bumgardner K, Krupski A, et al. Brief Intervention for Problem Drug Use in Safety-Net Primary Care Settings A Randomized Clinical Trial. *Jama-J Am Med Assoc.* 2014;312(5):492-501.
38. D'Onofrio G, O'Connor PG, Pantalon MV, et al. Emergency department-initiated buprenorphine/naloxone treatment for opioid dependence: a randomized clinical trial. *Jama.* 2015;313(16):1636-1644.
39. Han HR, Kim J, Lee JE, et al. Interventions that increase use of Pap tests among ethnic minority women: a meta-analysis. *Psycho-oncology.* 2011;20(4):341-351.
40. Maxwell AE, Jo AM, Crespi CM, Sudan M, Bastani R. Peer navigation improves diagnostic follow-up after breast cancer screening among Korean American women: results of a randomized trial. *Cancer Causes & Control.* 2010;21(11):1931-1940.

This material is the property of the NYU School of Medicine and NYU Langone Health. Do not disclose or use except as authorized in writing by the study sponsor

41. Grau LE, Griffiths-Kundishora A, Heimer R, et al. Barriers and facilitators of the HIV care continuum in Southern New England for people with drug or alcohol use and living with HIV/AIDS: perspectives of HIV surveillance experts and service providers. *Addiction science & clinical practice.* 2017;12(1):24.
42. Steward WT, Sumitani J, Moran ME, et al. Engaging HIV-positive clients in care: acceptability and mechanisms of action of a peer navigation program in South Africa. *AIDS care.* 2018;30(3):330-337.
43. Flay BR, Snyder F, Petraitis J. The theory of triadic influence. In: DiClimente RJ, Kegler MC, Crosby RA, eds. *Emerging theories in health promotion practice and research.* New York: Jossey-Bass; 2009:451-510.
44. Nowak A, Szamrej J, Latané B. From private attitude to public opinion: A dynamic theory of social impact. *Psychological Review.* 1990;97(3):362.
45. D'Onofrio G, McCormack RP, Hawk K. Emergency Departments - A 24/7/365 Option for Combating the Opioid Crisis. *The New England journal of medicine.* 2018;379(26):2487-2490.
46. D'Onofrio G, Degutis LC. Integrating Project ASSERT: a screening, intervention, and referral to treatment program for unhealthy alcohol and drug use into an urban emergency department. *Academic emergency medicine : official journal of the Society for Academic Emergency Medicine.* 2010;17(8):903-911.
47. Waye KM, Goyer J, Dettor D, et al. Implementing peer recovery services for overdose prevention in Rhode Island: An examination of two outreach-based approaches. *Addict Behav.* 2019;89:85-91.
48. Samuels EA, Bernstein SL, Marshall BDL, Krieger M, Baird J, Mello MJ. Peer navigation and take-home naloxone for opioid overdose emergency department patients: Preliminary patient outcomes. *Journal of substance abuse treatment.* 2018;94:29-34.
49. Bohnert AS, Bonar EE, Cunningham R, et al. A pilot randomized clinical trial of an intervention to reduce overdose risk behaviors among emergency department patients at risk for prescription opioid overdose. *Drug & Alcohol Dependence.* 2016;163:40-47.
50. Banta-Green CJ, Coffin PO, Merrill JO, et al. Impacts of an opioid overdose prevention intervention delivered subsequent to acute care. *Injury prevention : journal of the International Society for Child and Adolescent Injury Prevention.* 2018.
51. D'Onofrio G, Chawarski MC, O'Connor PG, et al. Emergency Department-Initiated Buprenorphine for Opioid Dependence with Continuation in Primary Care: Outcomes During and After Intervention. *Journal of general internal medicine.* 2017;32(6):660-666.
52. Coffin PO, Santos GM, Matheson T, et al. Behavioral intervention to reduce opioid overdose among high-risk persons with opioid use disorder: A pilot randomized controlled trial. *PloS one.* 2017;12(10):e0183354.
53. Gwadz M, Rotheram-Borus MJ. Tracking high-risk adolescents longitudinally. *AIDS Educ Prev.* 1992;Suppl:69-82.
54. Gwadz MV, Cylar K, Leonard NR, et al. An exploratory behavioral intervention trial to improve rates of screening for AIDS clinical trials among racial/ethnic minority and female persons living with HIV/AIDS. *AIDS Behav.* 2010;14(3):639-648.
55. Rotheram-Borus MJ, Lee M, Leonard N, et al. Four-year behavioral outcomes of an intervention for parents living with HIV and their adolescent children. *Aids.* 2003;17(8):1217-1225.
56. Martino S, Ball SA, Gallon SL, et al. *Motivational interviewing assessment: Supervisory tools for enhancing proficiency.* Salem, OR: Northwest Frontier Addiction technology Transfer Center, Oregon Health and Science University;2006.
57. Jeste DV, Palmer BW, Appelbaum PS, et al. A new brief instrument for assessing decisional capacity for clinical research. *Arch Gen Psychiatry.* 2007;64(8):966-974.
58. Maeng DD, Han JJ, Fitzpatrick MH, Boscarino JA. Patterns of health care utilization and cost before and after opioid overdose: findings from 10-year longitudinal health plan claims data. *Substance abuse and rehabilitation.* 2017;8:57-67.

This material is the property of the NYU School of Medicine and NYU Langone Health. Do not disclose or use except as authorized in writing by the study sponsor

19 Attachments

These documents are relevant to the protocol, but they are not considered part of the protocol. They are stored and modified separately. As such, modifications to these documents do not require protocol amendments, with the exception of key information sheets and consent forms, which require IRB approval for any changes.

The following attachments are included:

Attachment A.	Schedule of Events
Attachment B.	Consent Form – Main Study
Attachment C.	Key Information Sheet – Main Study
Attachment D.	Verbal Consent Form – Qualitative Interview Patients
Attachment E.	Verbal Consent Form – Qualitative Interview Providers
Attachment F.	UBACC for RCT
Attachment G.	Baseline Screening and Survey (Questionnaire, Information Form, Exit Survey)
Attachment H.	Locator Form
Attachment I.	Baseline SDC Group Handouts
Attachment J.	Follow-up Questionnaires (1, 3, 6-month)
Attachment K.	Qualitative Interview Guide, ED Providers
Attachment L.	Qualitative Interview Guide, Patients
Attachment M.	Qualitative Interview Guide, Relay Peers
Attachment N.	Interim Contact Form
Attachment O.	Study Contact Card and Sticker
Attachment P.	Administrative Data Abstraction Tool
Attachment Q.	NYU Maven Data Entry Elements
Attachment R.	Prisoner Appendix
Attachment S.	Relay Scheduling Tool
Attachment T.	NYU Relay Evaluation DUA with DOHMH
Attachment U.	Appointment Reminder Mailing
Attachment V.	Contacts’ Card Letter Insert
Attachment W.	Contacts’ Card
Attachment X.	Happy Holidays Card
Attachment Y.	Thank you Card

CONFIDENTIAL
This material is the property of the NYU School of Medicine and NYU Langone Health. Do not disclose or use except as authorized in writing by the study sponsor

Attachment A

Schedule of Events

Activity	Baseline	1 Month Visit	3 Month Visit	Interim Contacts	6 Month Visit	Qualitative Interviews	12-Month Outcome Abstraction
**Screening**							
ED provider calls Relay hotline	X						
Assignment of RA	X						
Screening for eligibility	X						
Informed consent process	X					X	
**Primary Study Procedures**							
Randomization	X						
Participant questionnaire	X	X	X		X		
Participant information form	X						
Locator form	X						
Relay intervention or SDC*	X						
Exit survey	X						
Semi-structured interview						X	
Administrative data extraction							X
**Additional Participant Contacts**							
Mail “Thank you” card (within week following baseline visit)				X			
Mail “Holiday” card (if applicable)				X			
Mail “Appointment reminder” cards (if applicable)				X			
Confirm locator form information		X	X	X	X		
Confirm next study appointment	X	X	X	X	X		
**Additional Study Procedures**							
Give study contact card	X						
Reimbursement and Greenphire ClinCard FAQ (if applicable)	X	X	X		X	X	

CONFIDENTIAL
This material is the property of the NYU School of Medicine and Langone Health. Do not disclose or use except as authorized in writing by the study sponsor

2193
