# Supplementary material for: Peer Navigator Intervention and Opioid-Related Adverse Events for Emergency Department Patients: A Randomized Clinical Trial
Source: JAMA Netw Open. 2026 Feb 6;9(2):e2555903. doi: 10.1001/jamanetworkopen.2025.55903 (PMC12881982; doi:10.1001/jamanetworkopen.2025.55903)
Supplement: Supplement 2. — eMethods. Additional Details on Relay Intervention and Statistical Analyses eTable. List of Diagnosis Codes for Substance Use-Related ED Visits eFigure 1. Summary of Outcome Effects for Relay vs. Site-Directed Care Arms (Intent-to- Treat Analyses) eFigure 2. Visualization of Primary Outcome (Opioid-Related Adverse Events) for Relay and SDC Arms (Intent-to-Treat Analyses) eFigure 3. Survival Plot for Overdose Mortality Comparing Relay and SDC Arm Participants (Intention-to-Treat Analysis) eFigure 4. Survival Plot for Opioid Overdose Mortality Comparing Relay and SDC Arm Participants (Intent-to-Treat Analysis) eFigure 5. Survival Plot for Time to Next Opioid-Involved Overdose Comparing Relay and SDC Arm Participants (Intent-to-Treat Analysis) eFigure 6. Survival Plot for Time to Next Substance Use-related ED Visit Comparing Relay and SDC Arm Participants (Intent-to-Treat Analysis) [file jamanetwopen-e2555903-s002.pdf]

Supplemental Online Content

Doran KM, Welch AE, Kepler KL, et al. Peer navigator intervention and opioid-related adverse events for emergency department patients: a randomized clinical trial. *JAMA Netw Open*. 2026;9(2):e2555903. doi:10.1001/jamanetworkopen.2025.55903

eMethods. Additional Details on Relay Intervention and Statistical Analyses

eTable. List of Diagnosis Codes for Substance Use-Related ED Visits

eFigure 1. Summary of Outcome Effects for Relay vs. Site-Directed Care Arms (Intention-to-Treat Analyses)

eFigure 2. Visualization of Primary Outcome (Opioid-Related Adverse Events) for Relay and SDC Arms (Intent-to-Treat Analyses)

eFigure 3. Survival Plot for Overdose Mortality Comparing Relay and SDC Arm Participants (Intent-to-Treat Analysis)

eFigure 4. Survival Plot for Opioid Overdose Mortality Comparing Relay and SDC Arm Participants (Intent-to-Treat Analysis)

eFigure 5. Survival Plot for Time to Next Opioid-Involved Overdose Comparing Relay and SDC Arm Participants (Intent-to-Treat Analysis)

eFigure 6. Survival Plot for Time to Next Substance Use-related ED Visit Comparing Relay and SDC Arm Participants (Intent-to-Treat Analysis)

This supplemental material has been provided by the authors to give readers additional information about their work.

© 2026 Doran KM et al. *JAMA Network Open.*

eMethods.

Relay intervention details. Relay is operated by the NYC Health Department and delivered by trained peer navigators (“Wellness Advocates” [WAs]). Relay serves ED patients presenting after a nonfatal opioid-involved overdose and provides overdose prevention education, naloxone, support, and linkage to care to opioid overdose survivors for 90 days after an overdose event. ED providers at participating hospitals call a centralized “Relay hotline” to dispatch a WA to come to the ED (with an expected arrival time within 1 hour). The EDs have protocols specifying who should call Relay and when the call should be made. ED providers receive initial and ongoing education and encouragement to call the Relay hotline for all patients with an opioid-involved overdose, but ultimately calling the hotline is at the provider’s discretion. ED providers are not required to obtain explicit patient consent prior to calling the Relay hotline. Relay has been described in more detail in prior publications, including: https://doi.org/10.2105/AJPH.2019.305202.

Relay Wellness Advocate (WA) Qualifications. The hiring requirements for Wellness Advocates state that "A Wellness Advocate possesses first-hand lived experience in substance use, either directly or through a friend or family member." The hiring requirements also include either a recovery coach certification, CRPA/CRPA-P, CARC, CASAC/CASAC-T, or 2 years of commensurate peer experience. The NYC Health Department provides additional training on Relay's procedures and framework, as well as ongoing skills trainings and refreshers including motivational interviewing, recovery coach peer trainings (CRPA and CCAR), and a variety of substance use trainings. WAs are assigned to cover specific hospitals, often based on where they live (to ensure quick response times); thus there is an increased likelihood of WA demographic and language concordance with the patients given that they are coming from the same local community. For baseline ED visits in which the WA does not speak the patient’s language they have access to a Language Line telephone interpretation service. Additionally, for follow-up visits bilingual WAs are generally assigned to Spanish-speaking patients to ensure language concordance.

Randomization. CMC set up the randomization. A single block had a 0.6 probability of being two allocations and a 0.4 probability of being four allocations. The allocations to SDC and Relay conditions were randomly shuffled within each block using the sample function of the R statistical computing environment. A total of 600 blocks were generated in this way and combined into a comma-delimited allocation table which was stratified by study site (n=150 permuted blocks in each of four sites). The allocation table was uploaded to the REDCap randomization module. Research assistants and coordinators within each emergency department were blind to the contents of the allocation table, and used the REDCap “randomize” button to request an allocated condition from the table.

Administrative data linkage and analysis. Administrative data were obtained from the two NYC Regional Health Information Organization (RHIOs), Healthix and the Bronx Regional Health Information Organization (RHIO), that together include data for visits to nearly all hospitals in NYC, aside from Veterans Affairs hospitals. RHIO data include dates and diagnostic codes for ED visits. The study team provided participant identifiers (collected via a combination of patient self-report and observation of patient hospital ID bracelet at the baseline visit) for all trial participants to Bronx RHIO and Healthix for data linkage and received back data on visits and associated diagnostic codes for participants for a time period spanning one year or more post-enrollment (i.e., covering all outcome time periods). Identifiers included patient first and last name, date of birth, medical record number, and site where the baseline ED visit occurred. Additional identifiers were provided when available including social security number, gender, address, and telephone number. Eligible visits occurred in the period from one year before to one year after the baseline ED visit. Each RHIO determined its own linkage / matching procedure, which was not conducted directly by the study team and generally included exact (rather than fuzzy) matching using available identifying information. The RHIOs and study team worked together to attempt to resolve any issues of unsuccessful matching (e.g., participants not found in the data) by correcting or collecting additional identifying information as possible.

Mortality data were obtained from the NYC Health Department’s Office of Vital Statistics. The NYC Health Department matched all RCT participants to all-cause mortality data and death certificates that the NYC

© 2026 Doran KM et al. *JAMA Network Open.*

Health Department's Bureau of Vital Statistics maintains and reports, based on exact or near matches of identifying data, including first and last name, date of birth, and address. All potential matches were manually reviewed. The NYC Health Department had the complete list of all RCT participants for this matching.

Additional detail, including analytic code, is available upon request to the authors.

Missing data. For administrative data, visits to sites which were not emergency departments were excluded from results received from Bronx RHIO and Healthix. Eligible ED visits were found for 219 participants (89%). Thus, we were unsuccessful in finding at least one ED visit (the expected baseline visit) in administrative data for 28 participants (11.3%). When a visit for the same day at the same ED site appeared in both Bronx RHIO and Healthix data, it was counted as a single visit. We acknowledge this process likely results in an undercount (due to incomplete linkage) of all ED visits experienced by participants during the study period. Mortality data is considered complete by the NYC Health Department Office of Vital Statistics, for all deaths occurring within NYC. For questionnaire data, details on missing data appear in the table below.

eMethods Table 1. Missing data for follow-up questionnaires

	Condition		
	SDC	Relay	Total
Follow-Up Questionnaires Completed			
None Completed	36 (30%)	34 (27%)	70 (28%)
1M Only	4 (3.3%)	7 (5.6%)	11 (4.5%)
3M Only	5 (4.1%)	7 (5.6%)	12 (4.9%)
6M Only	7 (5.7%)	6 (4.8%)	13 (5.3%)
1M & 3M	8 (6.6%)	7 (5.6%)	15 (6.1%)
1M & 6M	3 (2.5%)	3 (2.4%)	6 (2.4%)
3M & 6M	12 (9.8%)	12 (9.6%)	24 (9.7%)
All Completed	47 (39%)	49 (39%)	96 (39%)
Total	122 (100%)	125 (100%)	247 (100%)

Multiple imputation methods. The default number of cycles was used (n=5). As part of building the overall primary outcome, a total of 17 variables with some missing data were included in multiple imputation. ED site and study arm were included in all imputation models. Additionally, variables with at least 50% usable cases and an absolute correlation of at least r = .10 between the potential predictor and the variable to be imputed or between the potential predictor and an indicator of missing data in the variable to be imputed. The table below shows the percentage of data missing and the variables used in the imputation model for each of these 17 variables.

After imputing multiple datasets, each was augmented to construct the primary outcome from constituent parts. Within each imputed dataset, based on the observed relationship between self-reported opioid-involved overdose not leading to an ED visit and all substance use related ED visits from administrative data in the period covered by self-report (baseline + any follow-up), we predicted opioid-involved overdoses not leading to an ED visit in the period not captured by self-report, using data on substance use related ED visits in that period. This prediction included an offset for days covered by self-report. The primary outcome added together the following: 1) substance use related ED visits from administrative data; 2) opioid-involved overdoses not leading to an ED visit in the portion of the follow-up period covered by self-report; 3) opioid-involved overdoses not leading to an ED visit in the portion of the follow-up period *not* covered by self-report; and 4) any fatal overdose in the year following baseline.

© 2026 Doran KM et al. *JAMA Network Open.*

eMethods Table 2. Multiple imputation details

	Imputed Variable	Participants with Missing Data	Variables Used to Impute
1	Self-reported opioid involved overdoses not leading to ED visit in the follow-up period	n=70; 28.3%	ED Site + Study Arm + Self-Report Follow-Up Days + Self-reported opioid involved overdoses not leading to ED visit in the baseline period + Substance use related ED visits in the follow-up period that overlapped with self-report + NA + Substance use related ED visits in the baseline period + All ED visits in the follow-up period that overlapped with self-report + All ED visits in the follow-up period that did NOT overlap with self-report + All ED visits in the baseline period + WHO ASSIST Prescription Opioid Risk Score at baseline + MOUD at baseline
2	Substance use related ED visits in the baseline period	n=28; 11.3%	ED Site + Study Arm + Self-Report Follow-Up Days + Self-reported opioid involved overdoses not leading to ED visit in the follow-up period + Substance use related ED visits in the follow-up period that overlapped with self-report + NA + All ED visits in the follow-up period that overlapped with self-report + All ED visits in the follow-up period that did NOT overlap with self-report + WHO ASSIST Heroin Risk Score at baseline + MOUD at baseline + MOUD at 3M + MOUD at 6M + Self-reported opioid-involved overdose at 3M + Self-reported opioid-involved overdose at 6M + All self-reported opioid involved overdose not leading to ED visit (baseline + any follow-up)
3	All ED visits in the baseline period	n=28; 11.3%	ED Site + Study Arm + Self-Report Follow-Up Days + Substance use related ED visits in the follow-up period that overlapped with self-report + NA + All ED visits in the follow-up period that overlapped with self-report + All ED visits in the follow-up period that did NOT overlap with self-report + WHO ASSIST Heroin Risk Score at baseline + MOUD at baseline + MOUD at 3M + MOUD at 6M + Bohnert opioid risk behavior score at baseline + Bohnert opioid risk behavior score at 3M + Self-reported opioid-involved overdose at 3M + Self-reported opioid-involved overdose at 6M + All self-reported opioid involved overdose not leading to ED visit (baseline + any follow-up)
4	WHO ASSIST Heroin Risk Score at baseline	n=18; 7.3%	ED Site + Study Arm + Fatal Overdose within 365 Days + Self-reported opioid involved overdoses not leading to ED visit in the baseline period + NA + Substance use related ED visits in the baseline period + All ED visits in the baseline period + WHO ASSIST Prescription Opioid Risk Score at baseline + MOUD at baseline + MOUD at 3M + MOUD at 6M + Bohnert opioid risk behavior score at baseline + Bohnert opioid risk behavior score at 3M + Bohnert opioid risk behavior score at 6M + Self-reported opioid-involved overdose at baseline + Self-reported opioid-involved overdose at 3M + Self-reported opioid-involved overdose at 6M + All self-reported opioid involved overdose not leading to ED visit (baseline + any follow-up)

© 2026 Doran KM et al. *JAMA Network Open.*

5	WHO ASSIST Prescription Opioid Risk Score at baseline	n=8; 3.2%	ED Site + Study Arm + Self-Report Follow-Up Days + Self-reported opioid involved overdoses not leading to ED visit in the baseline period + MOUD at baseline + Bohnert opioid risk behavior score at baseline + Bohnert opioid risk behavior score at 6M + Self-reported opioid-involved overdose at 6M + All self-reported opioid involved overdose not leading to ED visit (baseline + any follow-up)
6	MOUD at baseline	n=12; 4.9%	ED Site + Study Arm + Self-Report Follow-Up Days + Self-reported opioid involved overdoses not leading to ED visit in the baseline period + WHO ASSIST Heroin Risk Score at baseline + WHO ASSIST Prescription Opioid Risk Score at baseline + Bohnert opioid risk behavior score at baseline
7	MOUD at 1M	n=120; 48.6%	ED Site + Study Arm + Self-Report Follow-Up Days + Substance use related ED visits in the follow-up period that overlapped with self-report + NA + Substance use related ED visits in the baseline period + All ED visits in the follow-up period that overlapped with self-report + All ED visits in the follow-up period that did NOT overlap with self-report + All ED visits in the baseline period + WHO ASSIST Heroin Risk Score at baseline + WHO ASSIST Prescription Opioid Risk Score at baseline + MOUD at baseline + Bohnert opioid risk behavior score at baseline
8	MOUD at 3M	n=101; 40.9%	ED Site + Study Arm + Self-Report Follow-Up Days + Substance use related ED visits in the follow-up period that overlapped with self-report + NA + Substance use related ED visits in the baseline period + All ED visits in the follow-up period that overlapped with self-report + All ED visits in the follow-up period that did NOT overlap with self-report + All ED visits in the baseline period + WHO ASSIST Heroin Risk Score at baseline + WHO ASSIST Prescription Opioid Risk Score at baseline + MOUD at baseline + Bohnert opioid risk behavior score at baseline + Self-reported opioid-involved overdose at baseline
9	MOUD at 6M	n=108; 43.7%	ED Site + Study Arm + Self-Report Follow-Up Days + Fatal Overdose within 365 Days + Substance use related ED visits in the follow-up period that overlapped with self-report + NA + Substance use related ED visits in the baseline period + All ED visits in the follow-up period that overlapped with self-report + All ED visits in the follow-up period that did NOT overlap with self-report + All ED visits in the baseline period + WHO ASSIST Heroin Risk Score at baseline + WHO ASSIST Prescription Opioid Risk Score at baseline + MOUD at baseline + Bohnert opioid risk behavior score at baseline

© 2026 Doran KM et al. *JAMA Network Open.*

10	Bohnert opioid risk behavior score at 1M	n=119; 48.2%	ED Site + Study Arm + Self-Report Follow-Up Days + Self-reported opioid involved overdoses not leading to ED visit in the baseline period + Substance use related ED visits in the follow-up period that overlapped with self-report + NA + Substance use related ED visits in the baseline period + All ED visits in the follow-up period that overlapped with self-report + All ED visits in the follow-up period that did NOT overlap with self-report + All ED visits in the baseline period + WHO ASSIST Heroin Risk Score at baseline + WHO ASSIST Prescription Opioid Risk Score at baseline + MOUD at baseline + Bohnert opioid risk behavior score at baseline + Self-reported opioid-involved overdose at baseline
11	Bohnert opioid risk behavior score at 3M	n=100; 40.5%	ED Site + Study Arm + Self-Report Follow-Up Days + Substance use related ED visits in the follow-up period that overlapped with self-report + NA + Substance use related ED visits in the baseline period + All ED visits in the follow-up period that overlapped with self-report + All ED visits in the follow-up period that did NOT overlap with self-report + All ED visits in the baseline period + WHO ASSIST Heroin Risk Score at baseline + WHO ASSIST Prescription Opioid Risk Score at baseline + MOUD at baseline + Bohnert opioid risk behavior score at baseline + Self-reported opioid-involved overdose at baseline
12	Bohnert opioid risk behavior score at 6M	n=108; 43.7%	ED Site + Study Arm + Self-Report Follow-Up Days + Fatal Overdose within 365 Days + Substance use related ED visits in the follow-up period that overlapped with self-report + NA + Substance use related ED visits in the baseline period + All ED visits in the follow-up period that overlapped with self-report + All ED visits in the follow-up period that did NOT overlap with self-report + All ED visits in the baseline period + WHO ASSIST Heroin Risk Score at baseline + WHO ASSIST Prescription Opioid Risk Score at baseline + MOUD at baseline + Bohnert opioid risk behavior score at baseline
13	Self-reported opioid-involved overdose at baseline	n=9; 3.6%	ED Site + Study Arm + Substance use related ED visits in the follow-up period that overlapped with self-report + All ED visits in the follow-up period that overlapped with self-report + WHO ASSIST Heroin Risk Score at baseline + WHO ASSIST Prescription Opioid Risk Score at baseline + MOUD at 1M + MOUD at 3M + Bohnert opioid risk behavior score at baseline + Bohnert opioid risk behavior score at 1M + Bohnert opioid risk behavior score at 3M + Self-reported opioid-involved overdose at 1M + Self-reported opioid-involved overdose at 3M

© 2026 Doran KM et al. *JAMA Network Open.*

14	Self-reported opioid-involved overdose at 1M	n=119; 48.2%	ED Site + Study Arm + Self-Report Follow-Up Days + Substance use related ED visits in the follow-up period that overlapped with self-report + NA + Substance use related ED visits in the baseline period + All ED visits in the follow-up period that overlapped with self-report + All ED visits in the follow-up period that did NOT overlap with self-report + All ED visits in the baseline period + WHO ASSIST Heroin Risk Score at baseline + WHO ASSIST Prescription Opioid Risk Score at baseline + MOUD at baseline + Bohnert opioid risk behavior score at baseline + Self-reported opioid-involved overdose at baseline
15	Self-reported opioid-involved overdose at 3M	n=100; 40.5%	ED Site + Study Arm + Self-Report Follow-Up Days + Self-reported opioid involved overdoses not leading to ED visit in the baseline period + Substance use related ED visits in the follow-up period that overlapped with self-report + NA + Substance use related ED visits in the baseline period + All ED visits in the follow-up period that overlapped with self-report + All ED visits in the follow-up period that did NOT overlap with self-report + All ED visits in the baseline period + WHO ASSIST Heroin Risk Score at baseline + WHO ASSIST Prescription Opioid Risk Score at baseline + MOUD at baseline + Bohnert opioid risk behavior score at baseline + Self-reported opioid-involved overdose at baseline
16	Self-reported opioid-involved overdose at 6M	n=108; 43.7%	ED Site + Study Arm + Self-Report Follow-Up Days + Fatal Overdose within 365 Days + Self-reported opioid involved overdoses not leading to ED visit in the baseline period + Substance use related ED visits in the follow-up period that overlapped with self-report + NA + Substance use related ED visits in the baseline period + All ED visits in the follow-up period that overlapped with self-report + All ED visits in the follow-up period that did NOT overlap with self-report + All ED visits in the baseline period + WHO ASSIST Heroin Risk Score at baseline + WHO ASSIST Prescription Opioid Risk Score at baseline + MOUD at baseline + Bohnert opioid risk behavior score at baseline
17	All self-reported opioid involved overdose not leading to ED visit (baseline + any follow-up)	n=70; 28.3%	ED Site + Study Arm + Self-Report Follow-Up Days + Fatal Overdose within 365 Days + Self-reported opioid involved overdoses not leading to ED visit in the baseline period + Substance use related ED visits in the follow-up period that overlapped with self-report + NA + Substance use related ED visits in the baseline period + All ED visits in the follow-up period that overlapped with self-report + All ED visits in the follow-up period that did NOT overlap with self-report + All ED visits in the baseline period + WHO ASSIST Heroin Risk Score at baseline + WHO ASSIST Prescription Opioid Risk Score at baseline + MOUD at baseline + Bohnert opioid risk behavior score at baseline

© 2026 Doran KM et al. *JAMA Network Open.*

eTable 1. List of Diagnosis Codes for Substance Use-Related ED Visits

**Opioid Overdose**							
T40.0X1A	T40.0X4A	T40.1X3A	T40.2X2A	T40.3X1A	T40.3X4A	T40.413A	T40.422A
T40.0X1D	T40.0X4D	T40.1X3D	T40.2X2D	T40.3X1D	T40.3X4D	T40.413D	T40.422D
T40.0X1S	T40.0X4S	T40.1X3S	T40.2X2S	T40.3X1S	T40.3X4S	T40.413S	T40.422S
T40.0X2A	T40.1X1A	T40.1X4A	T40.2X3A	T40.3X2A	T40.411A	T40.414A	T40.423A
T40.0X2D	T40.1X1D	T40.1X4D	T40.2X3D	T40.3X2D	T40.411D	T40.414D	T40.423D
T40.0X2S	T40.1X1S	T40.1X4S	T40.2X3S	T40.3X2S	T40.411S	T40.414S	T40.423S
T40.0X3A	T40.1X2A	T40.2X1A	T40.2X4A	T40.3X3A	T40.412A	T40.421A	T40.424A
T40.0X3D	T40.1X2D	T40.2X1D	T40.2X4D	T40.3X3D	T40.412D	T40.421D	T40.424D
T40.0X3S	T40.1X2S	T40.2X1S	T40.2X4S	T40.3X3S	T40.412S	T40.421S	T40.424S
**Opioid, Not Overdose**							
F11.10	F11.150	F11.21	F11.251	F11.920	F11.959	T40.0X5S	T40.3X5D
F11.11	F11.151	F11.220	F11.259	F11.921	F11.981	T40.1X5A	T40.3X5S
F11.120	F11.159	F11.221	F11.281	F11.922	F11.982	T40.1X5D	T40.415A
F11.121	F11.181	F11.222	F11.282	F11.929	F11.988	T40.1X5S	T40.415D
F11.122	F11.182	F11.229	F11.288	F11.93	F11.99	T40.2X5A	T40.415S
F11.129	F11.188	F11.23	F11.29	F11.94	R78.1	T40.2X5D	T40.425A
F11.13	F11.19	F11.24	F11.90	F11.950	T40.0X5A	T40.2X5S	T40.425D
F11.14	F11.20	F11.250	F11.91	F11.951	T40.0X5D	T40.3X5A	T40.425S
**Overdose, Not Opioid**							
T40.491A	T40.5X2A	T40.693A	T40.724A	T40.901A	T42.3X2A	T43.603A	T43.654A
T40.491D	T40.5X2D	T40.693D	T40.724D	T40.901D	T42.3X2D	T43.603D	T43.654D
T40.491S	T40.5X2S	T40.693S	T40.724S	T40.901S	T42.3X2S	T43.603S	T43.654S
T40.492A	T40.5X3A	T40.694A	T40.7X1A	T40.902A	T42.3X3A	T43.604A	T50.901A
T40.492D	T40.5X3D	T40.694D	T40.7X1D	T40.902D	T42.3X3D	T43.604D	T50.901S
T40.492S	T40.5X3S	T40.694S	T40.7X1S	T40.902S	T42.3X3S	T43.604S	T50.902A
T40.493A	T40.5X4A	T40.711A	T40.7X2A	T40.903A	T42.3X4A	T43.641A	T50.904A
T40.493D	T40.5X4D	T40.711D	T40.7X2D	T40.903D	T42.3X4D	T43.641D	T50.904S
T40.493S	T40.5X4S	T40.711S	T40.7X2S	T40.903S	T42.3X4S	T43.641S	T50.991A
T40.494A	T40.601A	T40.712A	T40.7X3A	T40.904A	T42.4X1A	T43.642A	T51.0X1A
T40.494D	T40.601D	T40.712D	T40.7X3D	T40.904D	T42.4X1D	T43.642D	T51.0X1D
T40.494S	T40.601S	T40.712S	T40.7X3S	T40.904S	T42.4X1S	T43.642S	T51.0X1S
T40.4X1A	T40.602A	T40.713A	T40.7X4A	T40.991A	T42.4X2A	T43.643A	T51.0X2A
T40.4X1D	T40.602D	T40.713D	T40.7X4D	T40.991D	T42.4X2D	T43.643D	T51.0X2D
T40.4X1S	T40.602S	T40.713S	T40.7X4S	T40.991S	T42.4X2S	T43.643S	T51.0X2S
T40.4X2A	T40.603A	T40.714A	T40.8X1A	T40.992A	T42.4X3A	T43.644A	T51.0X3A
T40.4X2D	T40.603D	T40.714D	T40.8X1D	T40.992D	T42.4X3D	T43.644D	T51.0X3D
T40.4X2S	T40.603S	T40.714S	T40.8X1S	T40.992S	T42.4X3S	T43.644S	T51.0X3S
T40.4X3A	T40.604A	T40.721A	T40.8X2A	T40.993A	T42.4X4A	T43.651A	T51.0X4A

© 2026 Doran KM et al. *JAMA Network Open.*

T40.4X3D	T40.604D	T40.721D	T40.8X2D	T40.993D	T42.4X4D	T43.651D	T51.0X4D
T40.4X3S	T40.604S	T40.721S	T40.8X2S	T40.993S	T42.4X4S	T43.651S	T51.0X4S
T40.4X4A	T40.691A	T40.722A	T40.8X3A	T40.994A	T43.601A	T43.652A	T51.91XA
T40.4X4D	T40.691D	T40.722D	T40.8X3D	T40.994D	T43.601D	T43.652D	
T40.4X4S	T40.691S	T40.722S	T40.8X3S	T40.994S	T43.601S	T43.652S	
T40.5X1A	T40.692A	T40.723A	T40.8X4A	T42.3X1A	T43.602A	T43.653A	
T40.5X1D	T40.692D	T40.723D	T40.8X4D	T42.3X1D	T43.602D	T43.653D	
T40.5X1S	T40.692S	T40.723S	T40.8X4S	T42.3X1S	T43.602S	T43.653S	
**Other Substance Use**							
F10.10	F12.10	F13.21	F14.221	F15.282	F16.99	F19.182	R78.0
F10.11	F12.11	F13.220	F14.222	F15.288	F18.10	F19.188	R78.2
F10.120	F12.120	F13.221	F14.229	F15.29	F18.11	F19.19	R78.3
F10.121	F12.121	F13.229	F14.23	F15.90	F18.120	F19.20	R78.4
F10.129	F12.122	F13.230	F14.24	F15.91	F18.121	F19.21	T40.495A
F10.130	F12.129	F13.231	F14.250	F15.920	F18.129	F19.220	T40.495D
F10.131	F12.13	F13.232	F14.251	F15.921	F18.14	F19.221	T40.495S
F10.132	F12.150	F13.239	F14.259	F15.922	F18.150	F19.222	T40.4X5A
F10.139	F12.151	F13.24	F14.280	F15.929	F18.151	F19.229	T40.4X5D
F10.14	F12.159	F13.250	F14.281	F15.93	F18.159	F19.230	T40.4X5S
F10.150	F12.180	F13.251	F14.282	F15.94	F18.17	F19.231	T40.5X5A
F10.151	F12.188	F13.259	F14.288	F15.950	F18.180	F19.232	T40.5X5D
F10.159	F12.19	F13.26	F14.29	F15.951	F18.188	F19.239	T40.5X5S
F10.180	F12.20	F13.27	F14.90	F15.959	F18.19	F19.24	T40.605A
F10.181	F12.21	F13.280	F14.91	F15.980	F18.20	F19.250	T40.605D
F10.182	F12.220	F13.281	F14.920	F15.981	F18.21	F19.251	T40.605S
F10.188	F12.221	F13.282	F14.921	F15.982	F18.220	F19.259	T40.695A
F10.19	F12.222	F13.288	F14.922	F15.988	F18.221	F19.26	T40.695D
F10.20	F12.229	F13.29	F14.929	F15.99	F18.229	F19.27	T40.695S
F10.21	F12.23	F13.90	F14.93	F16.10	F18.24	F19.280	T40.715A
F10.220	F12.250	F13.91	F14.94	F16.11	F18.250	F19.281	T40.715D
F10.221	F12.251	F13.920	F14.950	F16.120	F18.251	F19.282	T40.715S
F10.229	F12.259	F13.921	F14.951	F16.121	F18.259	F19.288	T40.725A
F10.230	F12.280	F13.929	F14.959	F16.122	F18.27	F19.29	T40.725D
F10.231	F12.288	F13.930	F14.980	F16.129	F18.280	F19.90	T40.725S
F10.232	F12.29	F13.931	F14.981	F16.14	F18.288	F19.91	T40.7X5A
F10.239	F12.90	F13.932	F14.982	F16.150	F18.29	F19.920	T40.7X5D
F10.24	F12.91	F13.939	F14.988	F16.151	F18.90	F19.921	T40.7X5S
F10.250	F12.920	F13.94	F14.99	F16.159	F18.91	F19.922	T40.8X5A
F10.251	F12.921	F13.950	F15.10	F16.180	F18.920	F19.929	T40.8X5D
F10.259	F12.922	F13.951	F15.11	F16.183	F18.921	F19.930	T40.8X5S
F10.26	F12.929	F13.959	F15.120	F16.188	F18.929	F19.931	T40.905A

© 2026 Doran KM et al. *JAMA Network Open.*

F10.27	F12.93	F13.96	F15.121	F16.19	F18.94	F19.932	T40.905D
F10.280	F12.950	F13.97	F15.122	F16.20	F18.950	F19.939	T40.905S
F10.281	F12.951	F13.980	F15.129	F16.21	F18.951	F19.94	T40.995A
F10.282	F12.959	F13.981	F15.13	F16.220	F18.959	F19.950	T40.995D
F10.288	F12.980	F13.982	F15.14	F16.221	F18.97	F19.951	T40.995S
F10.29	F12.988	F13.988	F15.150	F16.229	F18.980	F19.959	T42.3X5A
F10.90	F12.99	F13.99	F15.151	F16.24	F18.988	F19.96	T42.3X5D
F10.91	F13.10	F14.10	F15.159	F16.250	F18.99	F19.97	T42.3X5S
F10.920	F13.11	F14.11	F15.180	F16.251	F19.10	F19.980	T42.4X5A
F10.921	F13.120	F14.120	F15.181	F16.259	F19.11	F19.981	T42.4X5D
F10.929	F13.121	F14.121	F15.182	F16.280	F19.120	F19.982	T42.4X5S
F10.930	F13.129	F14.122	F15.188	F16.283	F19.121	F19.988	T43.605A
F10.931	F13.130	F14.129	F15.19	F16.288	F19.122	F19.99	T43.605D
F10.932	F13.131	F14.13	F15.20	F16.29	F19.129	G62.1	T43.605S
F10.939	F13.132	F14.14	F15.21	F16.90	F19.130	I42.6	T43.655A
F10.94	F13.139	F14.150	F15.220	F16.91	F19.131	K29.20	T43.655D
F10.950	F13.14	F14.151	F15.221	F16.920	F19.132	K29.21	T43.655S
F10.951	F13.150	F14.159	F15.222	F16.921	F19.139	K70.0	T50.905A
F10.959	F13.151	F14.180	F15.229	F16.929	F19.14	K70.10	Y90.4
F10.96	F13.159	F14.181	F15.23	F16.94	F19.150	K70.11	Y90.5
F10.97	F13.180	F14.182	F15.24	F16.950	F19.151	K70.2	Y90.6
F10.980	F13.181	F14.188	F15.250	F16.951	F19.159	K70.30	Y90.7
F10.981	F13.182	F14.19	F15.251	F16.959	F19.16	K70.31	Y90.8
F10.982	F13.188	F14.20	F15.259	F16.980	F19.17	K70.40	Y90.9
F10.988	F13.19	F14.21	F15.280	F16.983	F19.180	K70.41	
F10.99	F13.20	F14.220	F15.281	F16.988	F19.181	K70.9	

Footnote: Codes are those used in previous research supplemented with additional drug poisoning codes based on the National Center for Health Statistics overdose code list.

© 2026 Doran KM et al. *JAMA Network Open.*

eFigure 1. Summary of Outcome Effects for Relay vs. Site-Directed Care Arms (Intent-to-Treat Analyses)

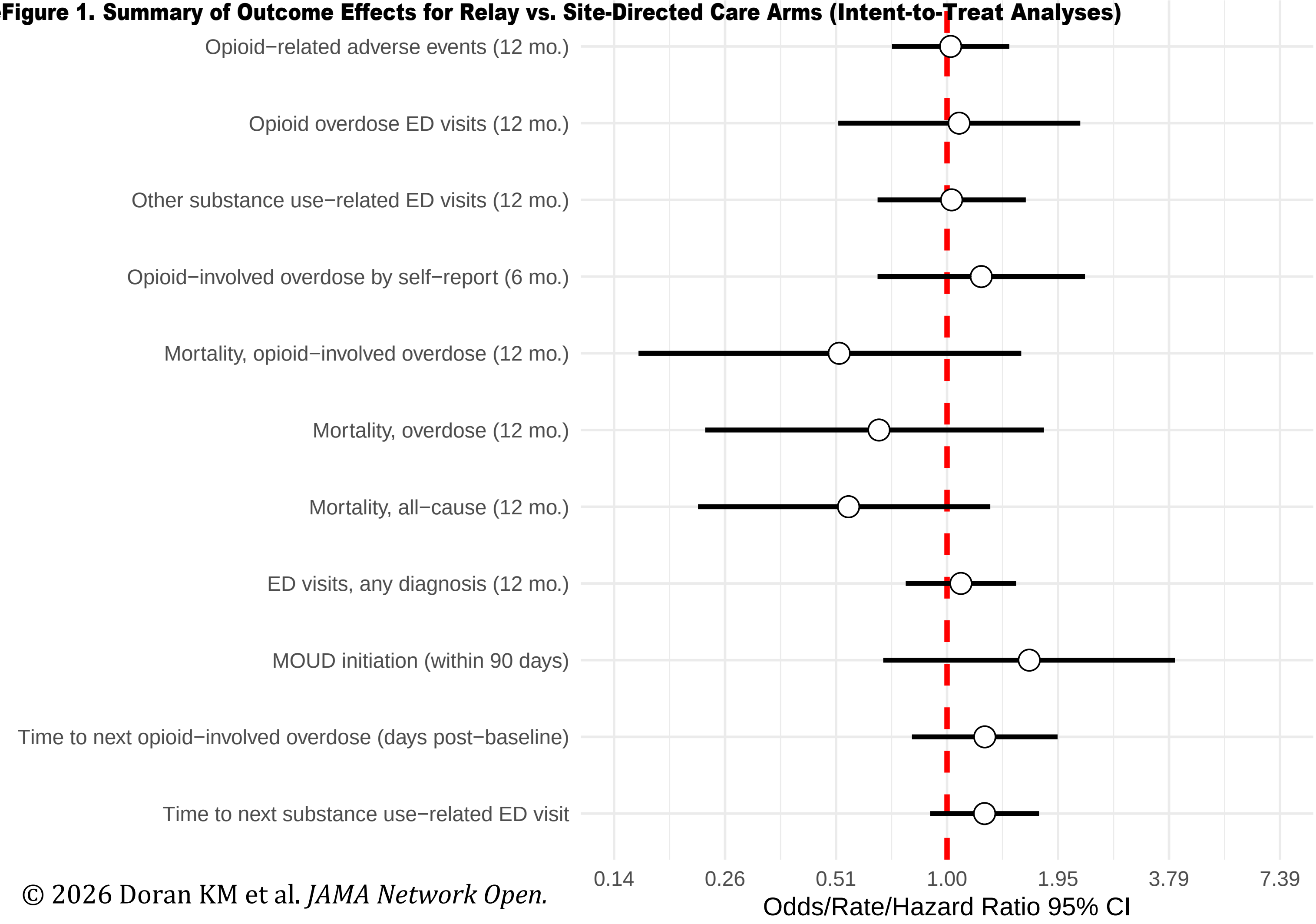

© 2026 Doran KM et al. *JAMA Network Open.*

eFigure 2. Visualization of Primary Outcome (Opioid-Related Adverse Events) for Relay and SDC Arms (Intention-to-Treat Analyses)

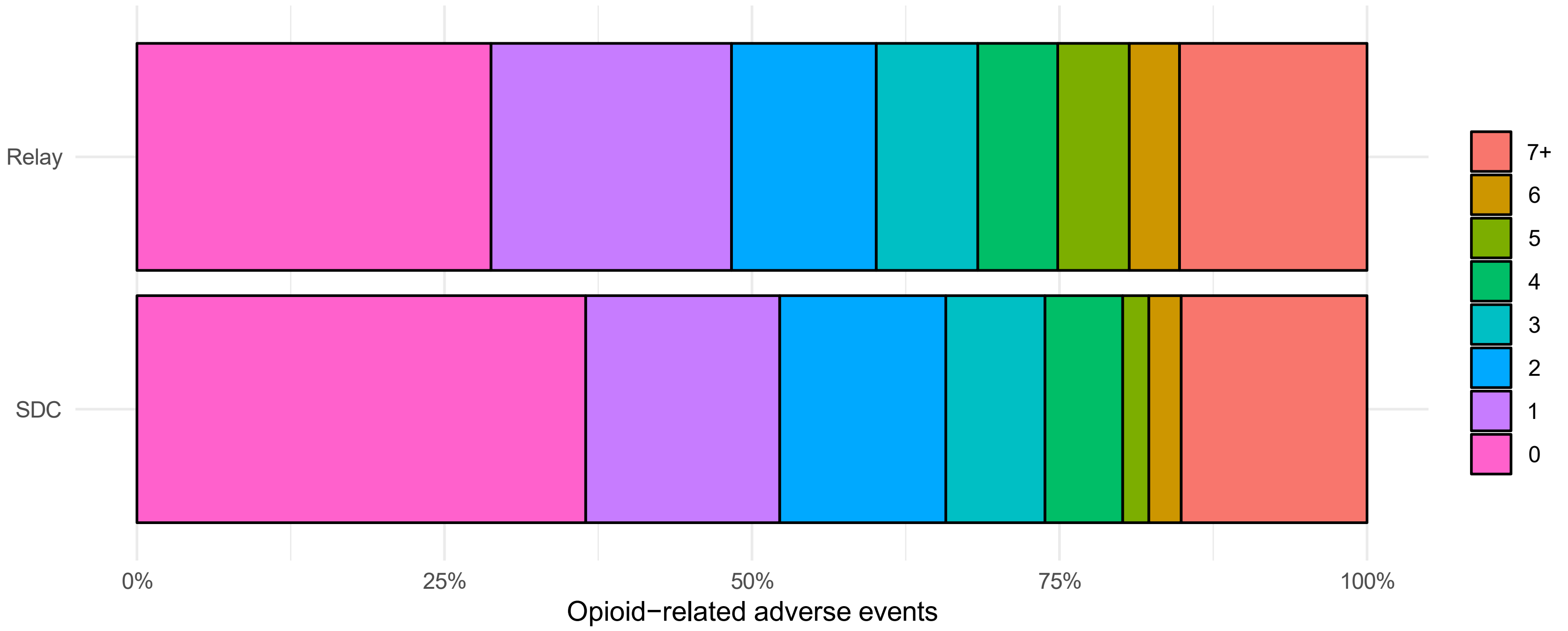

© 2026 Doran KM et al. *JAMA Network Open.*

eFigure 3. Survival Plot for Overdose Mortality Comparing Relay and SDC Arm Participants (Intention-to-Treat Analysis)

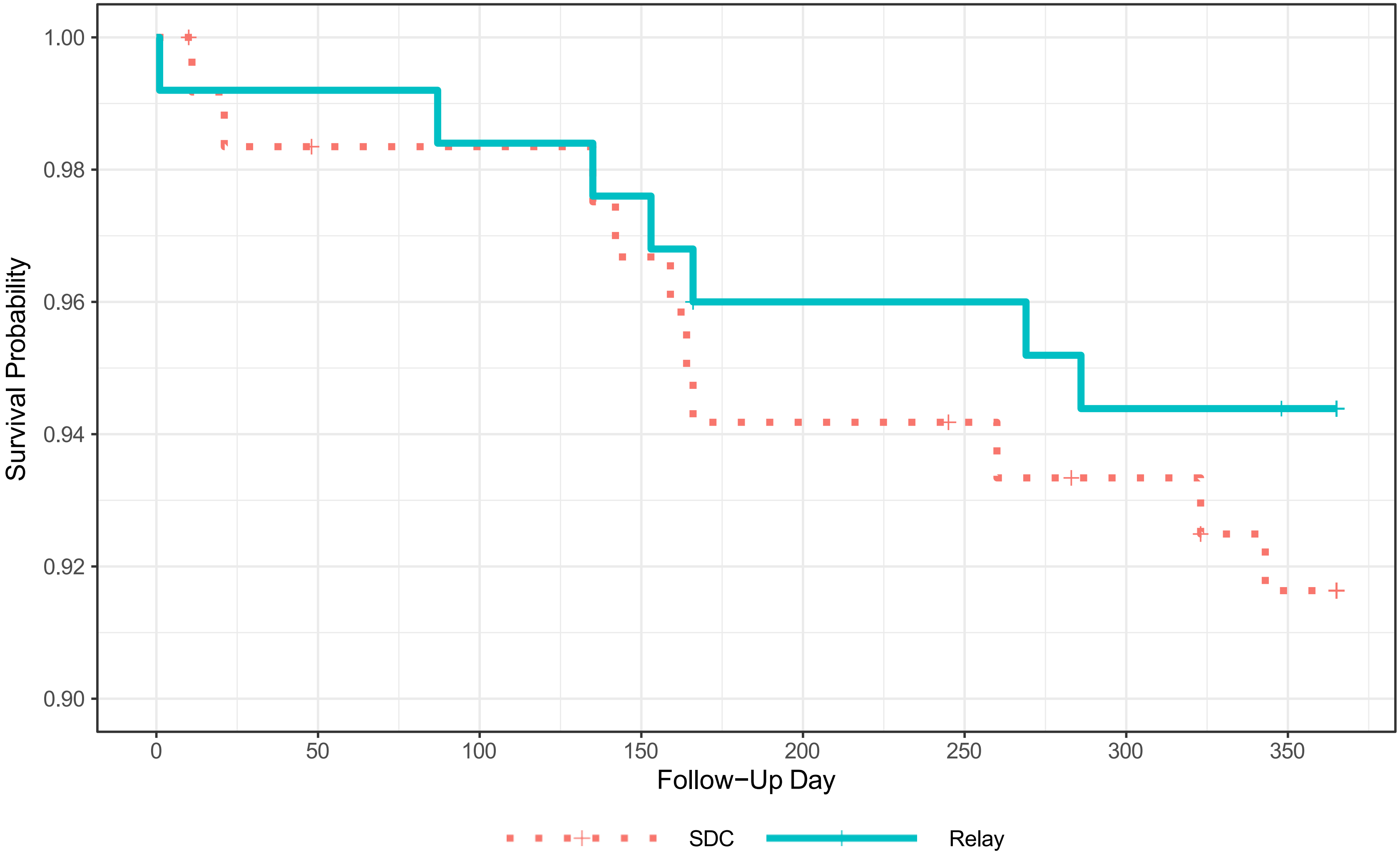

Number at Risk

SDC	122	118	118	116	113	112	110	107
Relay	125	124	123	122	119	119	117	116

© 2026 Doran KM et al. *JAMA Network Open.*

eFigure 4. Survival Plot for Opioid Overdose Mortality Comparing Relay and SDC Arm Participants (Intention-to-Treat Analysis)

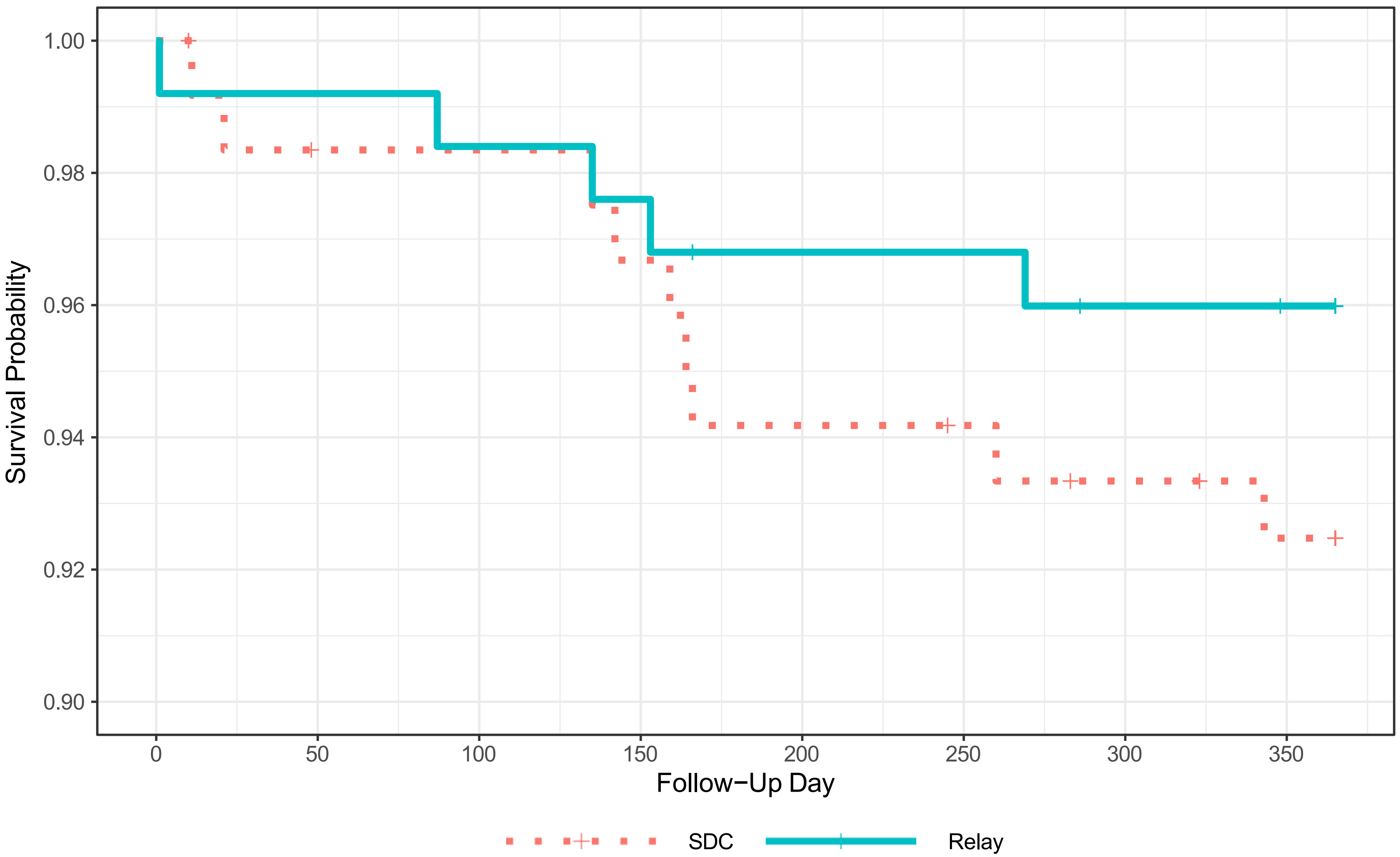

Number at Risk

SDC	122	118	118	116	113	112	110	107
Relay	125	124	123	122	119	119	117	116

© 2026 Doran KM et al. *JAMA Network Open.*

eFigure 5. Survival Plot for Time to Next Opioid-Involved Overdose Comparing Relay and SDC Arm Participants (Intention-to-Treat Analysis)

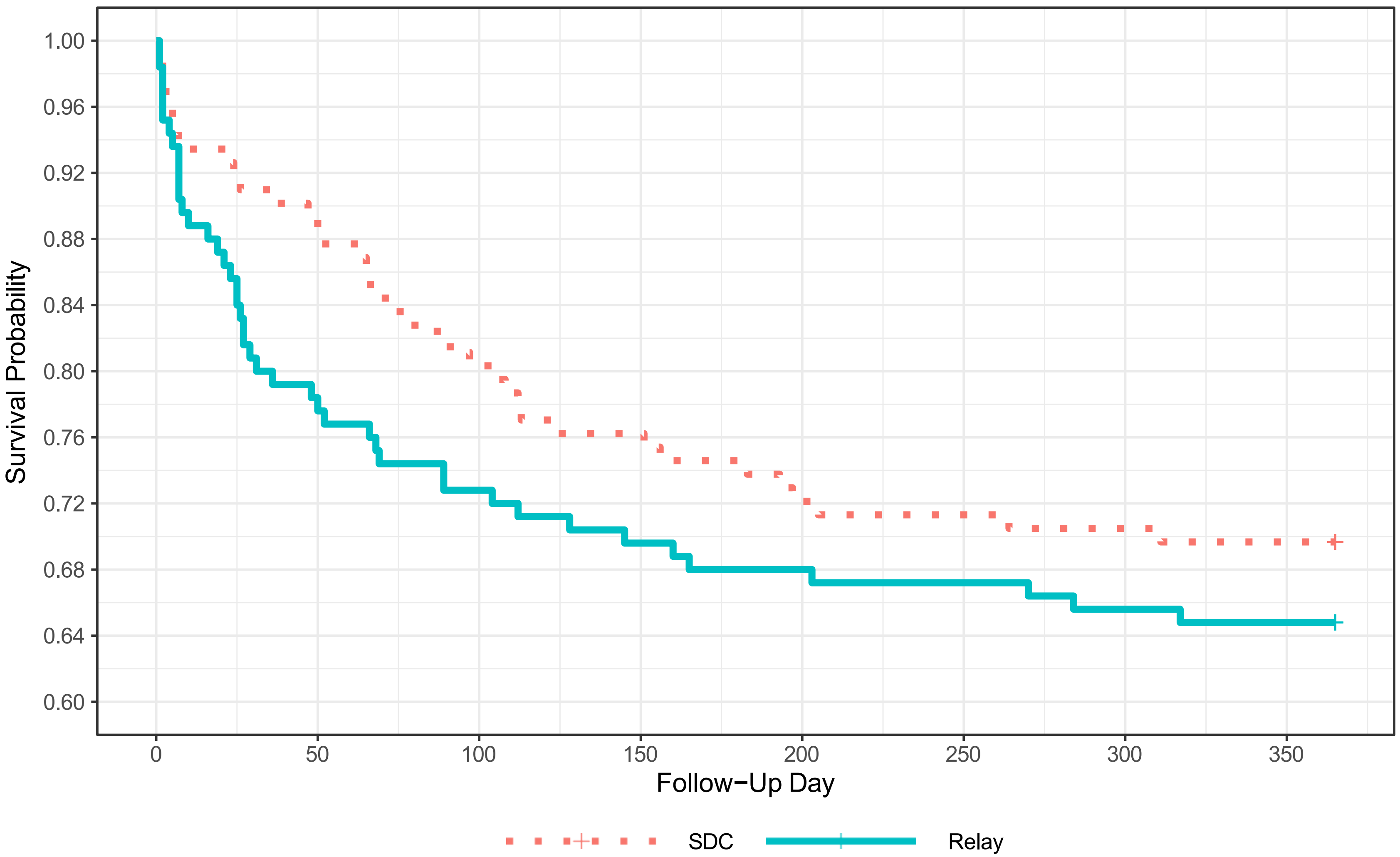

Number at Risk

SDC	122	109	98	93	88	87	86	85
Relay	125	98	91	87	85	84	82	81

© 2026 Doran KM et al. *JAMA Network Open.*

eFigure 6. Survival Plot for Time to Next Substance Use-related ED Visit Comparing Relay and SDC Arm Participants (Intention-to-Treat Analysis)

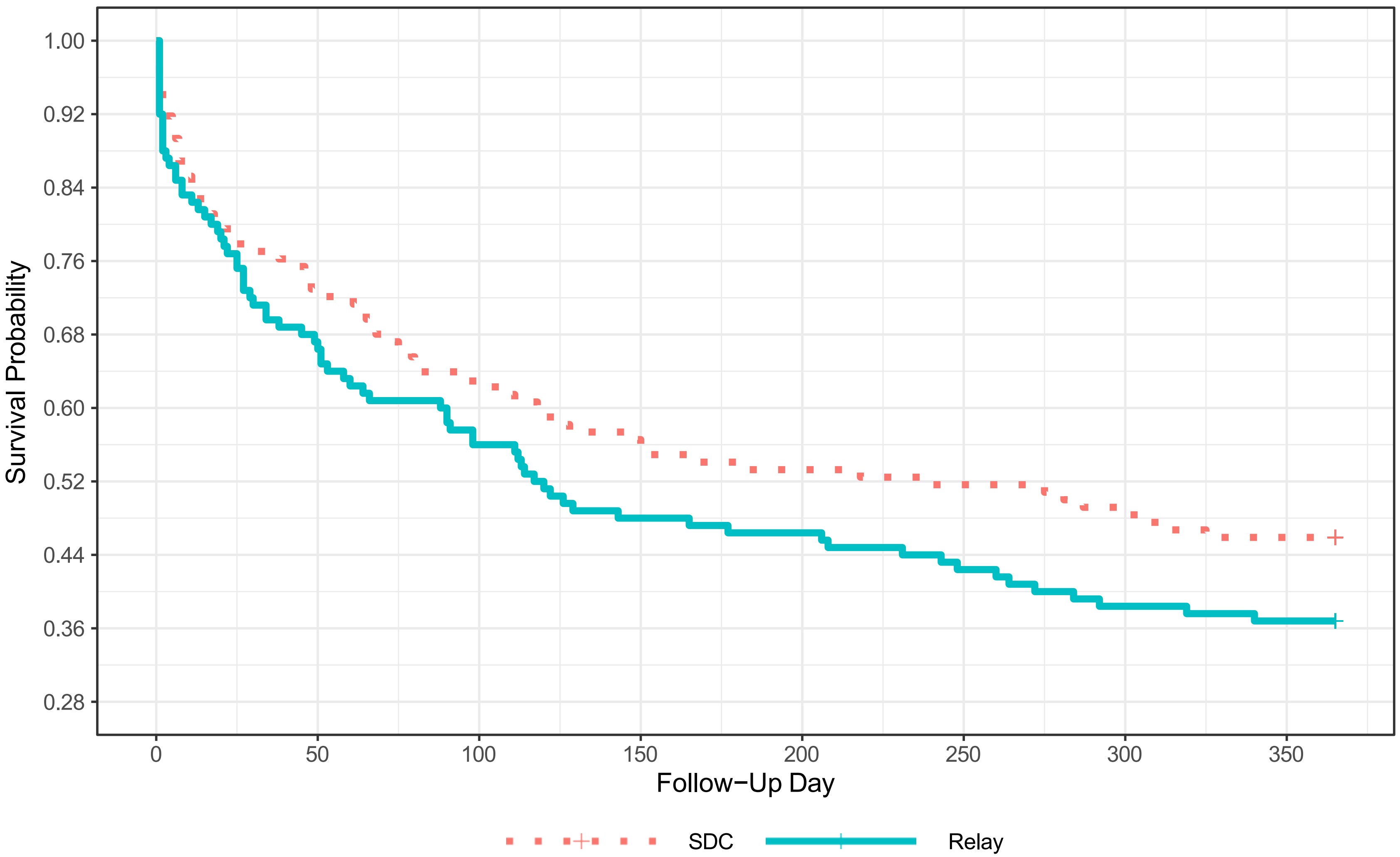

Number at Risk

SDC	122	89	76	69	65	63	59	56
Relay	125	84	70	60	58	53	48	46

© 2026 Doran KM et al. *JAMA Network Open.*
