# Supplementary material for: Peer Navigator Intervention and Opioid-Related Adverse Events for Emergency Department Patients: A Randomized Clinical Trial
Source: JAMA Netw Open. 2026 Feb 6;9(2):e2555903. doi: 10.1001/jamanetworkopen.2025.55903 (PMC12881982; doi:10.1001/jamanetworkopen.2025.55903)
Supplement: Supplement 3. — Data Sharing Statement [file jamanetwopen-e2555903-s003.pdf]

Data Sharing Statement

Doran. Peer Navigator Intervention and Opioid-Related Adverse Events for Emergency Department Patients. *JAMA Netw Open*. Published February 06, 2026.
doi:10.1001/jamanetworkopen.2025.55903

Data
Additional Information: ClinicalTrials.gov NCT04317053
Data available: Yes
Data types: Deidentified participant data, Data dictionary
How to access data: Upon reasonable request, we will provide a limited research dataset consisting of deidentified data collected directly for the purposes of this study to qualified investigators under a Data Use Agreement (DUA) that will require approval of the study principal investigators and each of the primary research team participating agencies. Please contact the corresponding author (kelly.doran@nyulangone.org) for additional details and requirements that must be met prior to sharing of data.
When available: With publication

Supporting Documents
Document types: Informed consent form
How to access documents: Please contact the corresponding author (kelly.doran@nyulangone.org) with any requests.
When available: With publication

Additional Information
Who can access the data: Upon reasonable request, we will provide the research dataset to qualified investigators meeting requirements for a Data Use Agreement (DUA) further described below.
Types of analyses: The data may only be used for qualified research purposes and not to identify any individual participant.
Mechanisms of data availability: Upon reasonable request, we will provide the research dataset to qualified investigators under a Data Use Agreement (DUA) that will require approval of the study principal investigators and each of the primary research team participating agencies (NYU, DOHMH). The DUA will be required to include: (1) a commitment to using the data only for research purposes and not to identify any individual participant; (2) a commitment to securing the data using appropriate computer technology; (3) a commitment to destroying or returning the data after analyses are completed; (4) reporting responsibilities; (5) restrictions on redistribution of the data to third parties or use for commercial purposes; (6) appropriate human subjects and institutional approvals on the part of the collaborator; and (7) proper acknowledgement of the data resources. The research dataset to be provided will include only that data collected directly for the study (i.e., study questionnaire data from baseline and follow-up interviews). Data from other sources (e.g., mortality data from the NYC Health Department's Office of Vital Statistics and health care utilization data from NYC Regional Health Information Organization (RHIOs)) is governed by separate regulations and cannot be shared with other researchers by the study team.
